# Global, regional, and national burden and trends of HIV/AIDS among women of childbearing age from 1990 to 2021: results from Global Burden of Disease 2021

**DOI:** 10.3389/fmed.2025.1605092

**Published:** 2025-07-04

**Authors:** Mingjie Tang, Jun Shao, Yanjie Jiang, Yinghong Li, Shiwei Li, Yilin Song, Wenxuan Wu, Jiqin Tang, Zhaoming Chen

**Affiliations:** ^1^Nanjing Hospital of Chinese Medicine Affiliated to Nanjing University of Chinese Medicine, Nanjing, China; ^2^Graduate School of Nanjing University of Chinese Medicine, Nanjing, China; ^3^Xiangya School of Medicine, Central South University, Changsha, China; ^4^College of Acupuncture and Orthopedics, Hubei University of Chinese Medicine, Wuhan, China; ^5^College of Rehabilitation Medicine, Shandong University of Traditional Chinese Medicine, Jinan, China

**Keywords:** HIV, AIDS, Global Burden of Disease, GBD 2021, women of childbearing age

## Abstract

**Background:**

Human immunodeficiency virus (HIV) is one of the key factors contributing to global morbidity and mortality. Women of childbearing age (WCBA) represent a high-risk population for HIV/AIDS, presenting a substantial challenge to global public health systems. A comprehensive understanding of the AIDS burden in this demographic is essential for developing targeted screening and treatment strategies to effectively control mother-to-child transmission.

**Methods:**

Utilizing GBD 2021 data, this study systematically evaluated epidemiological trends in HIV/AIDS incidence, prevalence, mortality, and disability-adjusted life years (DALYs) among WCBA. The analysis employed a multidimensional stratification approach, examining temporal patterns, age distributions, geographic variations, and Socio-demographic Index (SDI). Additionally, a comprehensive analytical approach was employed, which included the estimated annual percentage change (EAPC), Joinpoint regression, decomposition analysis, and predictive modeling using the Nordpred method. Decomposition analysis quantified contributions from population growth, aging, and epidemiological changes.

**Results:**

From 1990 to 2021, the numbers of incidence, prevalence, mortality, and DALYs of HIV/AIDS among WCBA increased significantly. In 2021, the global age-standardized rates for incidence (ASIR), prevalence (ASPR), mortality (ASMR), and DALYs (ASDALYR) were 34.73 (95% uncertainty interval [UI]: 30.03 to 40.54) per 100,000, 830.87 (95% UI: 784.57 to 884.29) per 100,000, 13.39 (95% UI: 10.34 to 17.56) per 100,000, and 829.75 (95% UI: 658.24 to 1,063.9) per 100,000, respectively. ASIR showed a downward trend, while ASPR exhibited an upward trend. Geographically, the highest persistent burden was observed in Sub-Saharan Africa. High-middle SDI region, Eastern Europe, and Pakistan had the fastest growth in incidence. Decomposition analysis showed that the increase in HIV/AIDS indicators among WCBA was mainly caused by population, and epidemiological changes. Nordpred analysis predicts modest decline in ASIR, ASPR, and ASMR by 2046.

**Conclusion:**

From 1990 to 2021, substantial rise in incident cases, prevalent cases, mortality, and DALYs has established HIV/AIDS among WCBA as a critical global public health burden. This burden exhibits marked disparities across geographic regions, countries, and age groups. In high-burden areas, particularly Sub-Saharan Africa, public health practitioners must strengthen international cooperation and prioritize expanding HIV testing and antiretroviral therapy access, women’s empowerment, and removing socio-cultural barriers.

## 1 Introduction

Acquired Immunodeficiency Syndrome (AIDS), resulting from Human Immunodeficiency Virus (HIV) infection, presents a substantial challenge to global public health (1). HIV depletes the immune system by targeting CD4 + T-lymphocytes, resulting in a heightened susceptibility to opportunistic infections and malignant neoplasms, ultimately advancing to AIDS, which carries a significant mortality rate (2). The Global Burden of Disease 2021 (GBD 2021) estimates that there were 40 million HIV infections globally in 2021, with women representing 55% (22.1 million), a substantially higher proportion than men (3). According to the 2023 UNAIDS report (4), women and girls constituted 53% of all people living with HIV (PLWH) and accounting for 44% of incident cases reported in 2023. Although extensive access to antiretroviral therapy (ART) has resulted in a 39.7 percent reduction in HIV-related mortality since 2010, women of childbearing age (WCBA) continue to represent a significant demographic affected, with a notably high disease burden in Sub-Saharan Africa (3, 5, 6). This region comprises roughly 81.1 percent of the worldwide infected female population and demonstrates a lifetime infection probability of 8.7 percent, significantly exceeding that of other regions (3, 5). Moreover, HIV infection in women of childbearing age presents a dual risk: it jeopardizes the health of the infected individuals and heightens the likelihood of vertical transmission from mother to child, thereby exacerbating the intergenerational effects of the disease (7, 8).

It is clear that women have heightened mucosal exposure of the genital tract, making them more vulnerable to HIV infection due to mucosal disruption during sexual intercourse (9). Moreover, immunosuppression during gestation has been shown to expedite viral replication (10). The risk of infection is further intensified by gender inequality, which hinders the ability of WCBA to negotiate protective measures in sexual conduct, particularly in Sub-Saharan Africa, where early marriages, multiple sexual partners, and sexual violence are prevalent (11, 12). The allocation of healthcare resources is globally inequitable. Although most of infected individuals receive ART, coverage is below 50 percent in areas like North Africa and the Middle East, with significant disparities in access to HIV screening and preventive treatment during pregnancy (13). Moreover, deficiencies persist in the prevention and management of mother-to-child transmission, as certain low-income nations do not ensure universal access to maternal viral load monitoring and post-exposure prophylaxis (PEP) for infants, leading to elevated rates of vertical transmission (14). The interconnected challenges of economic reliance and restricted educational achievement further hinder the ability of WCBA to access HIV prevention and care services, creating a detrimental cycle of “infection - poverty - loss of access” (15, 16). Thus, a thorough assessment of the HIV/AIDS burden among WCBA is essential to develop targeted interventions worldwide.

Despite the substantial burden of HIV/AIDS among WCBA, comprehensive assessments of its epidemiological trends and sociodemographic disparities remain scarce. A nuanced understanding of the spatiotemporal patterns of HIV/AIDS burden in this vulnerable population is critical to inform targeted interventions and advance progress toward the United Nations Sustainable Development Goals (SDGs) for maternal health. Leveraging the latest data from GBD 2021, this study systematically evaluates the global, regional, and national trajectories of HIV/AIDS among WCBA from 1990 to 2021 across four key metrics: incidence, prevalence, mortality, and disability-adjusted life years (DALYs).

## 2 Methods

### 2.1 Data source and disease definition

Compared to the GBD 2019 iteration, the GBD 2021 study has expanded and refined its data coverage and analytical priorities. This updated framework encompasses 204 countries and territories, evaluating 371 diseases and injuries from 1990 to 2021, including communicable diseases, and non-communicable diseases (17, 18). GBD 2021 stratifies populations across 25 age groups and both sexes (male, female, and combined). Notably, GBD 2021 places greater emphasis on temporal trends between 2010 and 2021, providing granular insights into the evolution of global disease epidemiology over the past decade, thereby enhancing evidence-based policymaking (17). Data for this study were extracted from the Global Health Data Exchange (GHDx) platform,^1^ focusing on four key indicators for WCBA: incidence, prevalence, mortality, and DALYs. DALYs represent the gold-standard metric for quantifying population health burdens, reflecting the total healthy life years lost due to morbidity and premature mortality (19). DALYs encompassing both years of life lost (YLLs) and years lived with disability (YLDs). The comprehensive methodological framework of GBD 2021, including case definitions and statistical modeling approaches, has been systematically documented in prior publications (20, 21). HIV/AIDS cases were identified using ICD-11 code 1C62 (22). The Socio-demographic Index (SDI), a composite metric integrating per capita income, average educational attainment, and total fertility rate among women under 25 years, was employed to categorize countries/territories into five development tiers (23): high SDI, high-middle SDI, middle SDI, low-middle SDI, and low SDI. Consistent with WHO standards, WCBA were defined as females aged 15–49 years (21). Our analysis specifically examined HIV/AIDS burden in this population, stratified into seven five-year age intervals (15–19, 20–24, 25–29, 30–34, 35–39, 40–44, and 45–49 years) across the 1990–2021 study period.

### 2.2 Statistical analysis

To mitigate bias from age-related factors and population structure, we computed the age-standardized incidence rate (ASIR), prevalence rate (ASPR), mortality rate (ASMR), and DALY rate (ASDALYR) for HIV/AIDS among WBCA, presenting the results as age-standardized rates (ASRs) per 100,000 and 95% uncertainty interval [UI]. The formula is as follows (24):


$$\frac{\sum_{i=1}^{N}\alpha_{i}W_{i}}{\sum_{i=1}^{N}W_{i}}$$

We assessed the fluctuations in the HIV/AIDS burden among WCBA from 1990 to 2021 utilizing the estimated annual percentage change (EAPC) (25). The EAPC was calculated by applying a linear regression model, using calendar year as the independent variable and the natural logarithm of ASRs as the dependent variable. The regression slope (*β*) denotes the EAPC, computed as (26):


y=α+βx+ε

EAPC=100×(exp(β)−1)

(α*_i_*: the age-specific rate for the *i*th age group, w: counts of individuals corresponding to the *i*th age group, N: total counts in the *i*th age group). A trend is deemed increasing if the EAPC exceeds 0 with a 95% confidence interval (CI) that excludes zero, decreasing if the EAPC is less than 0 with UI excluding zero, and stable if the CI encompasses zero. Spearman correlation analysis was utilized to evaluate the relationships between ASRs of HIV/AIDS burden metrics and the SDI.

We utilized Joinpoint regression (27) to identify critical temporal inflection points in HIV/AIDS trends. It is a methodological framework, which is employed to identify statistically significant inflection points in temporal trends through segmented linear regression modeling. This approach partitions longitudinal data into distinct phases characterized by differential trend patterns, where each segment is defined by an annual percentage change (APC) with 95% CI (28). The AAPC was calculated using the following formula:


$$\text{AAPC}=\{\exp(\frac{\sum w_{i}b_{i}}{\sum w_{i}})-1\}\times 100$$

(*wi*: length of each year segment, *bi*: slope coefficient for each year segment). An APC/AAPC with a 95% CI lower bound exceeding 0 signifies an upward trend; conversely, if the upper bound is less than 0, it indicates a downward trend. A 95% CI that encompasses zero indicates stability. Significance testing for inflection points was performed using the Monte Carlo permutation method with 10,000 iterations, applying a Bonferroni-adjusted significance threshold (*α* = 0.05).

Decomposition analysis (29) introduced by Das Gupta was employed to elucidate the contributions of three factors to alterations in the HIV/AIDS burden among WCBA: (1) population aging, (2) population growth, and (3) epidemiological shifts. This method measures the relative influence of each element on incidence, prevalence, mortality, and DALYs.

Utilizing the Nordpred package (30) in R, we forecasted the global burden of HIV/AIDS among WCBA from 2021 to 2046. The model utilized age-specific rates from GBD 2021 and WHO population structure data to estimate age-standardized rate trends for incidence, prevalence, mortality, and DALYs. Nordpred cohort-period framework incorporates demographic transitions, guaranteeing reliable long-term forecasts (31). All analyses were performed using R version 4.4.1. Statistical significance was defined as *p* < 0.05. Details regarding the data sources and methodologies can be found in Supplementary material.

## 3 Results

### 3.1 Global level

**Figure 1:**
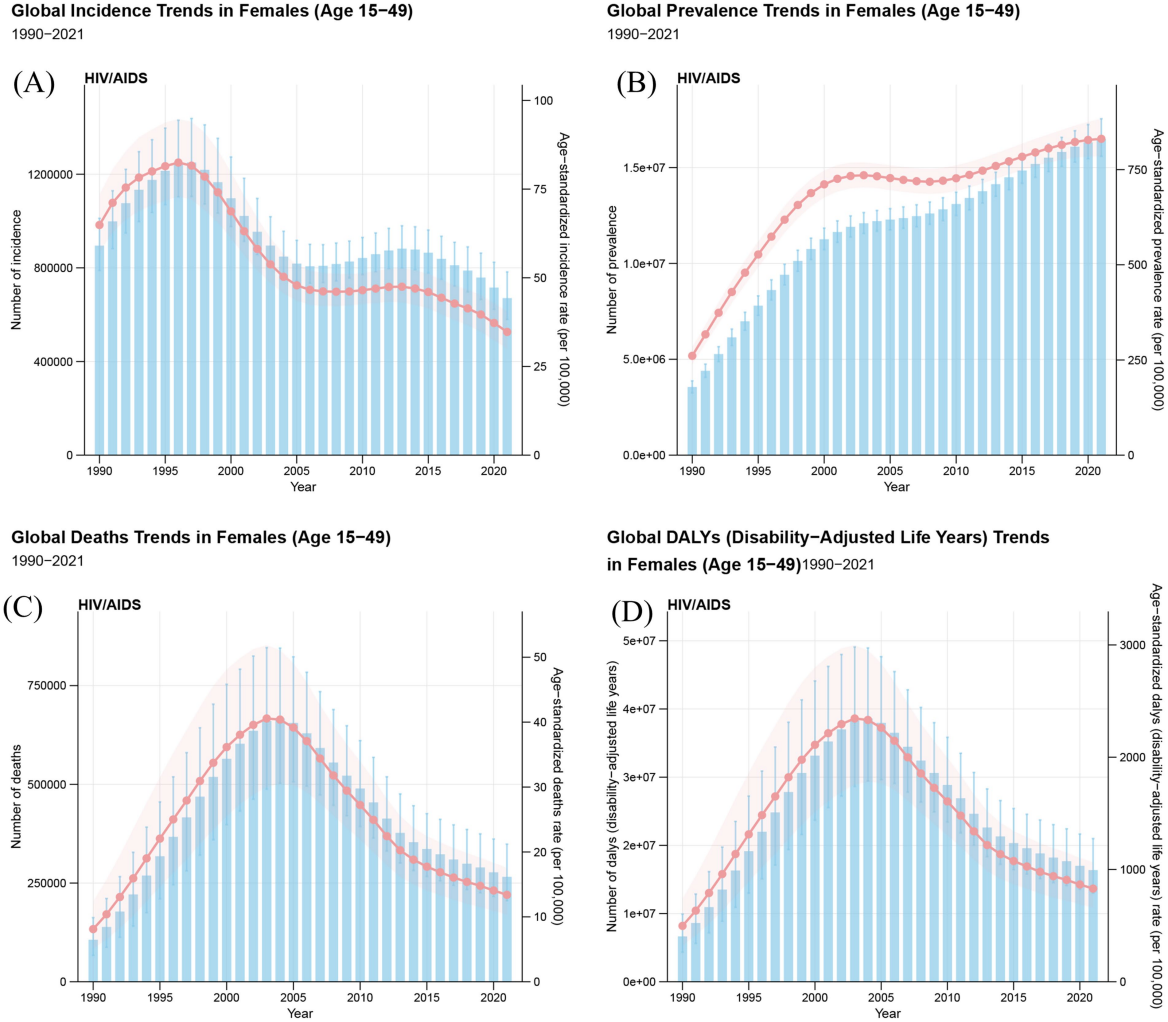
The trends in numbers and rates of incidence **(A)**, prevalence **(B)**, mortality **(C)**, and DALYs **(D)** for HIV/AIDS among women of childbearing age in global from 1990 to 2021. DALYs, disability-adjusted life years.

In 2021, global incident cases of HIV/AIDS among WCBA declined from 895,030 (95% UI: 789,222 to 1,011,444) in 1990 to 670,804 (95% UI: 579,765 to 782,487). Global prevalent cases increased from 3,554,833 (95% UI: 3,249,340 to 3,870,066) in 1990 to 16,497,874 (95% UI: 15,584,847 to 17,544,995) in 2021. From 1990 to 2021, global death cases from HIV/AIDS among WCBA increased from 106,464 (95% UI: 66,736 to 162. 356) to 265,696 (95% UI: 205,248 to 348,436). The number of DALYs increased from 6,659,152 (95% UI: 4,332,880 to 9,930,068) in 1990 to 16,378,796 (95% UI: 13,002,587 to 20,997,995) in 2021. However, the ASIR, ASPR, ASMR and ASDALYR in 2021 were 34.73 (95% UI: 30.03 to 40.54) per 100,000, 830.87 (95% UI: 784.57 to 884.29) per 100,000, 13.39 (95% UI: 10.34 to 17.56) per 100,000, and 829.75 (95% UI: 658.24 to 1,063.9) per 100,000, respectively. From 1990 to 2021, the global HIV/AIDS incidence rate among WCBA declined, with an EAPC of −2.57 (95% CI: −2.95 to −2.18). However, the prevalence of HIV/AIDS among WCBA in global increased, with an EAPC of 2.50 (95% CI: 1.84 to 3.16) (Tables 1–4; Figure 1).

**Table 1:** The incidence of HIV/AIDS cases and rates among WCBA in 1990 and 2021, and the trends from 1990 to 2021.

Location	Incidence				
	No., 1990 (95% UI)	ASIR, 1990 per 100,000 people (95% UI)	No., 2021 (95% UI)	ASIR, 2021 per 100,000 people (95% UI)	EAPC, 1990–2021 (95% CI)
Global	895,030 (789,222 to 1,011,444)	64.89 (57.14 to 73.45)	670,804 (579,765 to 782,487)	34.73 (30.03 to 40.54)	−2.57 (−2.95 to −2.18)
Low SDI	440,945 (357,077 to 530,100)	385.06 (310.38 to 465.02)	210,619 (153,432 to 288,376)	76.12 (55.64 to 103.45)	−4.67 (−4.99 to −4.35)
Low-middle SDI	347,713 (302,058 to 395,850)	122.86 (106.65 to 140.08)	201,844 (164,466 to 251,529)	39.45 (32.13 to 49.18)	−4.21 (−4.50 to −3.93)
Middle SDI	77,507 (64,626 to 91,610)	16.72 (13.99 to 19.69)	185,428 (152,558 to 220,616)	30.69 (25.25 to 36.5)	−1.11 (−2.42 to 0.21)
High-middle SDI	9,498 (7,994 to 11,398)	3.35 (2.82 to 4.03)	50,530 (32,923 to 82,623)	16.53 (10.82 to 27.06)	6.10 (5.60 to 6.6)
High SDI	18,447 (12,147 to 25,371)	8.11 (5.32 to 11.20)	21,790 (10,996 to 33,991)	9.08 (4.69 to 14.05)	0.66 (0.35 to 0.97)
Andean Latin America	503 (265 to 1,376)	5.02 (2.66 to 13.84)	2,049 (1,234 to 3,364)	11.54 (6.95 to 18.91)	1.60 (0.75 to 2.46)
Australasia	52 (35 to 73)	0.99 (0.65 to 1.38)	189 (82 to 350)	2.73 (1.22 to 5)	2.85 (2.39 to 3.31)
Caribbean	16,469 (10,502 to 24,539)	172.17 (109.71 to 257.04)	8,254 (4,161 to 14,717)	68.67 (34.66 to 122.37)	−2.20 (−2.65 to −1.75)
Central Asia	584 (382 to 869)	3.32 (2.19 to 4.92)	7,256 (3,728 to 14,244)	28.54 (14.61 to 55.93)	8.26 (7.60 to 8.91)
Central Europe	190 (119 to 280)	0.63 (0.40 to 0.94)	426 (230 to 739)	1.97 (1.05 to 3.4)	1.73 (0.71 to 2.76)
Central Latin America	3,410 (2,319 to 5,241)	8.09 (5.62 to 12.27)	6,992 (4,221 to 10,960)	10.29 (6.21 to 16.11)	1.25 (1.11 to 1.4)
Central Sub-Saharan Africa	68,971 (48,099 to 94,035)	553.21 (385.04 to 755.54)	48,790 (29,358 to 79,915)	152.52 (91.71 to 249.28)	−3.90 (−4.36 to −3.44)
East Asia	1,876 (1,126 to 3,215)	0.57 (0.34 to 0.98)	4,693 (2,108 to 8,101)	1.6 (0.72 to 2.81)	1.29 (−0.10 to 2.7)
Eastern Europe	1,914 (1,293 to 2,820)	3.4 (2.27 to 5.07)	39,756 (24,328 to 68,255)	83.59 (51.67 to 144.05)	11.53 (10.36 to 12.71)
Eastern Sub-Saharan Africa	449,160 (374,519 to 531,911)	1,013.23 (840.96 to 1,205.58)	189,069 (135,329 to 268,433)	173.87 (124.8 to 245.48)	−5.03 (−5.46 to −4.60)
High-income Asia Pacific	168 (83 to 284)	0.37 (0.18 to 0.62)	336 (189 to 514)	1.06 (0.61 to 1.6)	3.55 (3.17 to 3.93)
High-income North America	13,083 (7,435 to 19,081)	17.46 (9.86 to 25.65)	15,613 (5,951 to 26,304)	18.56 (7.26 to 31.08)	0.74 (0.37 to 1.10)
North Africa and Middle East	1,416 (489 to 4,087)	1.8 (0.62 to 5.2)	11,234 (3,715 to 34,187)	7.05 (2.33 to 21.46)	3.26 (2.85 to 3.67)
Oceania	31 (17 to 54)	1.99 (1.07 to 3.46)	2,043 (975 to 3,547)	58.76 (27.96 to 102.27)	6.73 (3.95 to 9.58)
South Asia	5,842 (3,240 to 9,940)	2.27 (1.25 to 3.87)	33,041 (18,519 to 66,653)	6.61 (3.7 to 13.34)	−1.83 (−3.97 to 0.35)
Southeast Asia	16,297 (9,622 to 24,764)	12.79 (7.64 to 19.32)	24,819 (16,909 to 36,237)	13.84 (9.39 to 20.27)	−0.93 (−1.31 to −0.54)
Southern Latin America	1,408 (1,053 to 1,809)	11.33 (8.46 to 14.57)	2,599 (1,934 to 3,406)	14.93 (11.14 to 19.54)	0.81 (0.68 to 0.95)
Southern Sub-Saharan Africa	155,000 (123,980 to 187,771)	1,120.7 (892.06 to 1,365.21)	121,228 (92,454 to 154,036)	552.09 (420.95 to 701.98)	−3.89 (−4.82 to −2.95)
Tropical Latin America	6,803 (5,101 to 8,979)	16.33 (12.35 to 21.4)	16,755 (9,002 to 29,337)	27.78 (14.84 to 48.61)	1.90 (1.45 to 2.35)
Western Europe	8,377 (6,823 to 10,332)	8.74 (7.12 to 10.79)	5,891 (4,154 to 7,888)	6.59 (4.65 to 8.81)	−0.83 (−1.06 to −0.60)
Western Sub-Saharan Africa	143,477 (114,672 to 177,125)	326.19 (260.52 to 403.71)	129,772 (104,355 to 159,876)	111.02 (89.14 to 136.96)	−4.15 (−4.45 to −3.86)

ASIR, age-standardized incidence rate; EAPC, estimated annual percentage change; WCBA, women of childbearing age.

**Table 2:** The prevalence of HIV/AIDS cases and rates among WCBA in 1990 and 2021, and the trends from 1990 to 2021.

Location	Prevalence				
	No., 1990 (95% UI)	ASPR, 1990 per 100,000 people (95% UI)	No., 2021 (95% UI)	ASPR, 2021 per 100,000 people (95% UI)	EAPC, 1990–2,021 (95% CI)
Global	3,554,833 (3,249,340 to 3,870,066)	260.91 (238.39 to 284.17)	16,497,874 (15,584,847 to 17,544,995)	830.87 (784.57 to 884.29)	2.50 (1.84 to 3.16)
Low SDI	2,067,982 (1,808,857 to 2,341,640)	1,853.22 (1,619.77 to 2,102.34)	5,569,700 (5,024,735 to 6,280,931)	2,293.68 (2,082.78 to 2,561.02)	−0.42 (−0.83 to −0.01)
Low-middle SDI	1,064,387 (937,252 to 1,191,486)	381.7 (336.04 to 427.47)	4,356,055 (4,016,853 to 4,697,371)	889.85 (820.96 to 958.68)	1.07 (0.29 to 1.86)
Middle SDI	188,954 (163,131 to 217,216)	41.65 (36.03 to 47.79)	5,624,969 (5,261,907 to 6,032,068)	870.23 (812.73 to 934.69)	7.38 (5.51 to 9.28)
High-middle SDI	55,850 (46,097 to 66,667)	19.88 (16.43 to 23.71)	565,981 (418,511 to 788,931)	167.59 (122.84 to 235.73)	6.93 (6.75 to 7.11)
High SDI	173,790 (112,581 to 251,647)	74.47 (48.10 to 107.99)	369,319 (214,412 to 542,849)	136.83 (79.73 to 200.49)	1.75 (1.58 to 1.92)
Andean Latin America	2,554 (1,632 to 3,934)	26.65 (17.35 to 40.42)	31,291 (22,166 to 44,171)	177.56 (126.04 to 250.15)	5.40 (4.61 to 6.20)
Australasia	606 (356 to 875)	11.1 (6.51 to 16.08)	2,761 (1,408 to 4,585)	35.81 (18.35 to 59.39)	3.98 (3.8 to 4.15)
Caribbean	70,735 (43,697 to 103,413)	752.45 (464.22 to 1,100.84)	143,514 (110,982 to 177,372)	1,176.61 (909.15 to 1,455.55)	0.60 (0.34 to 0.85)
Central Asia	1,277 (917 to 1,725)	7.48 (5.42 to 10.06)	24,259 (17,006 to 37,698)	94.21 (65.92 to 145.95)	7.39 (7.03 to 7.76)
Central Europe	983 (600 to 1,387)	3.2 (1.94 to 4.53)	8,548 (5,231 to 13,124)	31.18 (19.03 to 47.88)	7.16 (6.12 to 8.21)
Central Latin America	9,958 (7,031 to 13,977)	24.79 (17.68 to 34.53)	97,511 (65,506 to 139,975)	141.84 (95.1 to 203.82)	4.89 (4.43 to 5.35)
Central Sub-Saharan Africa	288,209 (218,085 to 375,516)	2,374.96 (1,801.73 to 3,095.01)	649,806 (532,708 to 787,628)	2,254.97 (1,865.53 to 2,705.14)	−1.47 (−1.88 to −1.06)
East Asia	10,557 (5,983 to 14,545)	3.27 (1.89 to 4.47)	70,329 (35,557 to 136,649)	19.87 (9.93 to 38.5)	5.70 (4.57 to 6.83)
Eastern Europe	10,524 (7,059 to 15,815)	18.26 (12.22 to 27.46)	419,095 (292,818 to 620,342)	768.52 (530.68 to 1,152.95)	13.27 (12.7 to 13.85)
Eastern Sub-Saharan Africa	1,893,605 (1,660,658 to 2,134,628)	4,401.92 (3,854.99 to 4,976.96)	5,528,688 (4,965,506 to 6,257,103)	5,956.09 (5,386.15 to 6,665.83)	−0.20 (−0.65 to 0.25)
High-income Asia Pacific	1,008 (488 to 1,655)	2.21 (1.06 to 3.61)	5,811 (3,519 to 8,354)	14.57 (8.95 to 20.94)	5.93 (5.63 to 6.24)
High-income North America	142,318 (85,878 to 214,231)	182.4 (109.54 to 275.37)	262,743 (134,575 to 411,018)	291.5 (149.54 to 455.16)	1.29 (1.14 to 1.44)
North Africa and Middle East	4,808 (2,278 to 12,841)	6.31 (3.01 to 16.82)	94,445 (49,805 to 197,239)	58.61 (30.85 to 122.7)	6.00 (5.21 to 6.80)
Oceania	91 (64 to 124)	5.89 (4.17 to 8.06)	27,681 (21,084 to 35,112)	834.27 (637.31 to 1,054.97)	13.75 (10.64 to 16.94)
South Asia	9,430 (6,055 to 14,083)	3.71 (2.38 to 5.53)	653,152 (510,828 to 884,848)	135.54 (106.28 to 182.77)	6.32 (3.22 to 9.51)
Southeast Asia	18,793 (12,705 to 26,024)	15.04 (10.25 to 20.74)	417,222 (304,805 to 573,728)	222.44 (162.02 to 306.89)	5.10 (3.54 to 6.69)
Southern Latin America	9,169 (7,608 to 10,919)	74.65 (61.95 to 88.88)	39,943 (33,314 to 47,851)	219.43 (182.79 to 263.14)	3.27 (2.96 to 3.58)
Southern Sub-Saharan Africa	457,909 (381,099 to 540,298)	3,357.73 (2,788.3 to 3,974.73)	5,331,459 (5,059,247 to 5,630,694)	24,688.66 (23,436.21 to 26,058.78)	4.92 (3.67 to 6.18)
Tropical Latin America	40,513 (27,224 to 58,350)	99.7 (67.32 to 143.45)	224,913 (130,548 to 361,706)	351.47 (203.83 to 565.27)	3.55 (3.28 to 3.82)
Western Europe	58,501 (46,041 to 72,021)	60.07 (47.27 to 74)	125,399 (92,582 to 158,530)	119.74 (88.29 to 151.47)	2.11 (1.79 to 2.43)
Western Sub-Saharan Africa	523,285 (417,244 to 646,308)	1,221.05 (974.02 to 1,510.39)	2,339,303 (2,103,547 to 2,581,389)	2,279.67 (2,056.35 to 2,506.2)	0.57 (−0.16 to 1.30)

ASPR, age-standardized prevalence rate; EAPC, estimated annual percentage change; WCBA, women of childbearing age.

**Table 3:** The mortality of HIV/AIDS cases and rates among WCBA in 1990 and 2021, and the trends from 1990 to 2021.

Location	Mortality				
	No., 1990 (95% UI)	ASMR, 1990 per 100,000 people (95% UI)	No., 2021 (95% UI)	ASMR, 2021 per 100,000 people (95% UI)	EAPC, 1990–2021 (95% CI)
Global	106,464 (66,736 to 162,356)	8.12 (5.07 to 12.36)	265,696 (205,248 to 348,436)	13.39 (10.34 to 17.56)	−0.34 (−1.99 to 1.34)
Low SDI	69,283 (40,881 to 108,386)	66.54 (39.01 to 103.73)	95,171 (67,318 to 134,722)	38.88 (27.39 to 55.37)	−3.52 (−4.94 to −2.08)
Low-middle SDI	25,165 (13,918 to 41,688)	9.50 (5.17 to 15.82)	92,438 (63,446 to 130,488)	18.92 (13.04 to 26.68)	−0.41 (−2.31 to 1.52)
Middle SDI	6,589 (5,890 to 7,584)	1.54 (1.38 to 1.78)	66,609 (60,178 to 74,474)	10.33 (9.33 to 11.56)	3.90 (1.05 to 6.83)
High-middle SDI	1,738 (1,702 to 1,774)	0.63 (0.62 to 0.64)	9,695 (9,245 to 10,697)	2.78 (2.65 to 3.08)	4.67 (4.16 to 5.17)
High SDI	3,548 (3,544 to 3,553)	1.49 (1.49 to 1.5)	1,594 (1,587 to 1,603)	0.58 (0.58 to 0.59)	−5.16 (−5.81 to −4.50)
Andean Latin America	79 (78 to 84)	0.94 (0.93 to 0.99)	541 (490 to 636)	3.08 (2.78 to 3.62)	4.33 (2.33 to 6.38)
Australasia	8 (8 to 8)	0.14 (0.14 to 0.14)	8 (8 to 8)	0.1 (0.1 to 0.1)	−2.84 (−3.73 to −1.93)
Caribbean	2,684 (1,464 to 4,591)	29.92 (16.35 to 51.1)	2,458 (1,476 to 3,850)	20.07 (12.02 to 31.45)	−2.90 (−4.00 to −1.77)
Central Asia	115 (115 to 115)	0.74 (0.74 to 0.74)	409 (408 to 410)	1.6 (1.59 to 1.6)	2.02 (1.51 to 2.53)
Central Europe	62 (61 to 62)	0.2 (0.2 to 0.2)	80 (80 to 81)	0.3 (0.3 to 0.3)	0.88 (−0.16 to 1.94)
Central Latin America	744 (743 to 744)	1.99 (1.99 to 2)	2,098 (2,095 to 2,101)	3.04 (3.04 to 3.04)	1.46 (0.89 to 2.02)
Central Sub-Saharan Africa	8,868 (5,102 to 14,670)	79.01 (45.74 to 129.66)	17,529 (11,314 to 25,654)	61.78 (40.46 to 89.15)	−2.43 (−3.71 to −1.13)
East Asia	282 (18 to 522)	0.09 (0.01 to 0.17)	3,672 (2,600 to 4,939)	0.97 (0.69 to 1.28)	6.38 (5.20 to 7.57)
Eastern Europe	744 (743 to 745)	1.27 (1.27 to 1.27)	7,304 (7,285 to 7,323)	12.74 (12.71 to 12.77)	8.71 (8.04 to 9.39)
Eastern Sub-Saharan Africa	60,093 (35,009 to 95,651)	152.16 (87.37 to 242.41)	88,028 (60,151 to 128,830)	93.14 (63.09 to 137.58)	−3.68 (−5.23 to −2.11)
High-income Asia Pacific	6 (6 to 6)	0.01 (0.01 to 0.01)	13 (13 to 13)	0.03 (0.03 to 0.03)	1.55 (0.75 to 2.36)
High-income North America	2,749 (2,745 to 2,753)	3.44 (3.44 to 3.45)	823 (823 to 824)	0.9 (0.9 to 0.9)	−6.63 (−7.39 to −5.87)
North Africa and Middle East	204 (123 to 435)	0.29 (0.18 to 0.61)	3,947 (2,318 to 7,945)	2.44 (1.43 to 4.92)	5.98 (4.81 to 7.16)
Oceania	7 (6 to 8)	0.5 (0.46 to 0.56)	328 (162 to 599)	9.96 (4.93 to 18.19)	9.14 (5.66 to 12.72)
South Asia	49 (13 to 120)	0.02 (0.01 to 0.05)	16,626 (9,725 to 29,771)	3.47 (2.04 to 6.18)	11.19 (5.75 to 16.9)
Southeast Asia	2,570 (2,509 to 2,640)	2.18 (2.13 to 2.24)	6,662 (5,483 to 8,433)	3.53 (2.91 to 4.47)	0.89 (−0.78 to 2.58)
Southern Latin America	126 (126 to 126)	1.04 (1.04 to 1.04)	488 (486 to 490)	2.66 (2.65 to 2.67)	1.72 (0.61 to 2.85)
Southern Sub-Saharan Africa	9,286 (5,174 to 15,575)	72.26 (39.38 to 122.55)	52,654 (44,513 to 64,593)	242.37 (204.53 to 297.96)	1.03 (−1.7 to 3.83)
Tropical Latin America	1,179 (1,178 to 1,180)	3.08 (3.08 to 3.08)	3,251 (3,245 to 3,258)	5 (4.99 to 5.01)	−0.26 (−1.15 to 0.63)
Western Europe	1,237 (1,235 to 1,238)	1.26 (1.26 to 1.26)	423 (422 to 423)	0.4 (0.4 to 0.4)	−6.25 (−7.11 to −5.38)
Western Sub-Saharan Africa	15,373 (8,338 to 25,735)	38.88 (20.98 to 64.91)	58,355 (39,607 to 84,420)	57.78 (39.67 to 82.7)	−0.96 (−2.6 to 0.70)

ASMR, age-standardized mortality rate; EAPC, estimated annual percentage change; WCBA, women of childbearing age.

**Table 4:** The DALYs of HIV/AIDS cases and rates among WCBA in 1990 and 2021, and the trends from 1990 to 2021.

Location	DALYs				
	No., 1990 (95% UI)	ASDALYR, 1990 per 100,000 people (95% UI)	No., 2021 (95% UI)	ASDALYR, 2021 per 100,000 people (95% UI)	EAPC, 1990–2021 (95% CI)
Global	6,659,152 (4,332,880 to 9,930,068)	497.86 (322.95 to 741.81)	16,378,796 (13,002,587 to 20,997,995)	829.75 (658.24 to 1,063.9)	−0.27 (−1.84 to 1.34)
Low SDI	4,296,830 (2,643,840 to 6,612,569)	3,993.62 (2,439.79 to 6,129.65)	5,910,328 (4,341,457 to 8,126,561)	2,351.67 (1,723.98 to 3,247.49)	−3.46 (−4.8 to −2.10)
Low-middle SDI	1,626,302 (961,045 to 2,597,193)	596.27 (347.01 to 958.11)	5,523,174 (3,915,776 to 7,638,397)	1,119.79 (796.64 to 1,545.99)	−0.52 (−2.33 to 1.32)
Middle SDI	411,167 (368,688 to 469,533)	93.84 (84.13 to 107.09)	4,258,659 (3,865,430 to 4,737,692)	666.7 (604.97 to 742.39)	4.07 (1.33 to 6.90)
High-middle SDI	104,061 (101,134 to 107,022)	37.29 (36.22 to 38.36)	563,186 (523,609 to 624,640)	164.81 (153.14 to 183.34)	4.69 (4.20 to 5.18)
High SDI	212,181 (204,806 to 222,419)	89.64 (86.49 to 94.03)	111,855 (97,518 to 130,331)	41.61 (36.34 to 48.4)	−4.46 (−5.05 to −3.86)
Andean Latin America	4,830 (4,645 to 5,124)	55.08 (53.14 to 58.24)	32,512 (29,640 to 37,392)	184.4 (168.03 to 212.25)	4.23 (2.27 to 6.22)
Australasia	502 (467 to 550)	9.18 (8.54 to 10.05)	633 (483 to 898)	8.21 (6.28 to 11.6)	−1.80 (−2.51 to −1.08)
Caribbean	162,799 (90,033 to 275,069)	1,773.87 (983.1 to 2,993.96)	146,185 (90,034 to 224,620)	1,197.7 (736.09 to 1,841.65)	−2.82 (−3.88 to −1.74)
Central Asia	6,578 (6,509 to 6,660)	41.12 (40.72 to 41.6)	23,955 (23,021 to 25,432)	93.52 (89.89 to 99.23)	2.16 (1.66 to 2.67)
Central Europe	3,663 (3,584 to 3,756)	11.89 (11.64 to 12.19)	5,159 (4,785 to 5,746)	19.87 (18.5 to 21.98)	1.22 (0.19 to 2.26)
Central Latin America	43,158 (42,674 to 43,826)	111.84 (110.64 to 113.51)	122,352 (118,374 to 128,108)	177.8 (172.01 to 186.18)	1.54 (0.95 to 2.12)
Central Sub-Saharan Africa	548,900 (326,281 to 888,900)	4,712.31 (2,813.28 to 7,580.66)	1,046,560 (695,700 to 1,512,166)	3,593.08 (2,421.96 to 5,119.17)	−2.48 (−3.69 to −1.25)
East Asia	17,269 (2,246 to 30,456)	5.52 (0.68 to 9.73)	202,974 (143,881 to 273,402)	54.92 (39.25 to 72.86)	6.33 (5.19 to 7.48)
Eastern Europe	42,210 (41,576 to 43,396)	72.24 (71.14 to 74.26)	422,893 (405,830 to 448,362)	755.69 (724.62 to 802.67)	8.87 (8.20 to 9.55)
Eastern Sub-Saharan Africa	3,759,617 (2,294,461 to 5,855,714)	9,129.64 (5,490.44 to 14,246.57)	5,508,246 (3,925,446 to 7,786,671)	5,656.98 (4,009.07 to 8,053.99)	−3.60 (−5.07 to −2.12)
High-income Asia Pacific	445 (376 to 533)	0.97 (0.82 to 1.16)	1,146 (932 to 1,447)	2.8 (2.27 to 3.54)	2.46 (1.81 to 3.12)
High-income North America	164,336 (157,828 to 173,559)	206.38 (198.05 to 218.22)	62,269 (51,297 to 77,150)	68.87 (56.74 to 85.29)	−5.70 (−6.36 to −5.04)
North Africa and Middle East	12,204 (7,532 to 25,750)	16.82 (10.52 to 34.96)	225,249 (134,080 to 451,067)	139.28 (82.74 to 279.47)	5.90 (4.75 to 7.06)
Oceania	408 (375 to 463)	28.25 (26.11 to 31.92)	20,671 (11,534 to 35,212)	619.33 (345.81 to 1,053.89)	9.24 (5.82 to 12.77)
South Asia	4,408 (2,041 to 8,637)	1.76 (0.81 to 3.48)	970,048 (583,004 to 1,720,998)	200.71 (121.13 to 355.01)	10.17 (5.14 to 15.44)
Southeast Asia	153,232 (149,427 to 157,545)	127.53 (124.41 to 131.06)	406,855 (342,872 to 496,087)	217.28 (183.21 to 264.7)	0.88 (−0.75 to 2.53)
Southern Latin America	7,828 (7,425 to 8,409)	64.1 (60.81 to 68.84)	29,139 (27,806 to 31,105)	160.34 (153.02 to 171.12)	1.59 (0.52 to 2.68)
Southern Sub-Saharan Africa	617,060 (373,481 to 988,752)	4,628.07 (2,746.81 to 7,503.29)	3,480,002 (2,991,945 to 4,152,683)	15,941.34 (13,688.03 to 19,052.23)	1.25 (−1.33 to 3.89)
Tropical Latin America	72,533 (70,610 to 75,229)	184.77 (179.98 to 191.61)	190,551 (180,614 to 205,022)	296.12 (280.61 to 318.72)	−0.30 (−1.16 to 0.57)
Western Europe	76,680 (74,625 to 79,268)	78.33 (76.23 to 80.98)	31,643 (27,664 to 36,346)	30.59 (26.79 to 35.08)	−5.42 (−6.24 to −4.58)
Western Sub-Saharan Africa	960,492 (547,320 to 1,570,174)	2,330.53 (1,318.94 to 3,802.78)	3,449,756 (2,399,779 to 4,909,364)	3,316.6 (2,334.67 to 4,668.28)	−1.03 (−2.60 to 0.57)

ASDALYR, age-standardized DALY rate; DALYs, age-standardized disability-adjusted life years; EAPC, estimated annual percentage change; WCBA, women of childbearing age.

### 3.2 SDI regional level

**Figure 2:**
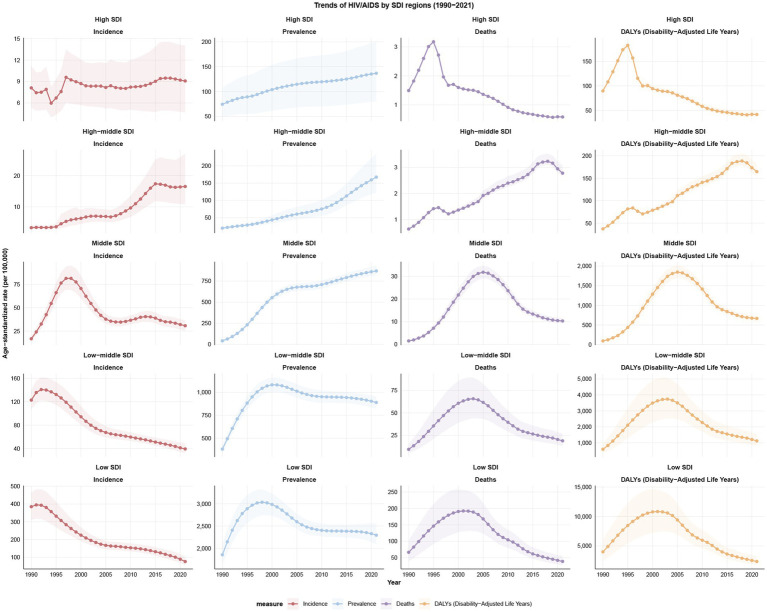
Trends of incidence, prevalence, mortality, and DALYs for HIV/AIDS among women of childbearing age by SDI regions from 1990 to 2021; DALYs, disability-adjusted life years; SDI, Socio-Demographic Index.

In 2021, the highest numbers of HIV/AIDS incidence, mortality and DALYs in WCBA globally were recorded in the low SDI region at 210,619 (95% UI: 153,432 to 288,376), 95,171 (95% UI: 67,318 to 134,722) and 5,910,328 (95% UI: 4,341,457 to 8,126,561), respectively. However, middle SDI region had the highest 2021 prevalence of all SDI regions at 5,624,969 (95% UI: 5,261,907 to 6,032,068). In 2021, low SDI region exhibited the highest ASIR, ASPR, ASMR, and ASDALYR at 76.12 (95% UI: 55.64 to 103.45), 2,293.68 (95% UI: 2,082.78 to 2,561.02), 38.88 (95% UI: 27.39 to 55.37), and 2,351.67 (95% UI: 1,723.98 to 3,247.49) per 100,000, respectively. From 1990 to 2021, incidence rates in high SDI and high-middle SDI regions demonstrated an upward trend, with the most significant increase occurring in high-middle SDI region, exhibiting an EAPC of 6.10 (95% CI: 5.60 to 6.60). Conversely, incidence rates in low-middle SDI and low SDI locations exhibited a downward trend, with the most pronounced decrease occurring in low SDI region, exhibiting an EAPC of −4.67 (95% CI: −4.99 to −4.35). The prevalence of most SDI regions exhibited an upward trend, with the exception of low SDI, which demonstrated a downward trend, exhibiting an EAPC of −0.42 (95% CI: −0.83 to −0.01). The data further revealed a downward trend in mortality and DALY rates for high SDI and low SDI regions from 1990 to 2021, while mortality and DALYs rates in high-middle and middle SDI areas showed an upward trend. Notably, high SDI region exhibited the most significant decline in mortality and DALY rates, with EAPCs of −5.16 (95% CI: −5.81 to −4.50) and −4.46 (95% CI: −5.05 to −3.86), respectively (Tables 1–4; Figure 2; Supplementary Figure S1).

### 3.3 GBD regional level

**Figure 3:**
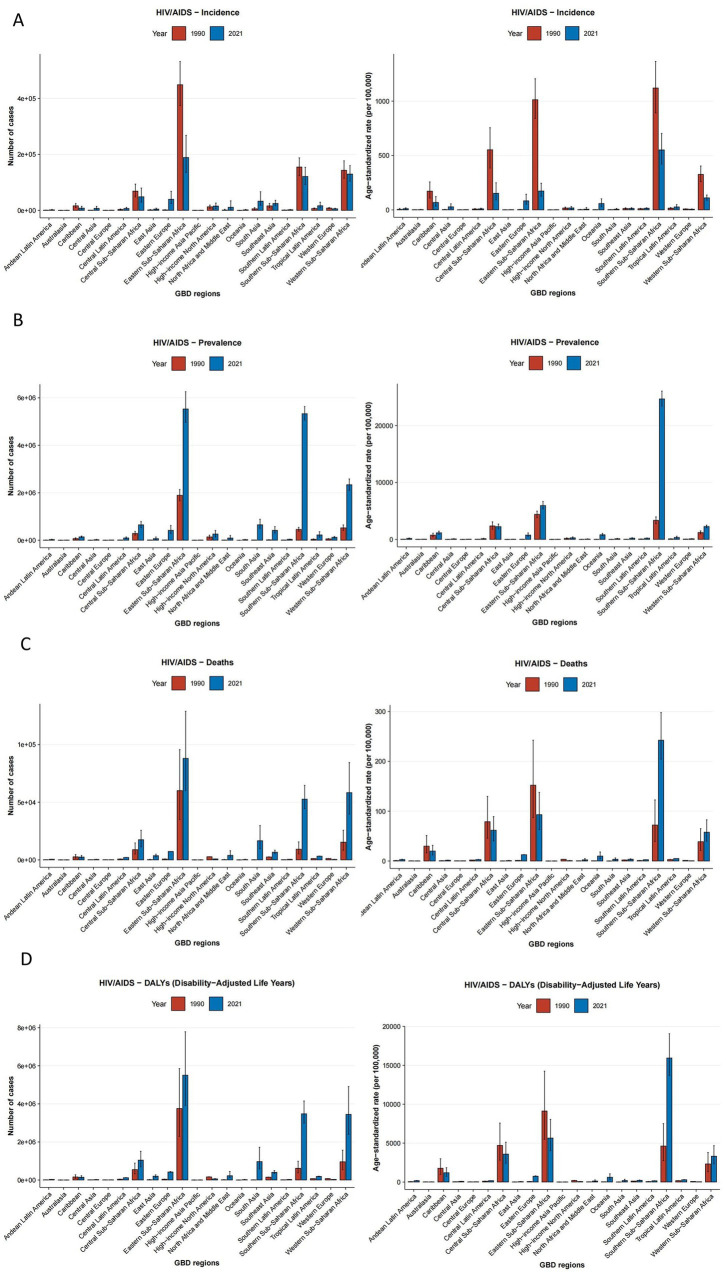
The numbers and rates of incidence **(A)**, prevalence **(B)**, mortality **(C)**, and DALYs **(D)** for HIV/AIDS among women of childbearing age by 21 regions in 1990 and 2021. DALYs, disability-adjusted life years.

In 2021, the top three regions in terms of incidence cases were Eastern Sub-Saharan Africa (189,069, 95% UI: 135,329 to 268,433), Western Sub-Saharan Africa (129,772, 95% UI: 104,355 to 159,876) and Southern Sub-Saharan Africa (121,228, 95% UI: 92,454 to 154,036). All three regions have reported more than 100,000 new cases. The highest prevalent cases of HIV/AIDS in 2021 had been recorded in Eastern Sub-Saharan Africa, with a total of 5,528,688 (95% UI: 4,965,506 to 6,257,103). The top three regions in terms of the number of deaths were Eastern Sub-Saharan Africa (88,028, 95% UI: 60,151 to 128,830), Western Sub-Saharan Africa (58,355, 95% UI: 39,607 to 84,420) and Southern Sub-Saharan Africa (52,654, 95% UI: 44,513 to 64,593) in 2021. The top three regions for DALYs were as follows: Eastern Sub-Saharan Africa (5,508,246, 95% UI: 3,925,446 to 7,786,671), Southern Sub-Saharan Africa (95% UI: 3,480,002, 2,991,945 to 4,152,683) and Western Sub-Saharan Africa (3,449,756, 95% UI: 2,399,779 to 4,909,364). In 2021, the top three regions in terms of ASIR were Southern Sub-Saharan Africa (552.09, 95% UI: 420.95 to 701.98), per 100,000, Eastern Sub-Saharan Africa (173.87, 95% UI: 124.8 to 245.48) per 100,000 and Central Sub-Saharan Africa (152.52, 95% UI: 91.71 to 249.28) per 100,000. Conversely, High-income Asia Pacific had the lowest ASIR at 1.06 (95% UI: 0.61 to 1.6) per 100,000. In 2021, Southern Sub-Saharan Africa had the highest ASPR of 24688.66 (95% UI: 23436.21 to 26058.78) per 100,000. However, high-income Asia Pacific has the lowest ASPR of 14.57 (95% UI: 8.95 to 20.94) per 100,000. By contrast, Southern Sub-Saharan Africa has the highest ASMR and ASDALYR of 242.37 (95% UI: 204.53 to 297.96) per 100,000 and 15941.34 (95% UI: 13688.03 to 19052.23) per 100,000, respectively in 2021 (Tables 1–4; Figure 3).

The region that has experienced the most significant increase in incidence over the past 32 years is Eastern Europe, with an EAPC of 11.53 (95% CI: 10.36 to 12.71). In contrast, Eastern Sub-Saharan Africa has demonstrated the largest decrease, exhibiting an EAPC of −5.03 (95% CI: −5.46 to −4.60). From 1990 to 2021, the majority of regions exhibited an upward trend in prevalence, with Oceania demonstrating the most significant increase, as indicated by an EAPC of 13.75 (95% CI: 10.64 to 16.94). Southern Sub-Saharan Africa demonstrated the largest increase in mortality and DALYs, with 11.19 (95% CI: 5.75 to 16.90) and 10.17 (95% CI: 5.14 to 15.44). This finding indicates a high burden of HIV/AIDS among WCBA in this region. High-income North America exhibited the most substantial declines in mortality and DALYs, with EAPCs of −6.63 (95% CI: −7.39 to −5.87) and −5.70 (95% CI: −6.36 to −5.04), respectively (Tables 1–4; Figure 3).

### 3.4 Countries level

**Figure 4:**
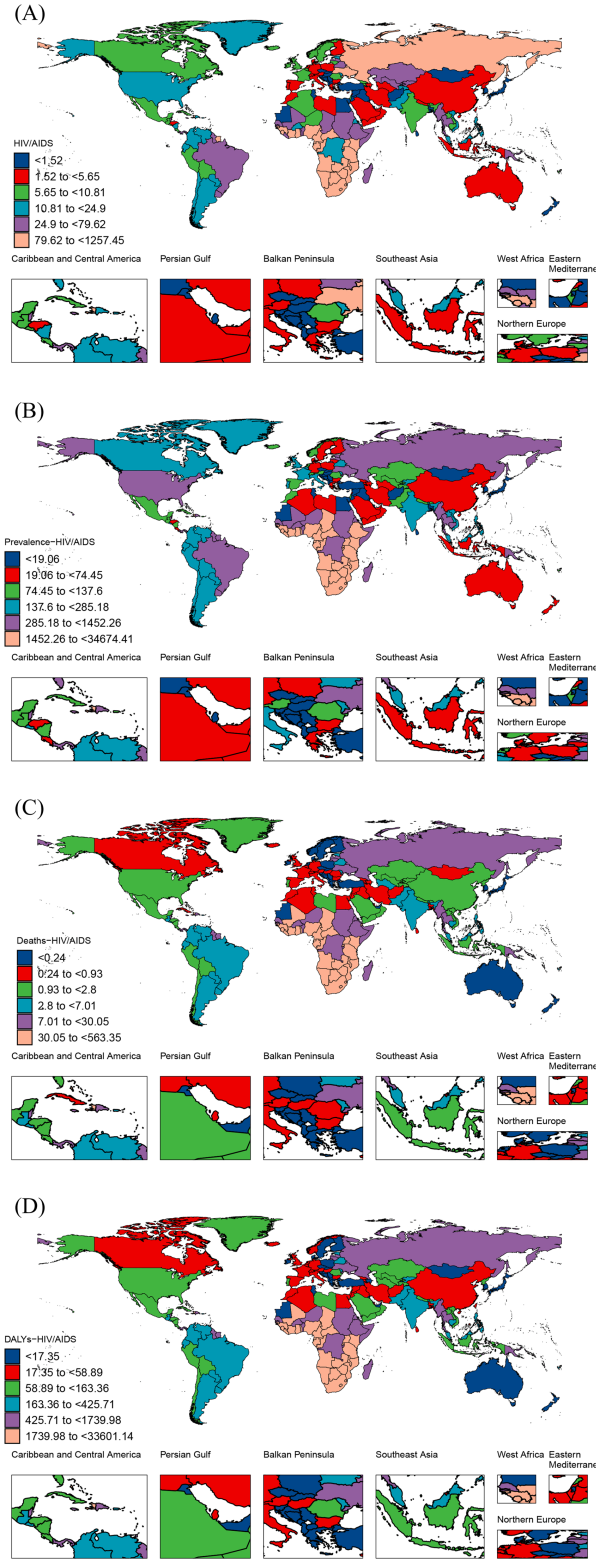
ASIR **(A)**, ASPR **(B)**, ASMR **(C)**, and ASDALYR **(D)** for HIV/AIDS among women of childbearing age across 204 countries and territories in 2021. ASIR, age-standardized incidence rate; ASPR, age-standardized prevalence rate; ASMR, age-standardized mortality rate; ASDALYR, age-standardized disability-adjusted life years rate.

In 204 countries and territories, the highest ASIR for HIV/AIDS among was observed in Equatorial Guinea (ASIR = 1217.3, 95% UI: 244.93 to 3255.72), Angola (ASIR = 431.35, 95% UI: 194.52 to 824.85) and Guinea-Bissau (ASIR = 406.88, 95% UI: 31.29 to 1149.83) per 100,000, respectively. The top three countries with the ASPR in 2021 were the Lesotho, Eswatini, and, South Africa, with rates of 34674.41 (95% UI: 29692.61 to 39180.09), 33810.75 (95% UI: 30340.38 to 37315.51), and 26684.61 (95% UI: 25064.63 to 28413.31) per 100,000, respectively. The top three with the ASMR in 2021 were the Lesotho, Eswatini, and Mozambique, with rates of 563.35 (95% UI: 299.99 to 936.66), 486.17 (95% UI: 213.16 to 947.87), and 322.18 (95% UI: 284.2 to 371.32) per 100,000, respectively. The top three countries in the 2021 ASDALYR ranking are consistent with ASMR (Figure 4; Supplementary Tables S1–S4).

From 1990 to 2021, the incidence rates were on the rise in 54.4% of countries. The greatest increase in incidence of HIV/AIDS among WCBA were in Pakistan (EAPC = 32.38, 95% CI: 27.89 to 37.03). Conversely, the country with the most significant decreases in incidence was Burundi (EAPC = -13.82, 95% CI: −8.43 to −7.26). Between 1990 and 2021, the prevalence was increasing in 83.8% of countries. The greatest increase in incidence of HIV/AIDS among WCBA was Pakistan (EAPC = 35.52, 95% CI: 32.24 to 38.88). Conversely, the largest reduction in prevalence was in Burkina Faso, with EAPC of −5.94 (95% CI: −6.18 to −5.71). From 1990 to 2021, the mortality and DALYs showed a declining trend in approximately half of the countries. The largest decline was seen in Burkina Faso, with EAPCs of −9.59 (95% CI: −10.68 to −8.48), and −9.46 (95% CI: −10.48 to −8.43). Conversely, the country with the most significant increases was Pakistan, with EAPCs of 41.48 (95% CI: 38.43 to 44.61), and 40.56 (95% CI: 37.56 to 43.61) (Figure 4; Supplementary Tables S1–S4).

### 3.5 Age patterns

**Figure 5:**
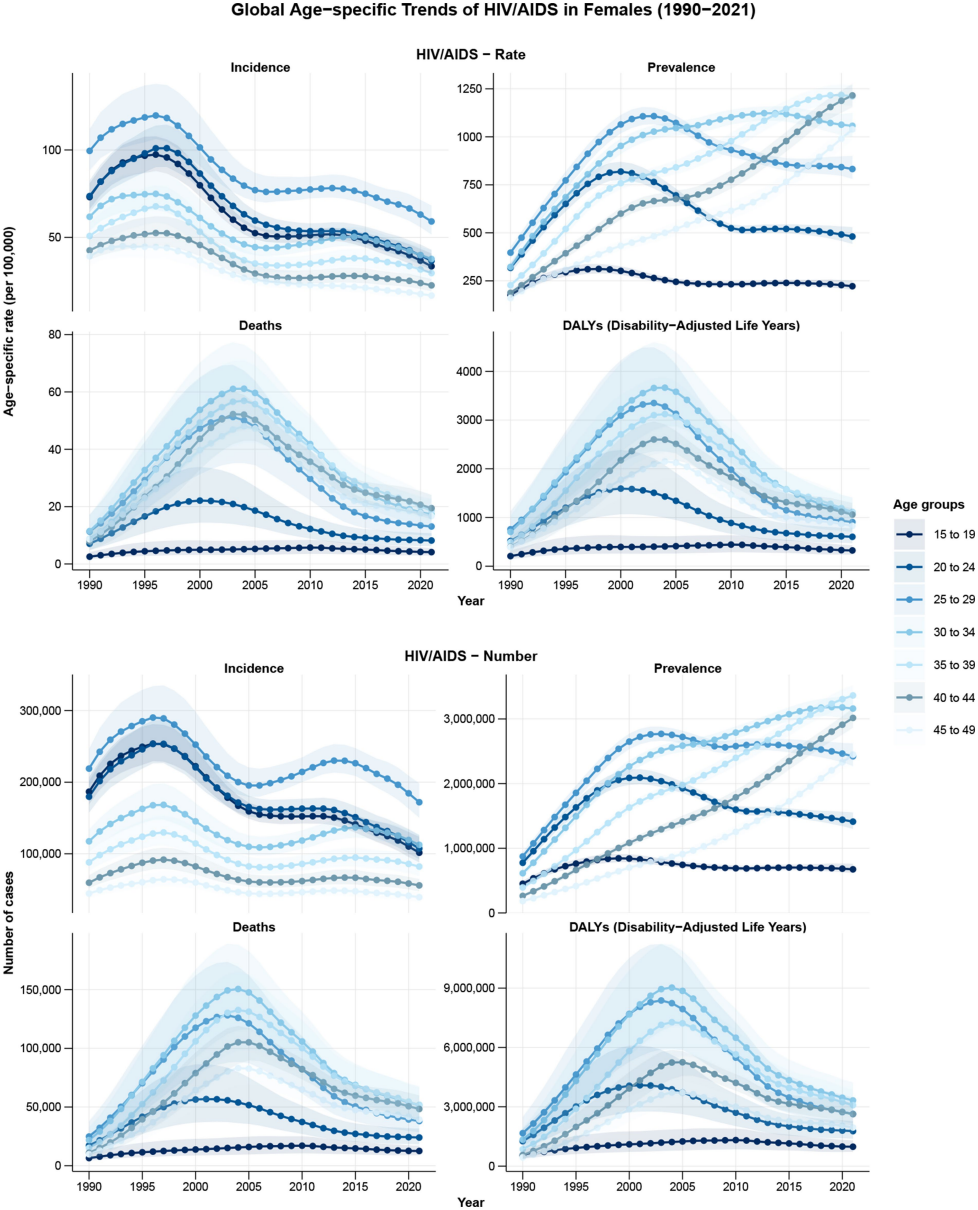
The trends of burden indicators for HIV/AIDS among women of childbearing age in different age-groups from 1990 to 2021.

In 2021, the age group with the highest incident numbers of HIV/AIDS among WCBA in global was 25–29, the age group with the highest number of prevalent cases was 35–39, and the age group with the highest number of deaths and DALYs was 30–34. In 2021, the incidence, prevalence, mortality, and DALYs of HIV/AIDS among WCBA in global exhibited a first increasing and then decreasing trend with age. The incidence of the condition peaks in the 25–29 age group, the prevalence and mortality peak in the 40–44 age group, and the DALYs peak in the 30–34 age group (Figure 5; Supplementary Figure S2).

### 3.6 The association between ASR and SDI

**Figure 6:**
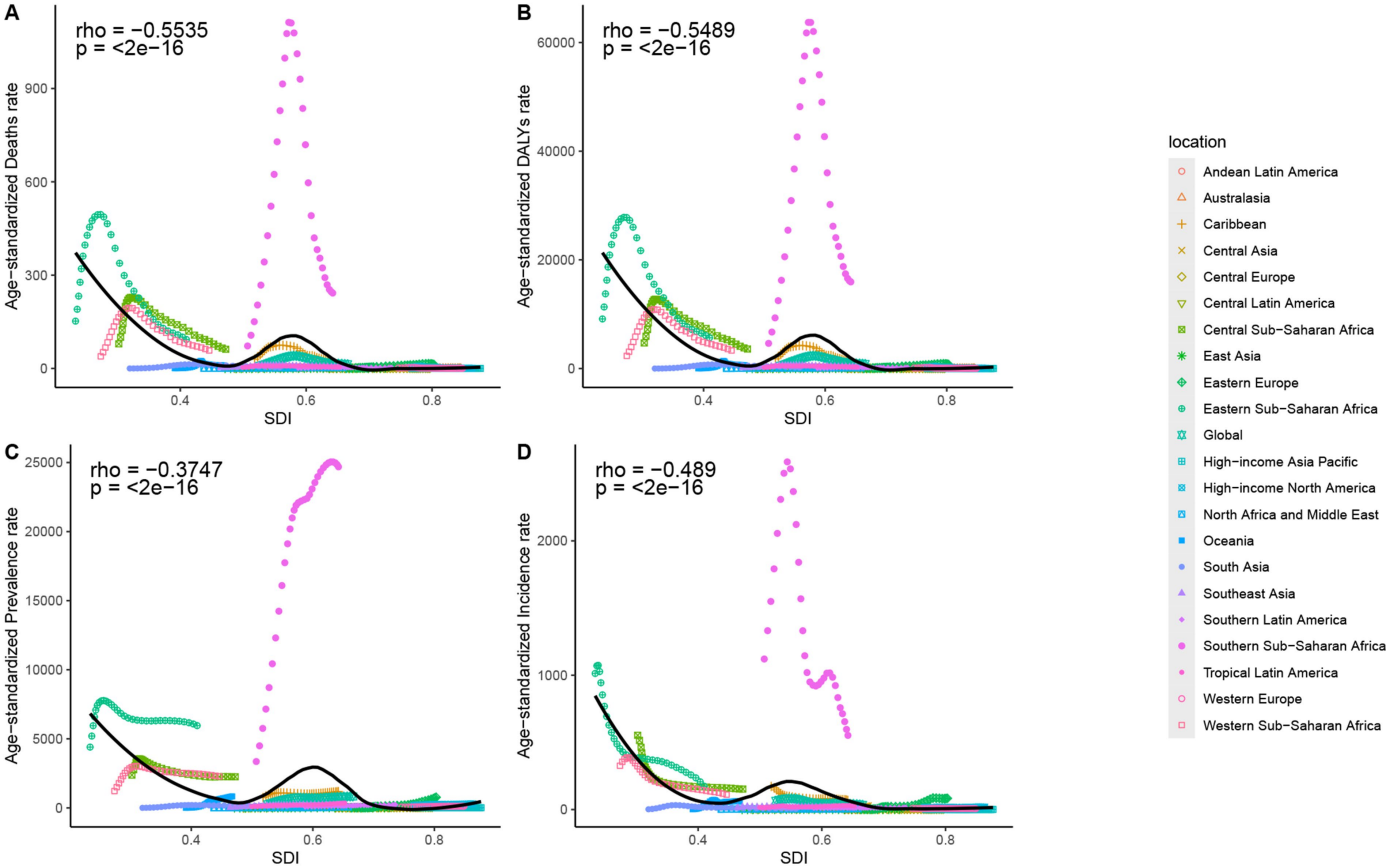
The associations between the SDI and ASMR **(A)**, ASDALYR **(B)**, ASPR **(C)**, ASIR **(D)** for HIV/AIDS among women of childbearing age across 21 regions. ASIR, age-standardized incidence rate; ASPR, age-standardized prevalence rate; ASMR, age-standardized mortality rate; ASDALYR, age-standardized disability-adjusted life years rate; SDI, Socio-Demographic Index.

From 1990 to 2021, the relationship between SDI and ASIR, ASPR, ASMR, and ASDALYR exhibited significant variations, particularly evident in Southern Sub-Saharan Africa. As SDI increases, a decrease in ASIR and ASPR is evident in most regions. ASIR in Southern Sub-Saharan Africa peaks at SDI close to 0.6 and then declines rapidly. Concurrently, ASMR and ASDALYR demonstrate a downward trend in the majority of regions, with Eastern Sub-Saharan Africa, Central Sub-Saharan Africa, and Western Sub-Saharan Africa exhibiting an initial upward trend, followed by a subsequent decrease, at SDI values less than 0.4. ASMR and ASDALYR in Southern Sub-Saharan Africa peaked at SDI close to 0.6 and then declined rapidly, while the former three showed a more moderate downward trend (Figure 6).

### 3.7 Decomposition analysis

From 1990 to 2021, the global incidence decreased significantly, with the greatest decrease occurring in low SDI region. The decline can be attributed to aging, population and epidemiological changes, which accounted for 14.6, −137.56 and 222.96% of the global decline in incidence, respectively. The impact of these various factors on the incidence of the disease varied according to geographical location. The population contribution was found to be most significant in the low SDI region, accounting for 265.46% of the total. Conversely, the Middle SDI region exhibited the most substantial increase in global prevalence. The impact of aging, demographic and epidemiological changes on the increase in global prevalence was 3.43, 25.79 and 70.78%, respectively. Globally, there was a significant increase in the number of deaths, with the largest increase recorded in the low-middle SDI region. The influence of aging, population and epidemiological changes accounted for 5.89, 41.06 and 53.05% of the global increase in deaths, respectively. A significant increase in DALYs was observed on a global scale, with the largest increase occurring in the low-middle SDI region. The same three factors accounted for 3.36, 41.75 and 54.89% of the increase in global DALYs, respectively. These findings indicate that expanding population sizes and epidemiological characteristics are significant contributors to the rising burden of disease in these regions (Supplementary Figure S3).

### 3.8 Joinpoint regression analysis

Joinpoint regression analyses demonstrated a general downward trend in ASIR (AAPC = −1.94; 95% CI: −2.05 to −1.82; *p* < 0.001) globally over the past 32 years. Conversely, the overall upward trend in ASPR (AAPC = 3.84; 95% CI: 3.79 to 3.91; *p* < 0.001) is significant. Of particular note is the downward trend in ASIR (APC = −7.09; 95% CI: −7.39 to −6.81; *p* < 0.001) from 1997 to 2005, and the subsequent downward trend in ASIR (APC = −4.52; 95% CI: −5.21 to −3.83; *p* < 0.001) from 2015 to 2021. Nevertheless, a rising trend was evident in all annual phases of ASPR, with inflection points occurring in 1992, 1995, 1998, 2001 and 2009. Global ASMR (AAPC = 1.58; 95% CI: 1.48 to 1.68; *p* < 0.001) and ASDALYR (AAPC = 1.61; 95% CI: 1.51 to 1.70; *p* < 0.001) demonstrated an overall upward trend, with the same inflection points (1994, 1999, 2003, 2006, 2015). ASMR exhibited a marked increase from 1990 to 1994 (APC = 24.14; 95% CI: 23.12 to 25.22; *p* < 0.001). However, from 2003 onwards, ASMR demonstrated a downward trend, with the most significant decline from 2006 to 2015 (APC = −8.15; 95% CI: −8.61 to −7.86; *p* < 0.001). The trend for ASDALYR was largely consistent with ASMR (Supplementary Figure S4).

### 3.9 Prediction analysis

Based on the Nordpred predictive analysis, it is projected that by 2046, the annual number of new cases will decrease to estimated 470,000 cases, while the annual number of prevalent cases will increase to estimated 18,000,000 cases. The annual number of deaths is also expected to rise, reaching estimated 210,000 cases by 2046. In 2046, the ASIR is anticipated to decline to 22.55, the ASPR to 818.46, and the ASMR to 9.77. Therefore, we observe that the burden of HIV/AIDS is gradually diminishing. However, the overall situation remains severe (Supplementary Figure S5).

## 4 Discussion

The 2021 United Nations General Assembly (UNGA) Political Declaration on HIV/AIDS (32), titled “Ending Inequalities and Getting on Track to End AIDS by 2030,” outlines ambitious targets, including reducing annual new HIV infections to <370,000 and AIDS-related deaths to <250,000 by 2025 (33). Central to this framework is addressing gender disparities, particularly reducing HIV incidence among women and ensuring 95% of women access sexual and reproductive health services by 2025 to mitigate the impact of gender-based violence on HIV transmission. However, limited studies have systematically quantified the global burden of HIV/AIDS among WCBA. This study, utilizing GBD 2021 data, provides the first comprehensive estimates of HIV/AIDS incidence, prevalence, mortality, and DALYs among WCBA over 32 years (1990–2021) and projects trends to 2046. Key findings include: first, the ASIR of HIV/AIDS among WCBA exhibited a declining trend globally, while the ASPR increased; Second, there are regional differences in the burden of HIV/AIDS among WCBA, with increasing trends in high and high-middle SDI regions and decreasing trends in middle, low-middle, and low SDI areas from 1990 to 2021. As SDI increases, ASIR and ASPR trend downward in most regions. The region with the largest increase in incidence was Eastern Europe, while the region with the largest decrease was Eastern Sub-Saharan Africa. Then, HIV/AIDS among WCBA had the highest incidence in the 25–29 year age group and the highest mortality and DALYs in the 30–34 year age group. Decomposition analyses revealed that expanding population base and epidemiological characteristics are determinants of the rising burden of HIV/AIDS. Projections show a declining trend in ASIR, ASPR and ASMR to 2046.

While a previous study (34) has examined global temporal trends in the incidence of mother-to-child transmission (MTCT) of infectious diseases among WCBA, critical gaps remain. Existing research lacks in-depth analyses of the underlying drivers of high HIV/AIDS prevalence in this population. To address these limitations, this study provides a comprehensive exploration through decomposition analysis, Joinpoint regression analysis and projections. While national data are indeed vital for informing local public health decisions, GBD-derived estimates offer a valuable complementary viewpoint. They can help fill in data gaps that may exist in certain regions or for specific sub-populations, and they also enable more straightforward comparisons across different countries and regions around the world. Furthermore, we propose evidence-based policy recommendations to guide governmental and public health agencies in designing targeted interventions. The underlying causes of the elevated prevalence of HIV/AIDS among WCBA are complex, encompassing a blend of biological, social, and economic factors. Research indicates that women have a significantly higher risk of HIV acquisition after a single exposure compared to men, due to the increased surface area of the cervicovaginal mucosa and heightened cellular immune activity in the vagina (35). Moreover, age has been recognized as a crucial determinant in the risk of HIV infection. Younger women demonstrate greater susceptibility to HIV than older women, a disparity potentially attributable to the larger expanse of vulnerable genital mucosa in the immature cervix and elevated inflammation levels in the reproductive tract among younger women, both of which are positively correlated with an increased risk of HIV infection (36, 37). In numerous areas with elevated HIV prevalence, gender inequality is widespread, and women frequently encounter obstacles in sexual relationships and struggle to reject unsafe sexual practices (38). Early marriage, multiple sexual partners, and sexual violence are widespread, significantly increasing the risk of infection (6). A study indicated that women with a history of multiple sexual partners possess an approximately 1.3-fold increased likelihood of HIV infection relative to their counterparts (6, 39). The economic difficulties faced by numerous women result in the exploitation of sexual services for essential survival resources, and the prevalence of multiple concurrent sexual partners markedly heightens the risk of exposure. The estimated prevalence of HIV among women in slum areas is 13 percent (40). Furthermore, the restricted educational achievements of WCBA directly lead to inadequate health literacy and a failure to access or understand preventive information. A study in Malawi indicated that merely 12.5 percent of women were cognizant of HIV pre-exposure prophylaxis (PrEP), with awareness significantly lower among those lacking secondary education, and substantially below that of men (41).

In 2021, the incidence, prevalence, mortality, and DALY rates were highest in low SDI region. This discovery indicates an increased prevalence of HIV/AIDS in areas marked by low SDI levels. This phenomenon is chiefly ascribed to the restricted availability of medical resources and the inadequate healthcare capacity in these areas (6). This study has identified a significant upward trend in the incidence of HIV/AIDS among WCBA, with the most pronounced increases observed in high-middle SDI region. The most credible hypothesis is that high-middle SDI districts are predominantly experiencing rapid economic development, characterized by urbanization resulting in significant population migration (e.g., rural–urban mobility). These populations frequently face a deficiency in social security, a rise in transient sexual relationships, and insufficient access to sexual health services. The influence of economic growth and social development on mitigating the disease burden is obstructed by entrenched cultural contexts and the stigma linked to HIV/AIDS among WCBA (3). From 1990 to 2021, Eastern Europe experienced the most significant rise in HIV/AIDS incidence among WCBA. The principal transmission risk has transitioned from unsafe injection practices to heterosexual contact, yet access to opioid agonist therapy and needle-syringe programs remains constrained (42). Moreover, the Russo-Ukrainian war in recent years has obstructed efforts to test and treat individuals, and the ensuing economic difficulties have forced some women to participate in high-risk sex work, which is a significant concern (43). The war in Ukraine conflict has exacerbated gaps in HIV services. As a result, projections may underestimate the burden in crisis-affected areas, reinforcing the need for adaptive health systems. However, implementing these policy recommendations in real-world settings presents significant challenges, particularly in fragile or conflict-affected regions. For example, in Ukraine and Yemen, ongoing conflicts have severely disrupted healthcare infrastructure (44–46). Such disruption makes the delivery of PrEP or ART services to those in need extremely difficult. In these settings, expanding PrEP coverage or ART access requires more than just medical supplies. It also involves rebuilding healthcare facilities, training local healthcare workers, and ensuring the safe transportation of medications. Furthermore, flexible approaches such as mobile clinics and community-based distribution systems are essential for expanding PrEP coverage in these dynamic environments. Additionally, resource allocation represents a persistent challenge. While international guidelines may recommend certain high-impact interventions, local governments often lack the financial resources to implement them. This situation necessitates innovative financing mechanisms and partnerships to mobilize the required resources (3). Between 1990 and 2021, Eastern Sub-Saharan Africa experienced the most substantial reduction in incidence. This decline signifies that the notable successes of this region in decreasing HIV/AIDS incidence stem from sustained advancement. This discovery aligns with the substantial advancements achieved in the accessibility of ART, PrEP, and voluntary medical male circumcision (47–49). Nevertheless, the region remains beset by discriminatory conditions and domestic laws that not only sustain the epidemic but also exacerbate its persistence, leading to elevated prevalence rates.

Among the 204 countries or territories, Pakistan has experienced the most significant rise in HIV/AIDS incidence. The literacy rate in Pakistan is notably low, particularly among females, resulting in inadequate awareness and understanding of HIV/AIDS and its prevention (50). In WCBA, the likelihood of HIV transmission is markedly heightened by practices such as extramarital sexual relations, unsafe blood transfusions, and the utilization of contaminated syringes. Furthermore, the widespread discrimination and stigma faced by individuals with HIV/AIDS in Pakistani society has resulted in a considerable number of infected individuals choosing to hide their status (51). This has impeded efforts for early detection and treatment and may also heighten the transmission risk within the community. The government of Pakistan must implement measures to address these issues, including the provision of sex education for WCBA and the empowerment of women, to mitigate the burden of HIV/AIDS among WCBA (52). Regarding age-specific patterns, our analysis revealed that women aged 35–39 years bore the highest prevalence of HIV/AIDS, while those aged 30–34 years experienced the peak mortality and DALY burden. The natural history of HIV involves a prolonged clinical latency period. Women infected at younger ages typically progress to advanced stages after 5–10 years (53), leading to concentrated diagnosis and documented morbidity in the 35–39 age group. Furthermore, cumulative exposure risk escalates with age. Women over 30 face amplified infection risks due to extended periods of sexual activity, partner turnover, and vertical transmission exposure during pregnancy (54, 55). Critically, the peak in mortality and DALYs at 30 to 34 years is indicative of significant structural barriers in high-burden settings. Younger women often experience delayed diagnosis due to the stigma associated with HIV and the fragmentation of health services. Even after diagnosis, timely access to ART is frequently impeded by financial limitations, medication shortages, and concerns regarding side effects (3, 5). As a result, untreated infections tend to progress to severe and often fatal complications by their early thirties. We have emphasized the importance of public health interventions, such as improving the accessibility of HIV testing services, reducing the stigma associated with HIV infection, and enhancing the affordability of ART, to reduce the disease burden of HIV/AIDS among WCBA.

Joinpoint regression analysis indicated a significant decline in incidence from 1997 to 2005, coinciding with the introduction of ART in 1996 (56). A subsequent decrease in incidence was noted from 2015 to 2021. The decrease can be ascribed to the implementation of the UNAIDS fast-track strategy in 2016, which set mid-term 90–90-90 testing and treatment objectives for 2020 (33). These objectives are crucial for accomplishing the global aim of eradicating AIDS by 2030 (32). Decomposition analyses reveal that population and epidemiological changes significantly impact the burden of DALYs related to HIV/AIDS among WCBA, both globally and across SDI regions. This indicates that global population growth is directly contributing to the rise in the number of WCBA living with HIV/AIDS. The analysis indicates that epidemiological shifts, especially changes in HIV/AIDS transmission dynamics and population susceptibility, significantly contribute to the increase of DALYs. This highlights the necessity of ongoing monitoring and the modification of prevention and control strategies to tackle the global issue of HIV/AIDS. Predictive analyses indicate a slight decrease in ASPR, alongside more significant reductions in ASIR and ASMR. The significant reductions in ASIR and ASMR reflect considerable global progress in the prevention, management, and treatment of HIV/AIDS. The slight decrease in ASPR indicates the difficulties related to long-term disease management. This highlights the necessity of ongoing monitoring, enhancing prevention and control measures, and fortifying international collaboration.

In the last 30 years, the advancement of ART has significantly influenced the natural progression of HIV infection. The emergence of early diagnosis and the standardization of combination antiretroviral therapy (cART) have initiated a fundamental change in the management of HIV infection. During the 1980s, HIV infection was marked by a significant mortality rate, with an average life expectancy of merely 1–2 years post-diagnosis. The introduction of cART has converted HIV infection into a manageable chronic condition. Clinical studies indicate that 20-year-old female patients commencing ART can anticipate an increase in life expectancy of approximately 9 years (57). The global HIV epidemic persists as a serious concern, with the 2023 UNAIDS report revealing around 1.3 million new infections in 2022, and young women in Sub-Saharan Africa aged 15–24 being infected at three times the rate of their male counterparts in the same age group. This concerning epidemiological profile highlights the urgent necessity for the execution of effective preventive measures for at-risk populations, especially WCBA (4).

In the domain of HIV prevention strategies, HIV testing has become a significant secondary prevention measure, enhancing traditional behavioral interventions and condom use. The prompt diagnosis and treatment of bacterial sexually transmitted infections (STIs) have proven to significantly aid in the prevention of HIV (58). Over the past 15 years, there has been considerable advancement in biomedical prevention strategies, including the creation of PrEP and post-exposure prophylaxis (PEP) (59, 60). The utilization of PrEP has been evidenced to diminish the likelihood of HIV infection by as much as 92 percent, and long-acting injectable PrEP (e.g., cabotegravir), upon its approval in the United States in 2021, has exhibited superior results compared to oral PrEP (61). African nations have made substantial advancements in combating AIDS, evidenced by a 56 percent reduction in infections in Sub-Saharan Africa since 2010, according to the latest UNAIDS 2023 report (4). A study reveals that Sub-Saharan Africa is adopting universal testing and treatment (UTT) to diminish HIV transmission, aspiring to achieve the 90–90-90 targets set by UNAIDS (62, 63). Due to the considerable regional variability in HIV epidemiological profiles, there is an urgent necessity to enact differentiated policy reforms. HIV prevention and control strategies for WCBA should encompass multifaceted interventions, including the optimization of policy frameworks, enhancement of socio-economic empowerment, promotion of participatory community governance, and expansion of community-based rapid testing networks (64).

The global HIV prevention and control framework presently utilizes a multifaceted array of strategies, encompassing MTCT, voluntary male circumcision (VMMC), barrier methods, varied PrEP options (including oral and long-acting injectable forms), and behavioral intervention programs (65–67). The expansion and enhancement of these interventions for the key population of WCBA is a primary emphasis of national AIDS programs. The continuous enhancement of prevention and control strategies necessitates augmented financial investment and a methodical implementation approach that targets constraints such as socio-cultural factors, cognitive barriers, stigma and discrimination, and inequitable access to services. It is essential to guarantee that all individuals can accurately evaluate their infection risk and have access to testing services, both institutional and self-administered, that are devoid of financial burdens and social bias. The current treatment and prevention framework comprises three pillars (treatment, prevention, and diagnosis), yet progress toward the global objective of eradicating the AIDS epidemic can be expedited through the tailored enhancement of service models, precision, and scalability.

Nonetheless, it is crucial to acknowledge that the study possesses certain limitations. First, our analysis relied on model-derived estimates obtained from the GBD 2021 dataset rather than actual surveillance data, and there may be inherent bias between them that could affect the accuracy of the results. Secondly, as an ecological analysis, our study identifies population-level trends but cannot elucidate individual risk factors including sexual behaviors, stigma-related care barriers, and socioeconomic status. Future research linking macro-level burden with micro-level survey data is critical to inform targeted interventions. Thirdly, the lack of data from certain low- and middle-income countries may undermine the accuracy of the estimates. In low- and middle-income countries, the limited accessibility to healthcare services can hinder the ability to conduct comprehensive HIV screening and diagnosis, leading to incomplete case reporting, particularly among economically and socially disadvantaged groups. This situation may have been exacerbated by the COVID-19 pandemic (68), as health systems in these areas often have limited adaptive capacity to handle additional stress. Fourthly, the actual definition of WCBA (15–49 years) is globally inconsistent, potentially underestimating the disease burden in this group. Additionally, the utilization of static SDI categories also undermines the ability of countries to grasp how their socioeconomic evolves over time. Finally, wide UIs in settings like Equatorial Guinea reflect both data scarcity and epidemiological volatility. This suggests that we should interpret rankings from such countries with caution, given the limited surveillance infrastructure and data volatility.

## 5 Conclusion

HIV/AIDS among WCBA constitutes a substantial global health burden. Low SDI region, particularly in Sub-Saharan Africa, account for a disproportionate share of the global HIV/AIDS burden in WCBA, despite recent declines in incidence rates. Conversely, Eastern Europe has experienced a marked upward trend in incidence. Furthermore, the disease burden exhibits different distribution across age groups. Governments urgently need to implement context-specific interventions to address these disparities and further reduce the global burden of HIV/AIDS among WCBA across all regions.

## Data Availability

Publicly available datasets were analyzed in this study. This data can be found at: the data is sourced from a public database, accessible via the following link: https://vizhub.healthdata.org/gbd-results/.

## Footnotes

^1^ http://ghdx.healthdata.org/gbd-results-tool
