# Supplementary material for: Global, regional, and national burden and trends of HIV/AIDS among women of childbearing age from 1990 to 2021: results from Global Burden of Disease 2021
Source: Front Med (Lausanne). 2025 Jul 4;12:1605092. doi: 10.3389/fmed.2025.1605092 (PMC12271156; doi:10.3389/fmed.2025.1605092)

**Global, regional, and national burden and trends of HIV/AIDS among women of childbearing age from 1990 to 2021: results from global burden of disease 2021**

**Supplementary materials**

| Items | Page | Content |
| --- | --- | --- |
| Data | 2 | Details of data and methods |
| Table S1 | 8 | The incidence of HIV/AIDS cases and rates among women of childbearing age across 204 countries in 1990 and 2021, and the trends from 1990 to 2021 |
| Table S2 | 18 | The prevalence of HIV/AIDS cases and rates among women of childbearing age across 204 countries in 1990 and 2021, and the trends from 1990 to 2021 |
| Table S3 | 30 | The mortality of HIV/AIDS cases and rates among women of childbearing age across 204 countries in 1990 and 2021, and the trends from 1990 to 2021 |
| Table S4 | 42 | The DALYs of HIV/AIDS cases and rates among women of childbearing age across 204 countries in 1990 and 2021, and the trends from 1990 to 2021. |
| Figure S1 | 58 | The numbers and rates of incidence, prevalence, mortality, and DALYs for HIV/AIDS among women of childbearing age by SDI regions in 1990 and 2021 |
| Figure S2 | 59 | The rates of incidence, prevalence, mortality, and DALYs for HIV/AIDS among women of childbearing age in different age sex groups |
| Figure S3 | 60 | Decomposition analysis of incidence, prevalence, mortality, and DALYs change in HIV/AIDS among women of childbearing age from 1990 to 2021 at the global level and by SDI. |
| Figure S4 | 61 | Joinpoint regression analysis of the temporal trends of HIV/AIDS among women of childbearing age from 1990 to 2021. |
| Figure S5 | 62 | Prediction of disease burden for HIV/AIDS among women of childbearing age by 2046. |

**Supplementary Data**

In GBD 2021, the data processing workflow is a crucial step to ensure the accuracy and consistency of global disease burden estimates. This process includes systematic adjustments to epidemiological data to correct for biases arising from differences in data sources, definitions, and measurement methods. These adjustments are implemented using complex statistical models such as MR-BRT and DisMod-MR 2.1, ensuring internal consistency in estimates of incidence, prevalence, years lived with disability (YLDs), years of life lost (YLLs), and disability-adjusted life years (DALYs) across different regions, ages, genders, and years. This process aims to minimize heterogeneity in the study results through standardization and correction steps.

1. Data extraction criteria

Data were retrieved for the following univariates categorizations of stroke: (1) cause: HIV/AIDS defined according to the International Classification of Diseases 9/10 (ICD-9/10) or customized classifications in some countries; (2) location: global, 21 GBD regions, 5 regions classified based on the quartiles of the Socio-Demographic Index (SDI) and 204 countries and territories; (3) age: since the GBD 2021 database provides age intervals in consecutive 5-year intervals from 5-9 to 90-94 years of age, the target population for this study ultimately included people between the ages of 15 and 49, all ages and age-standardized; (4) sex: and female; (5) year: from 1990 to 2021. Input data sources and results are available for download from the Global Health Data Exchange (<https://vizhub.healthdata.org/gbd-results/>).

2. Socio-demographic index (SDI)

SDI was originally constructed for GBD 2015 by using the Human Development Index (HDI) methodology, wherein a 0 to 1 index value was determined for each of the original three covariate inputs (TFR in ages 15 to 49 years, EDU15+, and LDI per capita) by using the observed minima and maxima over the estimation period to set the scales. In response to feedback from collaborators and the evolution of the GBD, we have refined the indicator with each GBD cycle. Beginning in GBD 2017, along with our expanded estimation of age‐specific fertility, we replaced TFR with TFU25 as one of the three component indices. The TFU25 provides a better measure of women’s status in society because it focuses on ages at which childbearing disrupts the pursuit of education and entrance into the workforce. In addition, we observed that in highly developed countries, the TFU25 has tended to decline consistently over time despite rebounds in TFR driven by increasing fertility at older ages. Thus, for each covariate input, an index score of 0 represents the minimum level of each covariate input past which selected health outcomes can get no worse, and an indexscore of 1 represents the maximum level of each covariate input past which selected health outcomes cease to improve. As a composite, a ocation with an SDI of 0 would have a theoretical minimum level of sociodemographic development relevant to these health outcomes, and a location with an SDI of 1 (before multiplying by 100 for reporting) would have a theoretical maximum level of sociodemographic development relevant to these health outcomes.

We computed the index scores underlying SDI as follows:

$$I_{cly}=\max\left( \frac{C_{ly}-C_{low}}{C_{high}-C_{low}}, 0.05 \right)$$

Where: $I_{cly}$ is the index for covariate C, location l and year y and is equal to the difference between the value of this covariate in this location year and the lower limit of the covariate divided by the difference between the upper and lower limits for this covariate. For access to SDI information, please visit the public webpage at http://ghdx.healthdata.org/record/ihme-data/gbd-2019-socio-demographic-index-sdi-1950-2019

3. Data processing and heterogeneity control

In GBD 2021, adjustments were made to epidemiological data known to have biases, such as those using alternative case definitions or measurement methods. These adjustments were made using correction factors estimated by MR-BRT (Meta-regression—Bayesian, regularized, trimmed), a collection of statistical models including linear and nonlinear mixed-effects models. The input data included paired estimates of two case definitions or measurement methods for the same age, sex, region, and year. MR-BRT also controlled for heterogeneity through network meta-regression and performed sex-splitting for inputs not reported by sex, and age-sex splitting for data not reported by either. These processes ensured data standardization and consistency, reducing heterogeneity issues caused by varying data sources and definitions. Additionally, input data spanning more than 25 years were disaggregated into finer age-specific estimates using alternative age patterns estimated from other available data sources.

4. Epidemiological estimation and YLD calculation

The prevalence and incidence estimates for most diseases and injuries were derived using DisMod-MR 2.1 (Disease Modeling Meta-Regression Tool, version 2.1). This Bayesian disease modeling tool generates internally consistent prevalence, incidence, remission, and mortality estimates stratified by sex, region, year, and age group. For areas lacking original epidemiological data, DisMod-MR 2.1 utilizes data from higher hierarchical levels as prior information to estimate parameters for lower levels. For certain causes, the Space-Time Gaussian Process Regression (ST-GPR) model was used as an alternative estimation method. For nonfatal causes, prevalence and incidence were further divided into specific sequelae estimates based on their severity. Sequela categories for nonfatal causes can range from asymptomatic to severe, depending on the disease. For most nonfatal causes, the proportion of cases in each sequela category was calculated using Medical Expenditure Panel Survey (MEPS) analysis. Crude YLD rates were estimated by multiplying the sequela-specific prevalence by the corresponding disability weights.

5. Comorbidity adjustment

GBD 2021 adjusted YLDs for comorbidities to account for the coexistence of nonfatal causes in the population, allowing YLDs to be additive within the GBD 2021 cause hierarchy. The coexistence of comorbidities was estimated through simulations involving 20,000 hypothetical individuals for each age, sex, region, and year. Each simulated individual was assigned the probability of having each sequela based on its prevalence. Subsequently, a cumulative disability weight was assigned to each individual by multiplying the disability weights of all assigned sequelae, with appropriate adjustments made to the sequela-specific disability weights.

6. YLLs and DALYs estimation process

In GBD 2021, YLLs were calculated by multiplying the estimated number of deaths by the standard life expectancy at the age of death, stratified by age, sex, region, and year. To ensure accurate attribution of causes of death, GBD 2021 employed the principles of the 11th edition of the International Classification of Diseases (ICD-11), assigning each death to the underlying cause that initiated the chain of events leading to death. For deaths recorded with non-specific, unreliable, or intermediate cause codes, reallocation algorithms were applied to reassign these "garbage codes" to the most probable causes of death. These algorithms were derived from published studies, expert consultations, or regression-based adjustments using data from sources reporting multiple causes of death. The cause of death for most diseases and injuries is estimated using the Cause of Death Ensemble model (CODEm). CODEm employs a set of statistical models, systematically testing the predictive validity of different covariate combinations, then combining the results to estimate the number of deaths for specific causes by location, age, sex, and year. For a small number of causes with sparse data or significant changes in reporting practices, GBD 2021 adopted customized modeling strategies, including the use of prevalence, incidence, case fatality data, or data related to sub-causes to infer causes of death. Through this process, GBD 2021 achieved progress in controlling data heterogeneity and reducing uncertainty. To estimate DALYs in GBD 2021, specific cause mortality rates and YLDs were first estimated. DALYs for each year were then calculated by adding YLLs to YLDs. The uncertainty of YLLs was assumed to be independent of the uncertainty of YLDs. By summing the first set of YLLs and YLDs across 500 simulations, and repeating the process for subsequent simulations, the 95% uncertainty interval for DALYs was ultimately calculated. The estimation of DALYs covered every cause, location, age group, sex, and year, providing a comprehensive assessment of the global health burden.

7.Data visualization

| Name | Cite |
| --- | --- |
| bamp | Schmid V, Geressen F, Held L, Rainer E (2022). _ bamp: Bayesian Age-Period-Cohort Modeling and Prediction_. R package version 2.1.3, https://cran.r-project.org/web/packages/bamp/index.html. |
| data.table | Dowle M, Srinivasan A (2023). _data.table: Extension of `data.frame`_. R package version 1.14.8, <https://CRAN.R-project.org/package=data.table>. |
| digest | Lucas DEwcbA, Tuszynski J, Bengtsson H, Urbanek S, Frasca M, Lewis B, Stokely M, Muehleisen H, Murdoch D, Hester J, Wu W, Kou Q, Onkelinx T, Lang M, Simko V, Hornik K, Neal R, Bell K, de Queljoe M, Suruceanu I, Denney B, Schumacher D, Chang. aW (2022). _digest: Create Compact Hash Digests of R Objects_. R package version 0.6.31, <https://CRAN.R-project.org/package=digest>. |
| gglot2 | H. Wickham. ggplot2: Elegant Graphics for Data Analysis. Springer-Verlag New York, 2016. |
| dplyr | Wickham H, François R, Henry L, Müller K, Vaughan D (2023). _dplyr: A Grammar of Data Manipulation_. R package version 1.1.0, <https://CRAN.R-project.org/package=dplyr>. |
| forcats | Wickham H (2023). _forcats: Tools for Working with Categorical Variables (Factors)_. R package version 1.0.0, <https://CRAN.R-project.org/package=forcats>. |
| ggnewscale | Campitelli E (2022). _ggnewscale: Multiple Fill and Colour Scales in 'ggplot2'_. R package version 0.4.8, <https://CRAN.R-project.org/package=ggnewscale>. |
| ggrepel | Slowikowski K (2023). _ggrepel: Automatically Position Non-Overlapping Text Labels with 'ggplot2'_. R package version 0.9.3, <https://CRAN.R-project.org/package=ggrepel>. |
| ggsci | Xiao N (2023). _ggsci: Scientific Journal and Sci-Fi Themed Color Palettes for 'ggplot2'_. R package version 3.0.0, <https://CRAN.R-project.org/package=ggsci>. |
| inla | Krainski E T, Lindgren F, Rue H (2024). _INLAspacetime: Spatial and Spatio-Temporal Models using 'INLA'_. R package version 0.1.10, https://cran.r-project.org/web/packages/INLAspacetime/index.html |
| lubridate | Garrett Grolemund, Hadley Wickham (2011). _ Dates and Times Made Easy with lubridate. Journal of Statistical Software, 40(3), 1-25. URL <https://www.jstatsoft.org/v40/i03/>. |
| nordpred | Moller B, Weedon-Fekjaer H (2019) ._ nordpred: Fit power5 and poisson Age-Period-Cohort models to calculate prediction of cancer incidence and mortality_. R package version 1.1, URL http://www.kreftregisteret.no/software/nordpred/. |
| patchwork | Pedersen T (2022). _patchwork: The Composer of Plots_. R package version 1.1.2, <https://CRAN.R-project.org/package=patchwork>. |
| purrr | Wickham H, Henry L (2023). _purrr: Functional Programming Tools_. R package version 1.0.1, <https://CRAN.R-project.org/package=purrr>. |
| RColorBrewer | Neuwirth E (2022). _RColorBrewer: ColorBrewer Palettes_. R package version 1.1-3, <https://CRAN.R-project.org/package=RColorBrewer>. |
| readr | Wickham H, Hester J, Bryan J (2023). _readr: Read Rectangular Text Data_. R package version 2.1.4, <https://CRAN.R-project.org/package=readr>. |
| readxl | Wickham H, Bryan J (2023). _readxl: Read Excel Files_. R package version 1.4.2, <https://CRAN.R-project.org/package=readxl>. |
| sf | Pebesma, E., 2018. Simple Features for R: Standardized Support for Spatial Vector Data. The R Journal 10 (1), 439-446, <https://doi.org/10.32614/RJ-2018-009> Pebesma, E., & Bivand, R. (2023). Spatial Data Science: With Applications in R (1st ed.). Chapman and Hall/CRC. <https://doi.org/10.1201/9780429459016> |
| snow | Tierney L, Rossini AJ, Li N, Sevcikova H (2021). _snow: Simple Network of Workstations_. R package version 0.4-4, <https://CRAN.R-project.org/package=snow>. |
| snowfall | Knaus J (2022). _snowfall: Easier Cluster Computing (Based on 'snow')_. R package version 1.84-6.2, <https://CRAN.R-project.org/package=snowfall>. |
| stringr | Wickham H (2022). _stringr: Simple, Consistent Wrappers for Common String Operations_. R package version 1.5.0, <https://CRAN.R-project.org/package=stringr>. |
| tibble | Müller K, Wickham H (2022). _tibble: Simple Data Frames_. R package version 3.1.8, <https://CRAN.R-project.org/package=tibble>. |
| tidyr | Wickham H, Vaughan D, Girlich M (2023). _tidyr: Tidy Messy Data_. R package version 1.3.0, <https://CRAN.R-project.org/package=tidyr>. |
| tidyverse | Wickham H, Averick M, Bryan J, Chang W, McGowan LD, François R, Grolemund G, Hayes A, Henry L, Hester J, Kuhn M, Pedersen TL, Miller E, Bache SM, Müller K, Ooms J, Robinson D, Seidel DP, Spinu V, Takahashi K, Vaughan D, Wilke C, Woo K, Yutani H (2019). “Welcome to the tidyverse.” _Journal of Open Source Software_, *4*(43), 1686. doi:10.21105/joss.01686 <https://doi.org/10.21105/joss.01686>. |
| viridis | Simon Garnier, Noam Ross, Robert Rudis, Antônio P. Camargo, Marco Sciaini, and Cédric Scherer (2021). Rvision - Colorblind-Friendly Color Maps for R. R package version 0.6.2 |
| viridisLite | Simon Garnier, Noam Ross, Robert Rudis, Antônio P. Camargo, Marco Sciaini, and Cédric Scherer (2022). Rvision - Colorblind-Friendly Color Maps for R. R package version 0.4.1 |

8 SDI quintiles for countries estimated in GBD 2021

| SDI quintile | Locations included based on SDI values in 2021 from GBD 2021 results |
| --- | --- |
| Low SDI（0.00-0.47） | Afghanistan, Benin, Burkina Faso, Burundi, Central African Republic, Chad, Cote d'lvoire, Democratic Republic of the Congo, Eritrea, Ethiopia, Gambia, Guinea, Guinea-Bissau, Haiti, Liberia, Madagascar, Malawi, Mali, Mozambique, Nepal, Niger, Pakistan, Papua New Guinea, Rwanda, Senegal, Sierra Leone, Solomon Islands, Somalia, South Sudan, Togo, Uganda, United Republic of Tanzania, Yemen |
| Low-middle SDI（0.47-0.62） | Angola, Bangladesh, Belize, Bhutan, Bolivia (Plurinational State of), Cabo Verde, Cambodia, Cameroon, Comoros, Congo, Democratic People's Republic of Korea, Djibouti, Dominican Republic, El Salvador, Eswatini, Ghana, Guatemala, Honduras, India, Kenya, Kiribati, Kyrgyzstan, Lao People's Democratic Republic, Lesotho, Maldives, Marshall Islands, Mauritania, Micronesia (Federated States of), Mongolia, Morocco, Myanmar, Nicaragua, Nigeria, Palestine, Sao Tome and Principe, Sudan, Tajikistan, Timor-Leste, Tuvalu, Vanuatu, Venezuela (Bolivarian Republic of), Zambia, Zimbabwe |
| Middle SDI（0.62-0.71） | Albania, Algeria, Armenia, Azerbaijan, Botswana, Brazil, China, Colombia, Costa Rica, Cuba, Ecuador, Egypt, Equatorial Guinea, Fiji, Gabon, Grenada, Guyana, Indonesia, Iran (Islamic Republic of), Iraq, Jamaica, Mexico, Namibia, Nauru, Panama, Paraguay, Peru, Philippines, Saint Lucia, Saint Vincent and the Grenadines, Samoa, South Africa, Suriname, Syrian Arab Republic, Thailand, Tokelau, Tonga, Tunisia, Turkmenistan, Uzbekistan, Viet Nam |
| High-middle SDI（0.71-0.81） | American Samoa, Antigua and Barbuda, Argentina, Bahamas, Bahrain, Barbados, Belarus, Bosnia and Herzegovina, Bulgaria, Chile, Cook Islands, Croatia, Dominica, Georgia, Greece, Greenland, Hungary, Israel, Italy, Jordan, Kazakhstan, Lebanon, Libya, Malaysia, Malta, Mauritius, Montenegro, Niue, North Macedonia, Northern Mariana Islands, Oman, Palau, Poland, Portugal, Republic of Moldova, Romania, Russian Federation, Saint Kitts and Nevis, Saudi Arabia, Serbia, Seychelles, Spain, Sri Lanka, Trinidad and Tobago, Turkey, Ukraine, United States Virgin Islands, Uruguay |
| High SDI（0.81-1.00） | Andorra, Australia, Austria, Belgium, Bermuda, Brunei Darussalam, Canada, Cyprus, Czechia, Denmark, Estonia, Finland, France, Germany, Guam, Iceland, Ireland, Japan, Kuwait, Latvia, Lithuania, uxembourg, Monaco, Netherlands, New Zealand, Norway, Puerto Rico, Qatar, Republic of Korea, San Marino, Singapore, Slovakia, Slovenia, Sweden, Switzerland, Taiwan (Province of China), United Arab Emirates, United Kingdom, United States of America |

Abbreviation: SDI, socio-demographic index.

**Supplementary Table S1**

The incidence of HIV/AIDS cases and rates among women of childbearing age across 204 countries in 1990 and 2021, and the trends from 1990 to 2021

| location | Num_1990 | ASR_1990 | Num_2021 | ASR_2021 | EAPC_CI |
| --- | --- | --- | --- | --- | --- |
| Afghanistan | 12 (5 to 31) | 0.59 (0.24 to 1.48) | 102 (41 to 188) | 1.42 (0.56 to 2.62) | 3.65 (3.19 to 4.12) |
| Albania | 1 (1 to 2) | 0.14 (0.1 to 0.21) | 1 (0 to 1) | 0.1 (0.05 to 0.19) | -1.8 (-2.15 to -1.45) |
| Algeria | 29 (22 to 39) | 0.49 (0.36 to 0.65) | 696 (512 to 905) | 6.17 (4.54 to 8.04) | 8.87 (8.1 to 9.66) |
| American Samoa | 0 (0 to 0) | 0.63 (0.35 to 1.38) | 2 (1 to 3) | 17.97 (9.79 to 26.65) | 11.01 (10.64 to 11.37) |
| Andorra | 0 (0 to 0) | 0.87 (0.62 to 1.17) | 0 (0 to 0) | 0.92 (0.66 to 1.23) | -0.42 (-0.98 to 0.14) |
| Angola | 1411 (300 to 3465) | 60.81 (12.89 to 148.98) | 32498 (14676 to 62202) | 431.35 (194.52 to 824.85) | 4.5 (3.68 to 5.33) |
| Antigua and Barbuda | 3 (2 to 7) | 18.92 (10.83 to 43.28) | 4 (2 to 6) | 15.03 (8.26 to 26.66) | -0.14 (-0.49 to 0.21) |
| Argentina | 1249 (896 to 1660) | 15.64 (11.23 to 20.79) | 1698 (1233 to 2236) | 14.3 (10.41 to 18.81) | -0.23 (-0.35 to -0.11) |
| Armenia | 3 (0 to 14) | 0.3 (0 to 1.55) | 61 (30 to 114) | 7.44 (3.64 to 13.89) | 14.52 (11.82 to 17.28) |
| Australia | 43 (27 to 62) | 0.97 (0.62 to 1.41) | 173 (72 to 330) | 3 (1.29 to 5.63) | 2.94 (2.41 to 3.46) |
| Austria | 157 (87 to 249) | 7.7 (4.26 to 12.27) | 83 (31 to 165) | 4.23 (1.59 to 8.35) | 0.28 (-0.57 to 1.14) |
| Azerbaijan | 9 (5 to 25) | 0.45 (0.23 to 1.22) | 74 (35 to 147) | 2.55 (1.19 to 5.02) | 5.17 (4.7 to 5.65) |
| Bahamas | 100 (74 to 132) | 132.95 (98.12 to 174.26) | 119 (61 to 207) | 114.57 (58.2 to 199.81) | -0.86 (-1.41 to -0.3) |
| Bahrain | 1 (1 to 2) | 0.95 (0.61 to 1.43) | 3 (2 to 5) | 0.93 (0.51 to 1.6) | -1.49 (-2.12 to -0.85) |
| Bangladesh | 2 (2 to 3) | 0.01 (0.01 to 0.01) | 522 (362 to 725) | 1.12 (0.78 to 1.56) | 15.48 (12.7 to 18.32) |
| Barbados | 41 (27 to 61) | 58.13 (38.31 to 86.78) | 30 (17 to 53) | 46.28 (27.22 to 79.75) | -2.87 (-3.61 to -2.12) |
| Belarus | 187 (0 to 593) | 7.05 (0 to 22.33) | 1233 (645 to 2450) | 54.7 (29.34 to 108.84) | 9.35 (8.42 to 10.28) |
| Belgium | 180 (102 to 284) | 7.26 (4.06 to 11.44) | 255 (116 to 409) | 10.67 (5.02 to 16.99) | 0.1 (-0.32 to 0.54) |
| Belize | 13 (9 to 18) | 32.55 (22.92 to 45.27) | 60 (33 to 106) | 48.13 (26.81 to 83.82) | -0.38 (-1.03 to 0.26) |
| Benin | 964 (477 to 1662) | 86.47 (42.87 to 148.24) | 1793 (719 to 3805) | 56.48 (22.57 to 120.74) | -4.27 (-5.22 to -3.3) |
| Bermuda | 4 (2 to 8) | 20.19 (11.69 to 47.21) | 1 (0 to 1) | 6.81 (3.85 to 11.82) | -3.13 (-3.62 to -2.65) |
| Bhutan | 3 (2 to 5) | 2 (1.09 to 3.32) | 16 (6 to 29) | 7.49 (3.04 to 13.7) | 3.39 (2.28 to 4.5) |
| Bolivia (Plurinational State of) | 41 (10 to 113) | 2.68 (0.62 to 7.33) | 285 (162 to 449) | 9.03 (5.11 to 14.23) | -1.02 (-3.45 to 1.47) |
| Bosnia and Herzegovina | 0 (0 to 1) | 0.04 (0.03 to 0.05) | 1 (0 to 1) | 0.09 (0.04 to 0.15) | 2.18 (1.36 to 3) |
| Botswana | 9214 (6335 to 12784) | 2752.09 (1867.42 to 3855.41) | 3193 (1587 to 5994) | 462.93 (230.05 to 868.93) | -6.98 (-7.51 to -6.44) |
| Brazil | 6775 (5084 to 8934) | 16.66 (12.6 to 21.81) | 16515 (8836 to 28965) | 28.28 (15.05 to 49.56) | 1.9 (1.45 to 2.35) |
| Brunei Darussalam | 1 (1 to 2) | 1.68 (0.87 to 2.79) | 6 (2 to 11) | 4.45 (1.94 to 8.42) | 3.68 (2.91 to 4.45) |
| Bulgaria | 13 (0 to 24) | 0.65 (0 to 1.18) | 32 (15 to 55) | 2.93 (1.45 to 4.87) | 4.15 (3.61 to 4.69) |
| Burkina Faso | 12509 (7056 to 20113) | 587.89 (330.28 to 947.03) | 852 (289 to 1854) | 15.66 (5.27 to 34.1) | -9.63 (-10.15 to -9.1) |
| Burundi | 17487 (5906 to 32983) | 1376.82 (461.03 to 2588.23) | 346 (146 to 704) | 11.34 (4.82 to 23.05) | -13.82 (-14.45 to -13.17) |
| Cabo Verde | 121 (33 to 245) | 149.92 (41.2 to 304.12) | 40 (8 to 180) | 25.89 (5.04 to 117.9) | -5.47 (-5.84 to -5.1) |
| Cambodia | 11 (8 to 15) | 0.44 (0.31 to 0.59) | 497 (274 to 776) | 10.77 (5.93 to 16.86) | -1.96 (-6.64 to 2.96) |
| Cameroon | 9849 (5896 to 14975) | 405.4 (242.16 to 619.2) | 16812 (8792 to 29641) | 214.83 (111.67 to 380.62) | -3.97 (-4.81 to -3.12) |
| Canada | 408 (162 to 741) | 5.55 (2.17 to 10.23) | 851 (320 to 1602) | 10.75 (4.19 to 20.02) | 1.73 (1.32 to 2.14) |
| Central African Republic | 20034 (11473 to 32727) | 3096.93 (1780.53 to 5033.94) | 3567 (1108 to 8917) | 264.17 (82.39 to 663.41) | -6.18 (-6.91 to -5.44) |
| Chad | 3197 (1227 to 6169) | 237.75 (91.41 to 458.3) | 2904 (1101 to 6063) | 76.82 (29.13 to 160.13) | -4.01 (-4.32 to -3.7) |
| Chile | 112 (64 to 172) | 2.98 (1.72 to 4.56) | 675 (303 to 1255) | 14.15 (6.41 to 26.27) | 4.45 (4 to 4.91) |
| China | 1838 (1103 to 3154) | 0.57 (0.34 to 1) | 4246 (1963 to 7464) | 1.52 (0.7 to 2.71) | 1 (-0.4 to 2.42) |
| Colombia | 389 (146 to 1579) | 4.39 (1.67 to 17.58) | 1806 (878 to 3333) | 13.59 (6.61 to 25.08) | 3.4 (2.8 to 4.02) |
| Comoros | 0 (0 to 1) | 0.25 (0.06 to 0.64) | 1 (0 to 1) | 0.26 (0.09 to 0.47) | -0.57 (-2.73 to 1.63) |
| Congo | 6725 (3194 to 11794) | 1182.24 (564.4 to 2061.58) | 3643 (1134 to 9167) | 256.58 (79.95 to 643.92) | -3.7 (-4.33 to -3.07) |
| Cook Islands | 0 (0 to 0) | 0.65 (0.35 to 1.41) | 1 (0 to 1) | 19.14 (10.46 to 28.48) | 11.19 (10.89 to 11.49) |
| Costa Rica | 22 (14 to 34) | 2.82 (1.82 to 4.28) | 81 (40 to 139) | 6.77 (3.34 to 11.57) | 1.88 (1.4 to 2.35) |
| Coted'Ivoire | 50085 (29408 to 76743) | 1804.78 (1056.62 to 2777.34) | 7069 (3227 to 13828) | 106.61 (48.58 to 209.26) | -7.81 (-8.28 to -7.34) |
| Croatia | 8 (5 to 13) | 0.68 (0.41 to 1.04) | 6 (2 to 10) | 0.65 (0.27 to 1.16) | -0.47 (-1.15 to 0.22) |
| Cuba | 49 (34 to 72) | 1.52 (1.04 to 2.21) | 273 (106 to 669) | 10.79 (4.27 to 26.38) | 9.85 (8.63 to 11.07) |
| Cyprus | 1 (0 to 1) | 0.31 (0.15 to 0.53) | 4 (2 to 7) | 1.62 (0.89 to 2.91) | 5.34 (4.55 to 6.14) |
| Czechia | 4 (3 to 7) | 0.17 (0.11 to 0.28) | 17 (6 to 31) | 0.98 (0.37 to 1.74) | 4.98 (4.12 to 5.85) |
| Democratic People's Republic of Korea | 9 (3 to 18) | 0.15 (0.04 to 0.31) | 342 (62 to 785) | 5.23 (0.95 to 11.98) | 8.41 (5.97 to 10.91) |
| Democratic Republic of the Congo | 39663 (23583 to 61981) | 460.59 (274.44 to 720.33) | 3334 (1686 to 6053) | 15.98 (8.08 to 29.04) | -9.53 (-10.01 to -9.04) |
| Denmark | 78 (56 to 103) | 6 (4.32 to 7.97) | 32 (23 to 43) | 2.58 (1.86 to 3.42) | -2.59 (-3.01 to -2.17) |
| Djibouti | 23 (3 to 80) | 23.72 (2.83 to 81.77) | 470 (144 to 1166) | 144.93 (44.41 to 360.24) | -1.26 (-3.86 to 1.41) |
| Dominica | 3 (2 to 3) | 14.61 (10.28 to 20.05) | 2 (1 to 3) | 11.38 (5.82 to 21.29) | -0.89 (-1.7 to -0.07) |
| Dominican Republic | 3498 (1496 to 6390) | 181.51 (77.01 to 333.07) | 662 (165 to 1782) | 22.7 (5.66 to 61.13) | -5.51 (-6.11 to -4.91) |
| Ecuador | 64 (34 to 162) | 2.35 (1.27 to 5.92) | 953 (456 to 1855) | 19.69 (9.46 to 38.19) | 7.23 (6.39 to 8.08) |
| Egypt | 32 (19 to 51) | 0.24 (0.15 to 0.38) | 304 (136 to 581) | 1.13 (0.51 to 2.17) | 6.17 (5.66 to 6.69) |
| El Salvador | 92 (54 to 143) | 6.79 (4.07 to 10.47) | 196 (100 to 360) | 10.77 (5.49 to 19.83) | 0.2 (-1.04 to 1.46) |
| Equatorial Guinea | 165 (49 to 406) | 166.79 (49.6 to 409.94) | 4443 (889 to 11928) | 1217.3 (244.93 to 3255.72) | 4.93 (3.98 to 5.9) |
| Eritrea | 3055 (862 to 5837) | 387.45 (109.26 to 740.87) | 361 (155 to 730) | 22.05 (9.5 to 44.57) | -10.1 (-10.65 to -9.54) |
| Estonia | 3 (1 to 5) | 0.68 (0.33 to 1.33) | 47 (27 to 83) | 20.68 (12.23 to 36.33) | 8 (3.71 to 12.47) |
| Eswatini | 309 (5 to 1627) | 147.47 (2.54 to 773.41) | 2211 (1053 to 3897) | 659.61 (312.4 to 1169.18) | -0.86 (-3.76 to 2.14) |
| Ethiopia | 41441 (16921 to 73328) | 361.82 (147.22 to 641.5) | 12664 (7093 to 25941) | 46.82 (26.3 to 95.08) | -7.56 (-7.97 to -7.16) |
| Fiji | 5 (3 to 11) | 2.32 (1.28 to 5.65) | 23 (12 to 43) | 10.18 (5.11 to 18.69) | 3.33 (2.56 to 4.1) |
| Finland | 30 (16 to 48) | 2.33 (1.25 to 3.71) | 18 (7 to 36) | 1.62 (0.64 to 3.1) | 0.38 (-0.36 to 1.12) |
| France | 970 (656 to 1402) | 6.66 (4.51 to 9.63) | 1475 (1041 to 1980) | 10.72 (7.62 to 14.32) | 1.49 (0.8 to 2.19) |
| Gabon | 972 (453 to 1687) | 434.31 (203.45 to 753.84) | 1305 (445 to 3334) | 267.67 (91.32 to 684.75) | -2.2 (-2.79 to -1.6) |
| Gambia | 155 (52 to 328) | 67.46 (22.92 to 142.39) | 1544 (611 to 3074) | 256.84 (101.73 to 510.21) | 1.84 (0.71 to 2.98) |
| Georgia | 24 (5 to 72) | 1.72 (0.38 to 5.07) | 52 (26 to 111) | 6.04 (3.05 to 12.8) | 7.59 (5.33 to 9.9) |
| Germany | 702 (421 to 1090) | 3.6 (2.15 to 5.6) | 507 (211 to 1025) | 2.99 (1.27 to 5.98) | 2.12 (1.22 to 3.02) |
| Ghana | 11341 (7630 to 15757) | 317.33 (213.81 to 440.48) | 14521 (7815 to 24842) | 158.3 (85.07 to 270.89) | -3.55 (-3.96 to -3.14) |
| Greece | 51 (29 to 78) | 2.09 (1.17 to 3.17) | 83 (43 to 135) | 4.59 (2.45 to 7.3) | 2.71 (2.12 to 3.31) |
| Greenland | 2 (1 to 3) | 14.36 (8.46 to 21.46) | 2 (0 to 5) | 15.95 (2.32 to 37.15) | 1.2 (0.65 to 1.75) |
| Grenada | 3 (2 to 6) | 13.22 (7.86 to 29.32) | 1 (1 to 2) | 3.9 (2.25 to 6.73) | -4.31 (-4.5 to -4.12) |
| Guam | 0 (0 to 1) | 0.75 (0.4 to 1.86) | 2 (1 to 4) | 5.95 (2.99 to 11.15) | 4.74 (3.88 to 5.6) |
| Guatemala | 174 (118 to 257) | 9.5 (6.5 to 13.86) | 317 (165 to 591) | 6.7 (3.51 to 12.49) | -2.64 (-3.36 to -1.91) |
| Guinea | 1862 (822 to 3440) | 134.1 (59.3 to 247.56) | 3704 (1643 to 6850) | 111.48 (49.54 to 206.58) | -2.07 (-2.77 to -1.38) |
| Guinea-Bissau | 252 (49 to 520) | 107.51 (21.19 to 221.94) | 2131 (163 to 6039) | 406.88 (31.29 to 1149.83) | 2.79 (2.02 to 3.57) |
| Guyana | 95 (63 to 141) | 44.21 (29.72 to 64.93) | 124 (69 to 215) | 58.21 (32.7 to 101.39) | -0.98 (-2.06 to 0.12) |
| Haiti | 10339 (5221 to 17345) | 655.72 (330.56 to 1103.05) | 5567 (1832 to 11820) | 156.47 (51.38 to 332.99) | -3.74 (-4.21 to -3.27) |
| Honduras | 82 (59 to 111) | 7.43 (5.4 to 9.95) | 77 (45 to 131) | 2.5 (1.46 to 4.25) | -3.56 (-4.5 to -2.61) |
| Hungary | 13 (8 to 18) | 0.49 (0.33 to 0.68) | 20 (8 to 38) | 0.85 (0.36 to 1.52) | 1.14 (0.21 to 2.08) |
| Iceland | 2 (1 to 3) | 2.83 (1.63 to 4.3) | 6 (2 to 12) | 7.8 (3.06 to 14.83) | 5.07 (3.95 to 6.2) |
| India | 5836 (3233 to 9933) | 2.85 (1.57 to 4.86) | 24737 (15846 to 36752) | 6.48 (4.14 to 9.64) | -2.43 (-4.62 to -0.2) |
| Indonesia | 268 (86 to 425) | 0.55 (0.18 to 0.87) | 3032 (1850 to 4888) | 4.19 (2.55 to 6.77) | 8.42 (6.89 to 9.96) |
| Iran (Islamic Republic of) | 14 (6 to 43) | 0.11 (0.04 to 0.35) | 613 (339 to 993) | 2.65 (1.48 to 4.27) | 11.32 (10.08 to 12.58) |
| Iraq | 12 (5 to 29) | 0.27 (0.12 to 0.66) | 140 (34 to 413) | 1.28 (0.3 to 3.86) | 4.71 (4.08 to 5.34) |
| Ireland | 74 (42 to 119) | 8.49 (4.78 to 13.59) | 74 (26 to 145) | 7.15 (2.62 to 13.96) | -1.1 (-1.69 to -0.5) |
| Israel | 44 (22 to 76) | 3.6 (1.81 to 6.26) | 137 (63 to 227) | 6.42 (2.98 to 10.62) | 2.33 (1.95 to 2.71) |
| Italy | 1670 (1003 to 2725) | 11.74 (7.06 to 19.15) | 626 (338 to 1053) | 5.4 (2.99 to 8.93) | -0.99 (-1.49 to -0.48) |
| Jamaica | 240 (118 to 572) | 39.91 (19.87 to 94.93) | 343 (167 to 673) | 43.52 (21.14 to 84.71) | 0.47 (0.21 to 0.73) |
| Japan | 115 (54 to 203) | 0.36 (0.17 to 0.64) | 235 (118 to 371) | 1.13 (0.6 to 1.76) | 3.95 (3.53 to 4.38) |
| Jordan | 2 (1 to 3) | 0.22 (0.14 to 0.35) | 6 (3 to 9) | 0.17 (0.1 to 0.29) | -1.75 (-2.29 to -1.2) |
| Kazakhstan | 313 (200 to 458) | 7.17 (4.55 to 10.54) | 2358 (1143 to 4733) | 46.46 (22.62 to 93.39) | 7.16 (6.7 to 7.62) |
| Kenya | 122150 (97652 to 147911) | 2360.47 (1892.11 to 2855.88) | 18240 (12696 to 24620) | 137.76 (96.05 to 185.57) | -8.8 (-9.41 to -8.19) |
| Kiribati | 0 (0 to 1) | 1.9 (1.33 to 2.62) | 0 (0 to 1) | 1.01 (0.35 to 2.81) | -1.03 (-1.67 to -0.39) |
| Kuwait | 3 (2 to 4) | 0.7 (0.51 to 0.93) | 1 (1 to 2) | 0.08 (0.04 to 0.15) | -8.07 (-9.87 to -6.24) |
| Kyrgyzstan | 16 (10 to 23) | 1.47 (0.96 to 2.17) | 967 (446 to 1809) | 54.91 (25.15 to 102.48) | 12.48 (11.54 to 13.44) |
| Lao People's Democratic Republic | 1 (0 to 1) | 0.07 (0.04 to 0.13) | 124 (80 to 182) | 6.1 (3.91 to 9.01) | 9.79 (5.03 to 14.76) |
| Latvia | 61 (38 to 94) | 9.17 (5.6 to 14.25) | 203 (94 to 315) | 49.34 (24.61 to 75.46) | 4.75 (4.13 to 5.36) |
| Lebanon | 5 (4 to 7) | 0.68 (0.48 to 0.93) | 51 (37 to 68) | 3.39 (2.43 to 4.51) | 4.91 (4.44 to 5.38) |
| Lesotho | 4867 (3182 to 6929) | 1279.65 (835.37 to 1824.64) | 6646 (3727 to 10703) | 1257.45 (704.17 to 2026.53) | -2.19 (-3.11 to -1.26) |
| Liberia | 878 (242 to 2024) | 153.61 (42.44 to 353.64) | 1078 (454 to 2258) | 78.19 (32.93 to 163.93) | -3.54 (-4.26 to -2.82) |
| Libya | 8 (1 to 40) | 0.93 (0.07 to 4.41) | 59 (1 to 377) | 2.97 (0.04 to 18.96) | 3.29 (2.42 to 4.17) |
| Lithuania | 44 (30 to 62) | 4.71 (3.2 to 6.57) | 54 (32 to 89) | 8.39 (5.01 to 13.97) | 1.1 (0.78 to 1.42) |
| Luxembourg | 12 (7 to 19) | 12.63 (7.06 to 19.77) | 13 (5 to 24) | 8.28 (3.51 to 15.97) | -0.53 (-1.2 to 0.14) |
| Madagascar | 29 (7 to 66) | 1.06 (0.28 to 2.42) | 2901 (1021 to 6266) | 41 (14.48 to 88.44) | 7.05 (4.39 to 9.78) |
| Malawi | 53445 (38311 to 70814) | 2327.43 (1665.46 to 3101.98) | 15794 (9070 to 25450) | 318.42 (182.59 to 515.45) | -5.66 (-6 to -5.31) |
| Malaysia | 305 (167 to 517) | 7.09 (3.89 to 12) | 1113 (546 to 2095) | 12.9 (6.31 to 24.32) | 0.97 (0.48 to 1.47) |
| Maldives | 0 (0 to 0) | 0.61 (0.38 to 0.93) | 1 (1 to 1) | 0.73 (0.44 to 1.19) | -0.74 (-1.27 to -0.2) |
| Mali | 3100 (915 to 6705) | 159.34 (47.02 to 343.93) | 2840 (1183 to 5713) | 53.19 (22.19 to 107.11) | -5.06 (-5.62 to -4.49) |
| Malta | 3 (2 to 4) | 2.85 (1.71 to 4.28) | 6 (2 to 11) | 6.23 (2.4 to 11.8) | 3.31 (2.67 to 3.95) |
| Marshall Islands | 0 (0 to 0) | 1.43 (0.81 to 3.08) | 6 (3 to 8) | 37.78 (21.14 to 55.44) | 10.53 (10.21 to 10.84) |
| Mauritania | 1 (0 to 2) | 0.15 (0.06 to 0.36) | 0 (0 to 0) | 0.02 (0.01 to 0.04) | -7.6 (-9.49 to -5.67) |
| Mauritius | 7 (5 to 11) | 2.41 (1.74 to 3.51) | 58 (29 to 110) | 18.43 (9.39 to 34.2) | 6.92 (4.14 to 9.78) |
| Mexico | 2221 (1550 to 3271) | 10.2 (7.29 to 14.83) | 2810 (1686 to 4471) | 8.11 (4.87 to 12.86) | 0.91 (0.27 to 1.55) |
| Micronesia (Federated States of) | 0 (0 to 1) | 1.46 (0.82 to 3.14) | 10 (6 to 15) | 39.12 (21.85 to 57.27) | 10.68 (10.39 to 10.98) |
| Monaco | 0 (0 to 0) | 1.36 (0.95 to 1.89) | 0 (0 to 0) | 1.71 (1.25 to 2.24) | 0.64 (0.33 to 0.94) |
| Mongolia | 0 (0 to 0) | 0.01 (0 to 0) | 4 (1 to 16) | 0.5 (0.1 to 1.8) | 6.93 (4.08 to 9.87) |
| Montenegro | 1 (0 to 1) | 0.41 (0.28 to 0.57) | 1 (1 to 2) | 0.94 (0.54 to 1.52) | 1.7 (1.13 to 2.28) |
| Morocco | 190 (114 to 300) | 2.96 (1.77 to 4.7) | 259 (164 to 372) | 2.68 (1.7 to 3.85) | -0.09 (-1.17 to 1) |
| Mozambique | 13582 (6557 to 24454) | 414.2 (201.67 to 745.36) | 61214 (22147 to 132180) | 773.93 (279.3 to 1668.43) | 0.9 (-0.09 to 1.9) |
| Myanmar | 2295 (1259 to 3732) | 21.5 (11.73 to 35.22) | 4079 (2093 to 6191) | 27.07 (13.9 to 41.06) | -1.18 (-2.34 to 0) |
| Namibia | 2524 (1675 to 3637) | 752.49 (498.99 to 1085.57) | 2362 (1454 to 3535) | 353.95 (217.19 to 530.6) | -4.5 (-5.63 to -3.36) |
| Nauru | 0 (0 to 0) | 0.64 (0.34 to 1.41) | 1 (0 to 1) | 19.03 (10.4 to 28.38) | 11.02 (10.7 to 11.35) |
| Nepal | 0 to 0 | 0 (0 to 0) | 268 (170 to 399) | 2.9 (1.82 to 4.33) | 5.91 (0.67 to 11.43) |
| Netherlands | 80 (45 to 124) | 2.02 (1.15 to 3.13) | 95 (38 to 183) | 2.69 (1.09 to 5.17) | 1.32 (0.16 to 2.49) |
| New Zealand | 10 (5 to 15) | 1.06 (0.6 to 1.66) | 15 (7 to 30) | 1.36 (0.63 to 2.66) | 2.11 (1.33 to 2.89) |
| Nicaragua | 13 (8 to 20) | 1.28 (0.81 to 2.01) | 252 (106 to 524) | 13.66 (5.73 to 28.5) | 7.32 (6.72 to 7.92) |
| Niger | 1512 (528 to 3028) | 86.81 (30.49 to 173.25) | 413 (122 to 1099) | 8.14 (2.42 to 21.61) | -8.84 (-9.49 to -8.18) |
| Nigeria | 42035 (30796 to 55041) | 208.29 (152.55 to 273.42) | 69366 (52599 to 92110) | 125.49 (95.26 to 166.15) | -2.86 (-3.24 to -2.47) |
| Niue | 0 (0 to 0) | 0.63 (0.33 to 1.42) | 0 (0 to 0) | 18.41 (9.93 to 27.63) | 10.98 (10.65 to 11.32) |
| North Macedonia | 0 (0 to 0) | 0.04 (0.03 to 0.05) | 0 (0 to 1) | 0.09 (0.05 to 0.17) | 1.84 (1.28 to 2.41) |
| Northern Mariana Islands | 0 (0 to 1) | 1.55 (0.83 to 3.9) | 1 (0 to 1) | 6.47 (3.27 to 12.04) | 2.86 (1.94 to 3.79) |
| Norway | 70 (46 to 102) | 6.67 (4.37 to 9.76) | 93 (40 to 173) | 8.12 (3.52 to 15.06) | 0.58 (0.13 to 1.04) |
| Oman | 3 (2 to 5) | 0.86 (0.54 to 1.34) | 37 (13 to 63) | 3.76 (1.37 to 6.47) | 3.81 (2.76 to 4.87) |
| Pakistan | 1 (0 to 5) | 0 (0 to 0.02) | 7498 (115 to 39075) | 12.08 (0.18 to 63.44) | 32.38 (27.89 to 37.03) |
| Palau | 0 (0 to 0) | 0.64 (0.35 to 1.39) | 1 (0 to 1) | 17.05 (9.14 to 25.88) | 10.87 (10.63 to 11.11) |
| Palestine | 0 (0 to 1) | 0.07 (0.03 to 0.14) | 13 (6 to 26) | 0.97 (0.42 to 1.98) | 9.06 (7.24 to 10.92) |
| Panama | 166 (98 to 262) | 26.27 (15.7 to 40.91) | 446 (242 to 786) | 41 (22.29 to 72.39) | 1.11 (0.39 to 1.84) |
| Papua New Guinea | 21 (8 to 41) | 2.13 (0.85 to 4.24) | 1778 (761 to 3212) | 67.84 (28.98 to 122.82) | 6.29 (3.3 to 9.37) |
| Paraguay | 28 (6 to 99) | 2.91 (0.66 to 10.47) | 240 (111 to 475) | 12.45 (5.72 to 24.63) | 4.15 (3.03 to 5.28) |
| Peru | 398 (186 to 1271) | 6.91 (3.24 to 22.33) | 811 (376 to 1531) | 8.36 (3.87 to 15.79) | 0.67 (0.33 to 1.02) |
| Philippines | 17 (8 to 34) | 0.11 (0.05 to 0.22) | 7264 (3467 to 13310) | 23.81 (11.43 to 43.58) | 9.18 (6.77 to 11.64) |
| Poland | 51 (23 to 95) | 0.56 (0.25 to 1.05) | 131 (53 to 265) | 1.57 (0.66 to 3.09) | 1.37 (0.61 to 2.13) |
| Portugal | 574 (410 to 760) | 22.9 (16.39 to 30.27) | 104 (72 to 143) | 4.73 (3.35 to 6.41) | -6.74 (-7.33 to -6.15) |
| Puerto Rico | 1250 (972 to 1579) | 131.41 (102.32 to 165.91) | 102 (57 to 177) | 13.84 (7.79 to 23.84) | -6.79 (-8 to -5.56) |
| Qatar | 1 (1 to 2) | 1.6 (1.07 to 2.33) | 1 (1 to 2) | 0.27 (0.15 to 0.44) | -7.11 (-8.22 to -5.99) |
| Republic of Korea | 36 (0 to 87) | 0.28 (0 to 0.7) | 76 (16 to 164) | 0.81 (0.21 to 1.65) | 3.51 (2.63 to 4.39) |
| Republic of Moldova | 61 (21 to 283) | 5.24 (1.74 to 24.38) | 220 (106 to 419) | 24.41 (11.95 to 45.83) | 4.16 (2.28 to 6.08) |
| Romania | 84 (51 to 129) | 1.5 (0.91 to 2.3) | 204 (98 to 383) | 6.4 (3.06 to 11.95) | 2.38 (1.01 to 3.76) |
| Russian Federation | 897 (550 to 1296) | 2.42 (1.47 to 3.54) | 26603 (16619 to 44321) | 82.92 (52.82 to 138.02) | 14.32 (12.87 to 15.79) |
| Rwanda | 10990 (3496 to 22031) | 674.59 (216.43 to 1349.67) | 3939 (1820 to 7646) | 113.74 (52.48 to 221.29) | -6.16 (-6.59 to -5.73) |
| Saint Kitts and Nevis | 3 (1 to 6) | 24.67 (13.53 to 58.12) | 4 (3 to 6) | 28.86 (19.48 to 41) | 0.39 (-0.22 to 1) |
| Saint Lucia | 31 (21 to 43) | 91.9 (64.74 to 126.41) | 19 (10 to 35) | 44.85 (22.82 to 80.65) | -2.94 (-3.8 to -2.06) |
| Saint Vincent and the Grenadines | 13 (9 to 20) | 49.97 (33.05 to 72.49) | 6 (3 to 12) | 24 (12.74 to 42.91) | -3.33 (-4.2 to -2.45) |
| Samoa | 1 (0 to 1) | 1.42 (0.8 to 3.07) | 19 (10 to 27) | 37.92 (20.86 to 55.6) | 10.84 (10.59 to 11.09) |
| San Marino | 0 (0 to 0) | 1.46 (1.03 to 2) | 0 (0 to 0) | 1.68 (1.23 to 2.19) | 0.26 (0.01 to 0.5) |
| Sao Tome and Principe | 0 (0 to 1) | 0.92 (0.26 to 4.36) | 0 (0 to 0) | 0.3 (0.11 to 0.64) | -3.54 (-4.32 to -2.75) |
| Saudi Arabia | 75 (35 to 181) | 2.22 (1.07 to 5.24) | 485 (102 to 1708) | 4.59 (1 to 15.62) | 1.65 (1.02 to 2.28) |
| Senegal | 1297 (684 to 2189) | 75.6 (39.93 to 127.35) | 1143 (461 to 2269) | 29.81 (12.01 to 59.14) | -4.41 (-5.1 to -3.72) |
| Serbia | 8 (0 to 15) | 0.36 (0 to 0.65) | 4 (2 to 8) | 0.2 (0.09 to 0.42) | -0.87 (-2.79 to 1.08) |
| Seychelles | 2 (1 to 3) | 12.63 (7.71 to 19.7) | 1 (1 to 2) | 5.78 (3.6 to 9.03) | -1.83 (-2.25 to -1.41) |
| Sierra Leone | 886 (221 to 2039) | 85.71 (21.39 to 197.81) | 1869 (726 to 4048) | 82.48 (31.98 to 178.67) | -1.07 (-1.92 to -0.21) |
| Singapore | 16 (8 to 28) | 1.65 (0.79 to 2.88) | 19 (7 to 36) | 1.28 (0.49 to 2.41) | -0.98 (-1.72 to -0.23) |
| Slovakia | 1 (1 to 1) | 0.08 (0.06 to 0.11) | 2 (1 to 3) | 0.16 (0.07 to 0.29) | 1.12 (0.65 to 1.59) |
| Slovenia | 1 (0 to 2) | 0.23 (0 to 0.43) | 1 (1 to 2) | 0.27 (0.14 to 0.56) | -0.78 (-2.43 to 0.89) |
| Solomon Islands | 1 (1 to 2) | 1.49 (0.84 to 3.15) | 67 (37 to 97) | 38.88 (21.51 to 56.47) | 10.67 (10.4 to 10.93) |
| Somalia | 491 (80 to 1582) | 29.22 (4.75 to 94.04) | 1401 (419 to 3330) | 30.24 (9.07 to 71.94) | -4.31 (-5.8 to -2.8) |
| South Africa | 29969 (20944 to 41089) | 300.58 (210.25 to 410.84) | 96291 (68794 to 128603) | 618.52 (441.2 to 827.48) | -1.79 (-3.58 to 0.04) |
| South Sudan | 1545 (201 to 5012) | 118.74 (15.43 to 385.25) | 4268 (726 to 12183) | 195.86 (33.5 to 560.19) | 0.18 (-0.59 to 0.95) |
| Spain | 2423 (1633 to 3409) | 25.12 (16.93 to 35.36) | 471 (274 to 713) | 5.07 (2.97 to 7.62) | -4.7 (-5.81 to -3.56) |
| Sri Lanka | 36 (3 to 186) | 0.78 (0.06 to 4.05) | 83 (34 to 175) | 1.51 (0.62 to 3.17) | 1.83 (1.19 to 2.47) |
| Sudan | 999 (93 to 3663) | 21.28 (1.98 to 77.93) | 7906 (692 to 30727) | 70.61 (6.18 to 274.92) | 2.32 (1.83 to 2.81) |
| Suriname | 35 (21 to 60) | 35.61 (21.41 to 60.5) | 130 (66 to 232) | 90.85 (46.28 to 162.77) | 1.53 (0.74 to 2.33) |
| Sweden | 204 (122 to 330) | 10.09 (6.01 to 16.39) | 174 (71 to 289) | 7.95 (3.34 to 13.15) | 0.81 (0 to 1.62) |
| Switzerland | 512 (262 to 844) | 29.24 (14.94 to 48.51) | 108 (38 to 211) | 5.38 (1.98 to 10.41) | -6.03 (-6.99 to -5.05) |
| Syrian Arab Republic | 4 (3 to 5) | 0.14 (0.1 to 0.19) | 10 (6 to 17) | 0.25 (0.14 to 0.42) | 0.93 (0.5 to 1.36) |
| Taiwan (Province of China) | 29 (17 to 53) | 0.52 (0.3 to 0.97) | 104 (26 to 223) | 2.16 (0.55 to 4.58) | 4.26 (2.94 to 5.59) |
| Tajikistan | 46 (28 to 74) | 4.23 (2.61 to 6.7) | 354 (141 to 662) | 14.51 (5.5 to 27.69) | 2.52 (1.7 to 3.36) |
| Thailand | 12644 (6492 to 20873) | 74.62 (38.55 to 123.01) | 5830 (2836 to 10527) | 39.31 (19.29 to 70.67) | -2.14 (-3.19 to -1.08) |
| Timor-Leste | 58 (42 to 77) | 30.01 (21.37 to 40.1) | 76 (52 to 106) | 21.51 (14.73 to 30.29) | -0.83 (-1.29 to -0.37) |
| Togo | 3428 (1856 to 5504) | 399.8 (217.36 to 642.23) | 1693 (811 to 3134) | 79.05 (37.84 to 146.22) | -6.19 (-6.8 to -5.58) |
| Tokelau | 0 (0 to 0) | 0.66 (0.35 to 1.48) | 0 (0 to 0) | 17.65 (9.45 to 26.61) | 10.56 (10.14 to 10.98) |
| Tonga | 0 (0 to 1) | 1.47 (0.82 to 3.17) | 11 (6 to 16) | 42.21 (23.56 to 61.95) | 11.2 (10.95 to 11.45) |
| Trinidad and Tobago | 188 (108 to 303) | 58.71 (33.89 to 94.55) | 524 (310 to 928) | 173.15 (105.53 to 299.56) | 1.8 (1.02 to 2.59) |
| Tunisia | 2 (0 to 5) | 0.09 (0.01 to 0.23) | 29 (13 to 50) | 0.94 (0.44 to 1.65) | 8.65 (7.02 to 10.3) |
| Turkey | 8 (0 to 22) | 0.06 (0 to 0.16) | 125 (61 to 225) | 0.64 (0.31 to 1.15) | 6.59 (5.54 to 7.64) |
| Turkmenistan | 42 (23 to 163) | 4.42 (2.34 to 17.42) | 28 (17 to 48) | 2.22 (1.34 to 3.78) | -1.71 (-2.09 to -1.32) |
| Tuvalu | 0 (0 to 0) | 0.66 (0.35 to 1.5) | 1 (0 to 1) | 18.42 (10 to 27.61) | 10.78 (10.44 to 11.12) |
| Uganda | 44824 (31456 to 59549) | 1108.42 (764.59 to 1495.28) | 29267 (13536 to 56276) | 277.52 (127.56 to 537.05) | -2.95 (-3.37 to -2.53) |
| Ukraine | 660 (407 to 1109) | 5.16 (3.14 to 8.75) | 11396 (5224 to 23503) | 104.74 (47.92 to 216.92) | 8.18 (6.54 to 9.84) |
| United Arab Emirates | 2 (1 to 3) | 0.43 (0.24 to 0.87) | 28 (16 to 45) | 1.78 (0.98 to 2.81) | 3.28 (2.16 to 4.43) |
| United Kingdom | 534 (319 to 839) | 3.78 (2.25 to 5.96) | 1522 (888 to 2279) | 10.09 (5.96 to 15.01) | 0.98 (-0.64 to 2.62) |
| United Republic of Tanzania | 89896 (55804 to 131112) | 1462.35 (895.61 to 2159.63) | 18092 (6059 to 41713) | 122.12 (40.44 to 283.65) | -5.76 (-6.83 to -4.68) |
| United States of America | 12672 (7144 to 18480) | 18.76 (10.52 to 27.54) | 14759 (5302 to 25077) | 19.44 (7.18 to 32.83) | 0.69 (0.31 to 1.07) |
| United States Virgin Islands | 5 (3 to 12) | 18.16 (10.4 to 41.87) | 1 (1 to 2) | 8.06 (4.6 to 14.17) | -2.35 (-2.69 to -2.01) |
| Uruguay | 47 (16 to 152) | 6.32 (2.18 to 20.35) | 227 (87 to 449) | 28.35 (10.91 to 55.79) | 3.9 (3.27 to 4.53) |
| Uzbekistan | 131 (43 to 255) | 2.6 (0.87 to 5.08) | 3357 (892 to 7886) | 36.2 (9.95 to 84.47) | 10.13 (8.8 to 11.47) |
| Vanuatu | 1 (0 to 1) | 1.41 (0.79 to 3.05) | 30 (17 to 44) | 38.1 (21.34 to 55.37) | 10.77 (10.48 to 11.05) |
| Venezuela (Bolivarian Republic of) | 250 (170 to 378) | 5.14 (3.51 to 7.78) | 1009 (524 to 1808) | 15.96 (8.25 to 28.49) | 1.82 (0.74 to 2.91) |
| Viet Nam | 629 (450 to 837) | 3.31 (2.36 to 4.4) | 2627 (1440 to 4912) | 10.83 (6.04 to 19.81) | 3.51 (2.54 to 4.48) |
| Yemen | 12 (5 to 29) | 0.45 (0.18 to 1.04) | 357 (143 to 670) | 4.24 (1.7 to 7.97) | 7.57 (6.7 to 8.46) |
| Zambia | 49881 (33934 to 68225) | 2571.11 (1715.18 to 3585.79) | 19948 (10343 to 34732) | 390.69 (200.61 to 685.47) | -5.13 (-5.44 to -4.81) |
| Zimbabwe | 108117 (79364 to 138712) | 4371.18 (3162.45 to 5674.89) | 10525 (6110 to 16426) | 255.91 (148.1 to 400.57) | -7.84 (-8.43 to -7.26) |

**Supplementary Table S2**

The prevalence of HIV/AIDS cases and rates among women of childbearing age across 204 countries in 1990 and 2021, and the trends from 1990 to 2021

| location | Num_1990 | ASR_1990 | Num_2021 | ASR_2021 | EAPC_CI |
| --- | --- | --- | --- | --- | --- |
| Afghanistan | 63 (27 to 106) | 3.37 (1.44 to 5.68) | 1021 (544 to 1559) | 16.3 (8.95 to 24.52) | 5.19 (4.96 to 5.43) |
| Albania | 9 (6 to 12) | 1.06 (0.74 to 1.42) | 8 (4 to 16) | 1.28 (0.65 to 2.61) | -0.53 (-0.99 to -0.07) |
| Algeria | 141 (118 to 167) | 2.54 (2.13 to 3.01) | 7440 (6320 to 8654) | 62.69 (53.04 to 73.23) | 11.33 (11.01 to 11.65) |
| American Samoa | 0 (0 to 1) | 3.15 (1.94 to 4.24) | 14 (10 to 18) | 124.03 (84.53 to 163.8) | 12.38 (12.17 to 12.6) |
| Andorra | 1 (1 to 1) | 5.72 (4.58 to 6.99) | 5 (4 to 5) | 18.37 (14.74 to 22.05) | 3.73 (3.17 to 4.29) |
| Angola | 4211 (1229 to 9159) | 185.15 (54.05 to 403.4) | 266291 (179857 to 376909) | 3839.55 (2634.76 to 5362.26) | 8.12 (6.84 to 9.42) |
| Antigua and Barbuda | 11 (8 to 17) | 70.87 (47.66 to 103.67) | 65 (44 to 101) | 259.3 (170.96 to 404.84) | 3.34 (2.94 to 3.73) |
| Argentina | 8202 (6677 to 9976) | 103.53 (84.35 to 125.82) | 29170 (24966 to 33731) | 235.99 (201.61 to 273.39) | 2.41 (2.07 to 2.75) |
| Armenia | 3 (0 to 5) | 0.3 (0 to 0.53) | 424 (283 to 653) | 48.66 (32.17 to 75.13) | 20.12 (19.27 to 20.97) |
| Australia | 532 (312 to 782) | 11.7 (6.85 to 17.22) | 2372 (1142 to 4061) | 36.68 (17.77 to 62.73) | 3.83 (3.65 to 4) |
| Austria | 326 (185 to 505) | 15.99 (9.05 to 24.79) | 1873 (836 to 3281) | 86.36 (38.39 to 151.2) | 4.39 (3.97 to 4.81) |
| Azerbaijan | 57 (36 to 82) | 2.87 (1.81 to 4.07) | 680 (394 to 1138) | 22.69 (13.12 to 37.86) | 5.53 (5.02 to 6.04) |
| Bahamas | 424 (283 to 601) | 576.49 (385.18 to 813.99) | 1331 (744 to 2222) | 1231.78 (684.5 to 2058.75) | 1.12 (0.72 to 1.53) |
| Bahrain | 6 (4 to 9) | 5.06 (3.47 to 7.14) | 47 (36 to 64) | 13.76 (10.66 to 18.95) | 2.81 (2.05 to 3.57) |
| Bangladesh | 9 (7 to 11) | 0.04 (0.03 to 0.05) | 4326 (3484 to 5482) | 9.44 (7.6 to 11.99) | 20.19 (17.37 to 23.08) |
| Barbados | 70 (43 to 110) | 99.33 (62.11 to 155.78) | 424 (327 to 571) | 571.31 (436.82 to 774.98) | 5.25 (4.66 to 5.85) |
| Belarus | 262 (0 to 400) | 9.86 (0 to 15.05) | 6941 (4389 to 11502) | 276.9 (173.51 to 465.75) | 11.66 (11.24 to 12.08) |
| Belgium | 607 (322 to 951) | 24.1 (12.75 to 37.82) | 4425 (2525 to 6447) | 164.18 (93.69 to 239.29) | 4.94 (4.1 to 5.8) |
| Belize | 48 (36 to 63) | 125.74 (95.04 to 162.62) | 759 (418 to 1320) | 644.72 (356.25 to 1120.51) | 4.08 (3.11 to 5.06) |
| Benin | 1246 (695 to 1943) | 112.27 (62.87 to 174.34) | 39440 (28761 to 52224) | 1446.39 (1076.29 to 1885.63) | 3.49 (1.45 to 5.57) |
| Bermuda | 14 (10 to 21) | 78.82 (53.09 to 113.79) | 20 (15 to 29) | 135.41 (98.39 to 198.75) | 0.56 (0.16 to 0.95) |
| Bhutan | 10 (5 to 16) | 7.22 (3.81 to 11.76) | 232 (131 to 339) | 112.73 (64.49 to 164.2) | 8.47 (7.41 to 9.55) |
| Bolivia (Plurinational State of) | 81 (16 to 243) | 5.3 (1.07 to 15.99) | 5259 (3903 to 6763) | 172.22 (128.4 to 220.84) | 7.42 (4.22 to 10.73) |
| Bosnia and Herzegovina | 5 (4 to 6) | 0.39 (0.3 to 0.5) | 6 (4 to 9) | 0.78 (0.49 to 1.21) | 1.45 (0.81 to 2.1) |
| Botswana | 22582 (16976 to 30072) | 6866.6 (5126.43 to 9163.95) | 155940 (127070 to 189957) | 23081.94 (18875.6 to 28005.01) | 1.93 (0.74 to 3.13) |
| Brazil | 40382 (27145 to 58185) | 101.75 (68.72 to 146.46) | 222242 (128896 to 357953) | 357.74 (207.3 to 576.29) | 3.54 (3.27 to 3.81) |
| Brunei Darussalam | 7 (3 to 12) | 10.84 (5.4 to 18.82) | 95 (47 to 162) | 71.99 (35.92 to 122.51) | 6.52 (6.23 to 6.82) |
| Bulgaria | 46 (0 to 80) | 2.25 (0 to 3.85) | 416 (282 to 583) | 31.59 (20.9 to 44.7) | 7.78 (6.96 to 8.6) |
| Burkina Faso | 97693 (69732 to 129865) | 4751.56 (3391.31 to 6305.19) | 39531 (26826 to 52642) | 859.24 (592.59 to 1124.47) | -5.94 (-6.18 to -5.71) |
| Burundi | 65339 (18354 to 158414) | 5211.17 (1456.94 to 12667.67) | 38712 (29312 to 50735) | 1531.7 (1177.42 to 1982.38) | -5.68 (-6.36 to -4.99) |
| Cabo Verde | 412 (119 to 794) | 525.05 (151.17 to 1010.08) | 1324 (555 to 3097) | 902.56 (387.55 to 2083.38) | 0.32 (-0.33 to 0.97) |
| Cambodia | 13 (9 to 17) | 0.49 (0.35 to 0.67) | 23300 (15448 to 29003) | 540.45 (359.58 to 670.2) | 10.85 (5.2 to 16.82) |
| Cameroon | 25062 (15890 to 37050) | 1048.6 (663.2 to 1551.99) | 366511 (298407 to 447877) | 5322.49 (4371 to 6439.53) | 3.02 (1.58 to 4.48) |
| Canada | 2954 (1199 to 5445) | 38.39 (15.46 to 71.37) | 15646 (7357 to 25837) | 172.83 (81.44 to 285.59) | 4.67 (4.25 to 5.09) |
| Central African Republic | 43638 (25590 to 68354) | 6791.28 (4004.03 to 10598.05) | 65736 (42813 to 99372) | 5399.55 (3600.44 to 8007.48) | -2.54 (-3.07 to -2) |
| Chad | 11372 (5460 to 20674) | 863.82 (413.82 to 1571.83) | 61457 (40689 to 84730) | 1946.86 (1326.9 to 2624.04) | 0.86 (0.03 to 1.7) |
| Chile | 737 (470 to 1063) | 20.24 (13.04 to 29.03) | 8262 (4123 to 14346) | 166.07 (82.69 to 288.72) | 6.94 (6.52 to 7.35) |
| China | 10468 (5937 to 14424) | 3.36 (1.94 to 4.59) | 65053 (31957 to 129926) | 19.13 (9.31 to 38.05) | 5.48 (4.35 to 6.62) |
| Colombia | 1077 (529 to 2687) | 12.49 (6.3 to 30.46) | 28391 (17742 to 44003) | 212.77 (132.94 to 329.87) | 7.98 (7.12 to 8.85) |
| Comoros | 1 (0 to 3) | 1.04 (0.23 to 3.07) | 17 (8 to 26) | 9.41 (4.63 to 14.31) | 7.56 (6.15 to 9) |
| Congo | 26380 (15728 to 39930) | 4753.16 (2845.58 to 7146.54) | 53610 (34335 to 85133) | 3920.35 (2528.37 to 6176.73) | -1.56 (-1.84 to -1.29) |
| Cook Islands | 0 (0 to 0) | 2.7 (1.73 to 3.77) | 5 (4 to 7) | 121.61 (84.49 to 159.17) | 12.83 (12.51 to 13.16) |
| Costa Rica | 131 (85 to 207) | 17.16 (11.17 to 27.09) | 910 (493 to 1474) | 70.02 (37.8 to 113.26) | 4.1 (3.69 to 4.51) |
| Coted'Ivoire | 185049 (102750 to 299468) | 6819.85 (3771.67 to 11122.73) | 244884 (191962 to 303064) | 4142.43 (3288.34 to 5069.76) | -2.88 (-3.37 to -2.38) |
| Croatia | 19 (12 to 28) | 1.55 (0.94 to 2.24) | 84 (47 to 141) | 8.6 (4.82 to 14.53) | 5.14 (4.75 to 5.53) |
| Cuba | 289 (211 to 407) | 9.34 (6.9 to 13.14) | 3399 (1733 to 6552) | 125.77 (63.37 to 245.7) | 10.11 (9.33 to 10.91) |
| Cyprus | 4 (2 to 6) | 1.84 (1.07 to 2.85) | 47 (24 to 83) | 14.85 (7.79 to 26.27) | 5.98 (5.58 to 6.39) |
| Czechia | 22 (14 to 34) | 0.84 (0.52 to 1.34) | 261 (108 to 457) | 11.92 (5.09 to 20.46) | 8.13 (7.45 to 8.81) |
| Democratic People's Republic of Korea | 37 (5 to 81) | 0.66 (0.09 to 1.44) | 4900 (2181 to 8694) | 70.77 (30.83 to 126.64) | 14.38 (12.09 to 16.72) |
| Democratic Republic of the Congo | 210527 (146830 to 295225) | 2527.46 (1771.88 to 3540.34) | 188906 (141460 to 245321) | 1041.27 (792 to 1337.74) | -4.01 (-4.58 to -3.43) |
| Denmark | 246 (191 to 309) | 18.64 (14.48 to 23.35) | 326 (266 to 390) | 23.39 (19.07 to 28.04) | 0.58 (0.12 to 1.04) |
| Djibouti | 26 (3 to 76) | 26.94 (3.28 to 78.39) | 7307 (3766 to 13473) | 2249.01 (1163.33 to 4141.31) | 8.07 (4.16 to 12.13) |
| Dominica | 10 (7 to 14) | 62.09 (44.38 to 83.09) | 27 (17 to 46) | 165.94 (104.2 to 281.87) | 2.01 (1.62 to 2.4) |
| Dominican Republic | 6327 (2761 to 11973) | 330.21 (143.27 to 624.67) | 21025 (11452 to 31623) | 738.57 (404.47 to 1105.83) | 0.33 (-0.47 to 1.14) |
| Ecuador | 438 (280 to 689) | 17.33 (11.38 to 26.52) | 11755 (7552 to 18399) | 249.37 (161.23 to 388.49) | 9.15 (8.8 to 9.49) |
| Egypt | 197 (144 to 249) | 1.54 (1.13 to 1.94) | 2577 (1186 to 4892) | 9.79 (4.53 to 18.55) | 5.78 (5.4 to 6.16) |
| El Salvador | 351 (220 to 544) | 27.49 (17.48 to 42.53) | 3933 (2220 to 6652) | 223.7 (126.21 to 378.85) | 6.28 (5.11 to 7.47) |
| Equatorial Guinea | 461 (124 to 1116) | 475.77 (128.48 to 1150.94) | 46570 (22630 to 82551) | 13872.69 (6967.98 to 24085.46) | 9.35 (8.05 to 10.67) |
| Eritrea | 9075 (2158 to 19665) | 1174.34 (278.48 to 2539.66) | 10721 (7392 to 15220) | 717.8 (502.22 to 1010.21) | -3.89 (-4.88 to -2.89) |
| Estonia | 3 (1 to 5) | 0.73 (0.36 to 1.33) | 953 (606 to 1607) | 321.43 (203.31 to 539.88) | 22.22 (18.8 to 25.75) |
| Eswatini | 423 (64 to 1480) | 203.94 (31.34 to 712.88) | 98096 (87487 to 108924) | 33810.75 (30340.38 to 37315.51) | 11.43 (7.92 to 15.04) |
| Ethiopia | 116716 (49713 to 204758) | 1040.25 (441.24 to 1832.9) | 339176 (256204 to 455111) | 1481.6 (1134.25 to 1952.58) | -1 (-1.95 to -0.05) |
| Fiji | 26 (16 to 36) | 13.16 (8.44 to 18.56) | 223 (120 to 384) | 97.38 (52.59 to 167.84) | 5.78 (5.11 to 6.45) |
| Finland | 80 (49 to 117) | 5.91 (3.6 to 8.65) | 330 (170 to 532) | 26.62 (13.78 to 42.96) | 4.72 (4.43 to 5.02) |
| France | 13137 (10231 to 16402) | 88.61 (68.91 to 110.78) | 27305 (22215 to 32419) | 177.04 (143.34 to 210.94) | 2.59 (2.38 to 2.8) |
| Gabon | 2992 (1496 to 4673) | 1366.07 (687.17 to 2127.71) | 28693 (18166 to 42226) | 6493.67 (4187.43 to 9398.52) | 3.55 (2.59 to 4.53) |
| Gambia | 432 (128 to 867) | 191.54 (56.71 to 385.12) | 16937 (10012 to 26167) | 3168.64 (1915.59 to 4799.74) | 6.96 (5.36 to 8.6) |
| Georgia | 12 (5 to 25) | 0.84 (0.38 to 1.78) | 557 (313 to 1080) | 61.34 (34.13 to 118.34) | 17.03 (15.9 to 18.17) |
| Germany | 7291 (4658 to 10503) | 35.85 (22.86 to 51.75) | 13401 (7182 to 22595) | 70.18 (37.55 to 118.2) | 2.39 (2.26 to 2.52) |
| Ghana | 40362 (27771 to 54661) | 1151.8 (792.92 to 1556.97) | 214564 (163609 to 277276) | 2513.5 (1938.43 to 3220.85) | 0.86 (0 to 1.73) |
| Greece | 201 (141 to 270) | 8.03 (5.63 to 10.79) | 1024 (618 to 1471) | 43.95 (27 to 62.87) | 6.08 (5.84 to 6.32) |
| Greenland | 23 (15 to 33) | 143.48 (96.61 to 212.63) | 37 (14 to 71) | 280.13 (109.43 to 541.47) | 2.31 (2.17 to 2.45) |
| Grenada | 10 (7 to 14) | 52.34 (36.52 to 72.72) | 24 (17 to 35) | 91.82 (65.08 to 138.11) | 0.82 (0.3 to 1.34) |
| Guam | 1 (1 to 2) | 3.39 (2.2 to 5.19) | 18 (12 to 30) | 50.04 (32.05 to 83.15) | 8.19 (7.39 to 8.99) |
| Guatemala | 930 (631 to 1259) | 53.42 (36.81 to 71.6) | 4093 (2302 to 7082) | 96.34 (54.28 to 166.83) | 0.91 (0.29 to 1.52) |
| Guinea | 6111 (2424 to 11399) | 444.1 (176.68 to 827.07) | 65129 (47772 to 85544) | 2224.92 (1662.27 to 2887.53) | 3.24 (2.07 to 4.43) |
| Guinea-Bissau | 811 (151 to 1711) | 353.95 (66.54 to 744.66) | 24351 (4452 to 48020) | 5097.61 (987.28 to 9848.95) | 6.61 (5.38 to 7.85) |
| Guyana | 397 (267 to 588) | 193.96 (131.66 to 285.95) | 2057 (1226 to 3311) | 1035.5 (618.95 to 1663.69) | 4.56 (3.38 to 5.74) |
| Haiti | 57236 (31262 to 88312) | 3719 (2029.7 to 5739.16) | 98594 (67536 to 129449) | 2854.25 (1966.08 to 3734.11) | -1.6 (-1.83 to -1.37) |
| Honduras | 550 (397 to 737) | 53.66 (39.11 to 71.44) | 955 (570 to 1562) | 34.37 (20.41 to 56.29) | -2.96 (-3.41 to -2.51) |
| Hungary | 212 (147 to 296) | 7.85 (5.45 to 11.01) | 156 (101 to 223) | 6.4 (4.21 to 9.15) | -1.39 (-2.14 to -0.62) |
| Iceland | 14 (9 to 20) | 20.7 (12.76 to 30.31) | 78 (42 to 126) | 92.58 (49.41 to 148.93) | 5.16 (4.91 to 5.42) |
| India | 9409 (6032 to 14059) | 4.64 (2.97 to 6.93) | 593074 (481461 to 718490) | 159.89 (129.98 to 193.47) | 6.12 (3 to 9.33) |
| Indonesia | 446 (289 to 613) | 0.93 (0.6 to 1.28) | 42272 (27512 to 69952) | 56.06 (36.37 to 92.85) | 12.93 (11.35 to 14.54) |
| Iran (Islamic Republic of) | 90 (53 to 151) | 0.76 (0.46 to 1.26) | 5904 (4039 to 8353) | 23.83 (16.11 to 33.89) | 12.06 (11.25 to 12.88) |
| Iraq | 51 (26 to 101) | 1.31 (0.68 to 2.52) | 1153 (405 to 2647) | 10.91 (3.81 to 25.37) | 6.76 (6.11 to 7.4) |
| Ireland | 88 (50 to 138) | 10.2 (5.76 to 15.93) | 1023 (519 to 1706) | 81.17 (41.15 to 134.66) | 5.99 (5.32 to 6.67) |
| Israel | 187 (100 to 314) | 15.55 (8.31 to 26.11) | 2488 (1347 to 3724) | 110.06 (59.55 to 164.61) | 6.1 (5.64 to 6.56) |
| Italy | 15940 (10431 to 22463) | 111.68 (73.07 to 157.4) | 20464 (12738 to 30261) | 142.35 (89.27 to 209.09) | 0.74 (0.42 to 1.07) |
| Jamaica | 796 (453 to 1377) | 138.22 (80.35 to 235.58) | 4454 (2525 to 7507) | 567.94 (323.23 to 953.77) | 3.45 (2.93 to 3.97) |
| Japan | 672 (336 to 1185) | 2.09 (1.04 to 3.66) | 4301 (2388 to 6420) | 16.84 (9.59 to 24.99) | 6.64 (6.34 to 6.93) |
| Jordan | 8 (5 to 13) | 1.08 (0.72 to 1.63) | 80 (55 to 121) | 2.68 (1.85 to 4.07) | 2.19 (1.6 to 2.78) |
| Kazakhstan | 261 (177 to 367) | 6.02 (4.07 to 8.45) | 6975 (4601 to 11128) | 134.27 (88.43 to 214.53) | 8.73 (8.03 to 9.42) |
| Kenya | 316014 (259529 to 376514) | 6275.59 (5155.34 to 7451.5) | 699499 (580405 to 806901) | 6154.29 (5143.3 to 7066.09) | -2.03 (-2.83 to -1.22) |
| Kiribati | 3 (2 to 3) | 14.43 (11.89 to 17.33) | 4 (2 to 7) | 11.8 (5.55 to 22.05) | -1.36 (-1.57 to -1.14) |
| Kuwait | 4 (3 to 5) | 0.87 (0.63 to 1.15) | 18 (11 to 30) | 1.1 (0.65 to 1.86) | 0.14 (-0.27 to 0.54) |
| Kyrgyzstan | 66 (43 to 99) | 6.36 (4.16 to 9.43) | 3001 (1760 to 4889) | 172.75 (102.16 to 279.71) | 10.93 (10.76 to 11.1) |
| Lao People's Democratic Republic | 3 (2 to 4) | 0.28 (0.21 to 0.37) | 2965 (2389 to 3579) | 156.18 (126.57 to 187.79) | 19.68 (15.19 to 24.35) |
| Latvia | 122 (83 to 170) | 18.08 (12.13 to 25.28) | 1686 (1266 to 2221) | 380.29 (284.12 to 504.97) | 8.6 (8.06 to 9.14) |
| Lebanon | 32 (26 to 38) | 4.28 (3.57 to 5.09) | 490 (398 to 592) | 30.6 (24.71 to 37.15) | 6.66 (6.25 to 7.07) |
| Lesotho | 11881 (7888 to 16875) | 3165.71 (2098.86 to 4499.54) | 158743 (134754 to 181094) | 34674.41 (29692.61 to 39180.09) | 5.54 (4.02 to 7.09) |
| Liberia | 2193 (645 to 5489) | 388.67 (114.34 to 974.21) | 24156 (17347 to 33822) | 1905.95 (1380.22 to 2643.03) | 2.85 (1.48 to 4.24) |
| Libya | 44 (4 to 171) | 5.4 (0.43 to 20.57) | 637 (15 to 3667) | 31.03 (0.71 to 178.55) | 5.41 (4.68 to 6.14) |
| Lithuania | 276 (194 to 392) | 29.22 (20.54 to 41.55) | 492 (328 to 782) | 73.82 (48.31 to 118.97) | 2.38 (2.02 to 2.74) |
| Luxembourg | 33 (19 to 50) | 31.86 (18.21 to 48.48) | 179 (87 to 307) | 102.59 (49.88 to 176.33) | 3.68 (3.52 to 3.84) |
| Madagascar | 41 (16 to 79) | 1.54 (0.59 to 2.94) | 26064 (13499 to 44891) | 394.35 (207.09 to 672.39) | 14.57 (10.92 to 18.34) |
| Malawi | 217115 (150392 to 294729) | 9708.26 (6700.29 to 13226.76) | 543587 (461012 to 625095) | 13336.23 (11410.81 to 15183.54) | -0.01 (-0.49 to 0.46) |
| Malaysia | 674 (389 to 1029) | 15.93 (9.25 to 24.28) | 11891 (6719 to 19455) | 139.78 (79.03 to 228.7) | 4.6 (3.73 to 5.47) |
| Maldives | 2 (1 to 3) | 4.24 (2.57 to 6.25) | 9 (5 to 15) | 7.03 (4.45 to 11.82) | 0.68 (0.09 to 1.27) |
| Mali | 9212 (3613 to 21650) | 479.9 (188.74 to 1127.34) | 60000 (43278 to 83141) | 1337.36 (990.9 to 1809.94) | 1.36 (0.24 to 2.49) |
| Malta | 13 (8 to 18) | 13.31 (8.55 to 18.86) | 111 (53 to 187) | 104.27 (50.32 to 174.73) | 7.08 (6.88 to 7.28) |
| Marshall Islands | 1 (0 to 1) | 6.67 (4.35 to 9.03) | 32 (22 to 42) | 221.89 (151.76 to 286.89) | 11.4 (11.07 to 11.73) |
| Mauritania | 13 (4 to 30) | 2.84 (1.03 to 6.68) | 73 (18 to 97) | 8.05 (2.05 to 10.66) | 2.57 (0.99 to 4.17) |
| Mauritius | 18 (15 to 22) | 6.05 (5.11 to 7.25) | 617 (430 to 913) | 189.69 (132.47 to 280.44) | 13.95 (13.21 to 14.7) |
| Mexico | 5357 (3831 to 7336) | 26.03 (18.82 to 35.32) | 33960 (22406 to 49355) | 96.13 (63.15 to 140.1) | 3.28 (3.01 to 3.55) |
| Micronesia (Federated States of) | 2 (1 to 2) | 7.76 (4.49 to 10.54) | 58 (40 to 76) | 232.97 (160.63 to 302.09) | 10.92 (10.61 to 11.23) |
| Monaco | 1 (1 to 1) | 9.85 (8 to 11.97) | 2 (2 to 3) | 30.22 (24.99 to 35.53) | 3.59 (3.2 to 3.98) |
| Mongolia | 0 (0 to 0) | 0.01 (0 to 0) | 52 (15 to 135) | 5.75 (1.65 to 14.85) | 14.24 (10.18 to 18.44) |
| Montenegro | 3 (2 to 4) | 1.92 (1.27 to 2.73) | 17 (12 to 22) | 11.78 (8.37 to 16.1) | 4.84 (4.36 to 5.33) |
| Morocco | 781 (526 to 1225) | 12.61 (8.48 to 19.83) | 7388 (5817 to 9005) | 74.77 (58.77 to 91.26) | 5.2 (4.35 to 6.06) |
| Mozambique | 40470 (22271 to 69744) | 1260.04 (695.5 to 2170.54) | 1112764 (888847 to 1417120) | 16752.91 (13721.76 to 20827.73) | 6.8 (5.49 to 8.13) |
| Myanmar | 3102 (1872 to 4617) | 29.19 (17.53 to 43.64) | 88309 (52466 to 112616) | 589.05 (349.66 to 750.87) | 5.31 (3.24 to 7.42) |
| Namibia | 6211 (4297 to 8693) | 1883.4 (1301.87 to 2634.17) | 89352 (76769 to 102188) | 14759.35 (12736.39 to 16801.32) | 4.31 (2.71 to 5.94) |
| Nauru | 0 (0 to 0) | 3.24 (1.94 to 4.38) | 3 (2 to 4) | 119.88 (84.37 to 156.66) | 11.74 (11.37 to 12.1) |
| Nepal | 0 to 0 | 0 (0 to 0) | 7721 (6131 to 9440) | 91.28 (72.95 to 111.02) | 19.88 (13.34 to 26.8) |
| Netherlands | 1038 (640 to 1535) | 25.68 (15.82 to 38) | 3112 (1725 to 4921) | 80.13 (44.48 to 126.79) | 4.24 (3.84 to 4.64) |
| New Zealand | 74 (43 to 111) | 8.11 (4.71 to 12.23) | 389 (222 to 644) | 31.17 (17.77 to 51.59) | 4.85 (4.58 to 5.13) |
| Nicaragua | 74 (53 to 105) | 8.3 (6.02 to 11.62) | 2259 (1167 to 3897) | 123.89 (64.17 to 213.44) | 8.77 (8.43 to 9.12) |
| Niger | 4524 (1554 to 9031) | 265.22 (91.22 to 528.03) | 15308 (9222 to 24328) | 373.07 (231.77 to 578.8) | -1.39 (-2.66 to -0.11) |
| Nigeria | 123375 (91885 to 163001) | 628.47 (467.64 to 834.65) | 1043247 (872894 to 1218457) | 2159.11 (1817.95 to 2503.62) | 2.16 (1.23 to 3.09) |
| Niue | 0 (0 to 0) | 3.23 (2.02 to 4.34) | 0 (0 to 1) | 118.46 (82.19 to 156.02) | 11.98 (11.7 to 12.26) |
| North Macedonia | 2 (1 to 2) | 0.31 (0.22 to 0.42) | 6 (4 to 10) | 1.04 (0.62 to 1.73) | 3.15 (2.72 to 3.59) |
| Northern Mariana Islands | 1 (1 to 2) | 7.55 (5.18 to 10.84) | 8 (5 to 12) | 66.72 (42.13 to 109.52) | 6.43 (5.73 to 7.14) |
| Norway | 83 (53 to 117) | 7.8 (4.99 to 11.03) | 1517 (823 to 2441) | 122.64 (66.76 to 197.41) | 8.41 (8.08 to 8.75) |
| Oman | 12 (8 to 18) | 3.76 (2.58 to 5.47) | 506 (214 to 854) | 46.41 (19.78 to 77.87) | 7.79 (6.89 to 8.7) |
| Pakistan | 3 (0 to 14) | 0.01 (0 to 0.06) | 47800 (1211 to 239687) | 79.97 (2.04 to 403.63) | 35.52 (32.24 to 38.88) |
| Palau | 0 (0 to 0) | 2.9 (1.91 to 3.94) | 4 (3 to 5) | 110.74 (76.29 to 147.04) | 12.17 (11.92 to 12.42) |
| Palestine | 1 (1 to 2) | 0.35 (0.22 to 0.55) | 167 (94 to 273) | 13.87 (7.78 to 22.66) | 13.53 (12.31 to 14.76) |
| Panama | 504 (312 to 785) | 82.69 (51.78 to 127.75) | 4576 (2959 to 6936) | 429.17 (277.58 to 650.97) | 3.45 (2.87 to 4.04) |
| Papua New Guinea | 38 (19 to 65) | 4.02 (1.94 to 6.8) | 25343 (19080 to 32437) | 1019.15 (770.26 to 1299.34) | 14.2 (10.57 to 17.94) |
| Paraguay | 131 (59 to 233) | 14.18 (6.45 to 24.85) | 2671 (1520 to 4525) | 141.61 (81.02 to 239.27) | 6.84 (5.89 to 7.78) |
| Peru | 2035 (1237 to 3246) | 36.99 (22.99 to 58.13) | 14277 (8513 to 22308) | 145.27 (86.69 to 226.93) | 3.77 (3.45 to 4.09) |
| Philippines | 50 (24 to 100) | 0.34 (0.17 to 0.67) | 46347 (21756 to 89740) | 155.81 (73.58 to 302.14) | 13.54 (10.81 to 16.33) |
| Poland | 130 (66 to 232) | 1.4 (0.7 to 2.51) | 2847 (1523 to 4813) | 27.43 (14.71 to 46.06) | 8.11 (6.66 to 9.59) |
| Portugal | 701 (535 to 890) | 27.93 (21.36 to 35.46) | 4306 (3511 to 5148) | 148.28 (119.9 to 178.94) | 3.24 (1.89 to 4.61) |
| Puerto Rico | 1965 (1438 to 2539) | 206.84 (151.39 to 267.23) | 681 (483 to 1022) | 84.74 (59.38 to 128.72) | -4.46 (-4.92 to -3.99) |
| Qatar | 8 (5 to 11) | 9.3 (5.99 to 13.12) | 35 (26 to 47) | 5.66 (4.15 to 7.62) | -2.5 (-2.9 to -2.1) |
| Republic of Korea | 294 (0 to 476) | 2.34 (0 to 3.76) | 873 (462 to 1445) | 6.98 (3.65 to 11.57) | 3.63 (3.29 to 3.96) |
| Republic of Moldova | 99 (50 to 174) | 8.3 (4.21 to 14.82) | 2705 (1608 to 4373) | 259.38 (153.49 to 419.58) | 12.08 (10.92 to 13.25) |
| Romania | 384 (239 to 537) | 6.91 (4.28 to 9.65) | 4515 (2641 to 7124) | 111.34 (64.67 to 176.77) | 8.8 (7.51 to 10.11) |
| Russian Federation | 6220 (4423 to 8951) | 16.15 (11.45 to 23.26) | 309974 (222297 to 439870) | 821.08 (583.88 to 1180.91) | 14.89 (14.41 to 15.37) |
| Rwanda | 37121 (9021 to 81386) | 2319.26 (567.18 to 5074.77) | 116063 (90337 to 144152) | 3717.41 (2928.49 to 4574.64) | -0.11 (-0.83 to 0.61) |
| Saint Kitts and Nevis | 7 (4 to 11) | 68.52 (40.13 to 104.2) | 78 (62 to 96) | 475.09 (378.53 to 583.04) | 4.99 (4.02 to 5.96) |
| Saint Lucia | 20 (14 to 26) | 60.9 (44.55 to 79.7) | 40 (23 to 70) | 85.77 (49.27 to 151.56) | -0.52 (-1.06 to 0.02) |
| Saint Vincent and the Grenadines | 47 (30 to 67) | 183.29 (122.39 to 262.22) | 89 (53 to 157) | 318.68 (186.29 to 566.16) | -0.21 (-0.94 to 0.53) |
| Samoa | 3 (2 to 3) | 7.26 (4.45 to 9.74) | 105 (71 to 138) | 226.71 (154.93 to 294.81) | 11.31 (11.07 to 11.55) |
| San Marino | 1 (0 to 1) | 9.65 (7.76 to 11.77) | 2 (2 to 3) | 30.26 (25.17 to 35.46) | 3.57 (3.13 to 4.02) |
| Sao Tome and Principe | 1 (0 to 2) | 3.56 (1.79 to 6.56) | 3 (2 to 5) | 6.02 (3.92 to 9.2) | 0.08 (-0.62 to 0.79) |
| Saudi Arabia | 425 (246 to 727) | 13.52 (8.14 to 22.34) | 5359 (2342 to 13279) | 48.69 (21.3 to 119.32) | 3.7 (3.14 to 4.26) |
| Senegal | 4231 (2602 to 6714) | 252.74 (155.65 to 400.44) | 27455 (19140 to 37887) | 806.1 (570.7 to 1096.87) | 1.95 (0.83 to 3.08) |
| Serbia | 128 (0 to 211) | 5.44 (0 to 9.03) | 60 (32 to 132) | 2.7 (1.44 to 5.98) | -1.39 (-2.2 to -0.57) |
| Seychelles | 3 (2 to 4) | 16.32 (11.03 to 23.72) | 18 (11 to 31) | 71.88 (43.06 to 124.47) | 2.6 (1.57 to 3.64) |
| Sierra Leone | 2522 (854 to 5844) | 245.92 (83.33 to 569.57) | 38407 (26125 to 54234) | 1942.54 (1349.92 to 2692.07) | 4.75 (3.48 to 6.04) |
| Singapore | 35 (17 to 59) | 3.67 (1.79 to 6.16) | 541 (272 to 887) | 30.43 (15.44 to 49.71) | 4.82 (3.79 to 5.86) |
| Slovakia | 6 (4 to 8) | 0.44 (0.31 to 0.61) | 25 (16 to 40) | 1.85 (1.12 to 3.01) | 4.07 (3.79 to 4.35) |
| Slovenia | 2 (0 to 4) | 0.46 (0 to 0.76) | 23 (13 to 41) | 4.67 (2.65 to 8.4) | 6.7 (5.84 to 7.56) |
| Solomon Islands | 6 (3 to 8) | 7.82 (4.51 to 10.61) | 376 (257 to 489) | 227.17 (156.65 to 294.57) | 10.8 (10.51 to 11.09) |
| Somalia | 749 (236 to 2072) | 44.88 (14.19 to 123.85) | 17621 (7566 to 35657) | 412.07 (175.35 to 830.14) | 2.52 (-0.13 to 5.23) |
| South Africa | 64749 (51755 to 79695) | 655.89 (524.72 to 805.15) | 4237370 (3981885 to 4509134) | 26684.61 (25064.63 to 28413.31) | 8.89 (6.49 to 11.34) |
| South Sudan | 4063 (963 to 12053) | 319.7 (75.48 to 953.15) | 62376 (22026 to 122564) | 3037.44 (1086.6 to 5932.18) | 5.12 (3.77 to 6.49) |
| Spain | 14635 (10647 to 19177) | 152.7 (111.11 to 199.98) | 12381 (9059 to 16040) | 99.47 (70.87 to 131.19) | -2.13 (-2.57 to -1.7) |
| Sri Lanka | 149 (81 to 240) | 3.29 (1.81 to 5.28) | 779 (358 to 1486) | 13.86 (6.33 to 26.48) | 3.16 (2.7 to 3.62) |
| Sudan | 2772 (367 to 10898) | 60.49 (8.01 to 238.47) | 55932 (14098 to 157672) | 522.02 (135.82 to 1456.5) | 5.25 (4.19 to 6.32) |
| Suriname | 140 (97 to 205) | 146.58 (102.68 to 212.46) | 1807 (1164 to 2749) | 1231.88 (790.02 to 1879.56) | 5.86 (5.01 to 6.73) |
| Sweden | 232 (132 to 363) | 11.31 (6.37 to 17.73) | 1434 (710 to 2227) | 60.35 (29.98 to 93.57) | 5.05 (4.77 to 5.33) |
| Switzerland | 1150 (654 to 1825) | 64.42 (36.49 to 102.57) | 2868 (1419 to 4781) | 126.13 (62.58 to 209.72) | 1.29 (0.82 to 1.76) |
| Syrian Arab Republic | 26 (19 to 35) | 1.01 (0.74 to 1.37) | 96 (55 to 166) | 2.69 (1.5 to 4.72) | 2.76 (2.39 to 3.12) |
| Taiwan (Province of China) | 51 (31 to 70) | 0.94 (0.58 to 1.27) | 377 (163 to 769) | 6.14 (2.61 to 12.47) | 6.06 (5.17 to 6.96) |
| Tajikistan | 204 (127 to 319) | 19.61 (12.6 to 29.95) | 2776 (1788 to 3983) | 115.59 (74.05 to 165.97) | 4.34 (3.63 to 5.04) |
| Thailand | 11383 (6518 to 17954) | 67.93 (39.19 to 106.89) | 157038 (95847 to 238807) | 871.52 (518.19 to 1355.85) | 4.54 (3.14 to 5.97) |
| Timor-Leste | 56 (41 to 74) | 29.02 (21.12 to 38.39) | 866 (691 to 1062) | 283.81 (227.55 to 347.34) | 4.43 (3.05 to 5.84) |
| Togo | 8646 (4775 to 14436) | 1025.46 (566.82 to 1705.93) | 56498 (42221 to 72606) | 2818.66 (2122.02 to 3596.82) | 0.89 (-0.33 to 2.12) |
| Tokelau | 0 (0 to 0) | 3.56 (2.13 to 4.8) | 0 (0 to 0) | 116.41 (80.99 to 152.91) | 11.52 (11.21 to 11.84) |
| Tonga | 2 (1 to 2) | 7.96 (4.69 to 10.79) | 69 (49 to 88) | 287.92 (204.6 to 365.82) | 12.08 (11.94 to 12.23) |
| Trinidad and Tobago | 511 (282 to 881) | 162.06 (89.88 to 278.75) | 3750 (2983 to 5012) | 1045 (826.54 to 1410.32) | 4.87 (4.04 to 5.71) |
| Tunisia | 6 (2 to 14) | 0.3 (0.08 to 0.71) | 619 (407 to 820) | 18.72 (12.14 to 25.1) | 13.72 (12.46 to 14.99) |
| Turkey | 57 (0 to 101) | 0.41 (0 to 0.72) | 1459 (948 to 2296) | 6.84 (4.39 to 10.85) | 9.41 (8.78 to 10.05) |
| Turkmenistan | 222 (159 to 290) | 24.26 (17.69 to 31.37) | 589 (413 to 912) | 47.26 (33.2 to 72.92) | 1.83 (1.63 to 2.04) |
| Tuvalu | 0 (0 to 0) | 3.61 (2.11 to 5.08) | 3 (2 to 4) | 119.5 (83.78 to 156.1) | 11.29 (10.94 to 11.63) |
| Uganda | 498020 (398622 to 606440) | 13336.77 (10634.05 to 16373.72) | 762694 (593567 to 938739) | 8734.61 (6918.52 to 10590.82) | -1.37 (-1.64 to -1.09) |
| Ukraine | 3542 (2045 to 5898) | 27.1 (15.56 to 45.31) | 96344 (55751 to 163600) | 806.73 (454.19 to 1403.29) | 10.57 (9.29 to 11.86) |
| United Arab Emirates | 7 (5 to 11) | 1.88 (1.24 to 2.96) | 390 (287 to 496) | 19.21 (13.55 to 25.3) | 7.26 (6.23 to 8.3) |
| United Kingdom | 2444 (1415 to 3841) | 16.97 (9.8 to 26.71) | 26586 (15568 to 38257) | 157.73 (92.9 to 226.5) | 7.41 (6.15 to 8.69) |
| United Republic of Tanzania | 392126 (280947 to 524029) | 6605.64 (4720.1 to 8861.19) | 1154441 (819749 to 1729129) | 8754.49 (6378.75 to 12696.89) | -0.44 (-0.85 to -0.02) |
| United States of America | 139338 (84426 to 209057) | 198.32 (119.61 to 298.4) | 247056 (125074 to 387936) | 305.21 (154.78 to 478.33) | 1.16 (1.01 to 1.3) |
| United States Virgin Islands | 18 (12 to 27) | 62.47 (39.89 to 94.36) | 32 (22 to 47) | 171.62 (118.23 to 259.64) | 2.22 (1.69 to 2.76) |
| Uruguay | 229 (107 to 424) | 30.66 (14.28 to 56.61) | 2509 (1212 to 4062) | 297.3 (142.21 to 482.08) | 6.65 (6.07 to 7.23) |
| Uzbekistan | 452 (280 to 644) | 9.5 (6.02 to 13.39) | 9205 (4365 to 16552) | 97.38 (46.4 to 174.75) | 6.77 (6.38 to 7.17) |
| Vanuatu | 3 (2 to 3) | 7.38 (4.49 to 9.95) | 171 (117 to 221) | 225.16 (155.52 to 289.71) | 11.11 (10.83 to 11.39) |
| Venezuela (Bolivarian Republic of) | 985 (701 to 1370) | 21.12 (15.08 to 29.35) | 18434 (12582 to 26801) | 257.61 (172.62 to 378.14) | 7.82 (6.66 to 9) |
| Viet Nam | 2868 (2186 to 3540) | 15.65 (12 to 19.22) | 42230 (27898 to 63136) | 156.58 (102.95 to 234.68) | 6.57 (5.67 to 7.48) |
| Yemen | 75 (33 to 143) | 2.83 (1.22 to 5.38) | 3040 (1842 to 4433) | 38.59 (23.79 to 55.7) | 9.09 (8.66 to 9.52) |
| Zambia | 195374 (130657 to 267940) | 10476.76 (6934.87 to 14463.99) | 632831 (529905 to 733991) | 15197.97 (12848.07 to 17449.87) | 0.33 (-0.08 to 0.75) |
| Zimbabwe | 352064 (277199 to 430624) | 14586.12 (11407.39 to 17964.51) | 591958 (507395 to 670755) | 16438.87 (14163.62 to 18529.41) | -0.89 (-1.45 to -0.33) |

**Supplementary Table S3**

The mortality of HIV/AIDS cases and rates among women of childbearing age across 204 countries in 1990 and 2021, and the trends from 1990 to 2021

| location | Num_1990 | ASR_1990 | Num_2021 | ASR_2021 | EAPC_CI |
| --- | --- | --- | --- | --- | --- |
| Afghanistan | 3 (0 to 7) | 0.17 (0.03 to 0.38) | 28 (7 to 62) | 0.46 (0.12 to 1.01) | 4.24 (3.39 to 5.1) |
| Albania | 0 (0 to 0) | 0.02 (0.02 to 0.02) | 0 (0 to 0) | 0.03 (0.03 to 0.03) | -0.83 (-2.01 to 0.37) |
| Algeria | 6 (3 to 11) | 0.13 (0.07 to 0.21) | 67 (30 to 136) | 0.57 (0.26 to 1.15) | 5.68 (4.25 to 7.12) |
| American Samoa | 0 (0 to 0) | 0.92 (0.92 to 0.93) | 0 (0 to 0) | 3.4 (3.39 to 3.41) | 4.73 (3.05 to 6.44) |
| Andorra | 0 (0 to 0) | 0.11 (0.05 to 0.22) | 0 (0 to 0) | 0.05 (0.02 to 0.12) | -5.03 (-5.96 to -4.08) |
| Angola | 93 (42 to 192) | 4.35 (1.96 to 9.01) | 9264 (5392 to 15195) | 138.58 (81.73 to 224.01) | 9.03 (6.92 to 11.18) |
| Antigua and Barbuda | 1 (1 to 1) | 8.21 (8.16 to 8.25) | 1 (1 to 1) | 5.61 (5.6 to 5.63) | -1.44 (-2.12 to -0.75) |
| Argentina | 99 (99 to 99) | 1.26 (1.25 to 1.26) | 386 (384 to 388) | 3.11 (3.09 to 3.13) | 1.44 (0.24 to 2.67) |
| Armenia | 1 (1 to 1) | 0.12 (0.12 to 0.12) | 3 (3 to 3) | 0.3 (0.29 to 0.3) | 2.87 (2.52 to 3.22) |
| Australia | 6 (6 to 6) | 0.14 (0.14 to 0.14) | 7 (7 to 7) | 0.1 (0.1 to 0.1) | -2.86 (-3.84 to -1.88) |
| Austria | 10 (10 to 10) | 0.48 (0.48 to 0.48) | 5 (5 to 5) | 0.25 (0.25 to 0.25) | -4.14 (-4.95 to -3.33) |
| Azerbaijan | 5 (5 to 5) | 0.3 (0.3 to 0.3) | 8 (8 to 8) | 0.25 (0.25 to 0.26) | -1.44 (-2.07 to -0.79) |
| Bahamas | 17 (17 to 17) | 24.99 (24.63 to 25.36) | 34 (33 to 34) | 30.25 (29.7 to 30.79) | -2.09 (-3.33 to -0.83) |
| Bahrain | 0 (0 to 0) | 0.29 (0.29 to 0.29) | 4 (4 to 4) | 1.23 (1.23 to 1.24) | 7.02 (5.55 to 8.51) |
| Bangladesh | 0 (0 to 0) | 0 (0 to 0) | 153 (74 to 274) | 0.34 (0.17 to 0.6) | 23.12 (20.37 to 25.92) |
| Barbados | 5 (5 to 5) | 7.54 (7.5 to 7.57) | 5 (5 to 5) | 6.98 (6.95 to 7) | -1.05 (-1.97 to -0.12) |
| Belarus | 21 (21 to 21) | 0.79 (0.79 to 0.79) | 111 (111 to 111) | 4.41 (4.4 to 4.42) | 6.53 (6.01 to 7.05) |
| Belgium | 21 (21 to 21) | 0.82 (0.82 to 0.82) | 10 (10 to 10) | 0.39 (0.39 to 0.39) | -4.11 (-4.83 to -3.39) |
| Belize | 5 (5 to 5) | 13.63 (13.52 to 13.74) | 23 (23 to 23) | 20.09 (19.86 to 20.32) | 1.01 (-0.21 to 2.24) |
| Benin | 10 (2 to 28) | 0.91 (0.18 to 2.61) | 644 (314 to 1110) | 23.12 (11.31 to 39.78) | 2.79 (-1.09 to 6.83) |
| Bermuda | 2 (2 to 2) | 9.25 (9.19 to 9.3) | 1 (1 to 1) | 5.06 (5.04 to 5.07) | -2.22 (-2.9 to -1.53) |
| Bhutan | 0 (0 to 1) | 0.23 (0.05 to 0.6) | 2 (1 to 5) | 1.1 (0.37 to 2.62) | 4.63 (2.16 to 7.17) |
| Bolivia (Plurinational State of) | 1 (0 to 6) | 0.09 (0.01 to 0.41) | 83 (31 to 177) | 2.73 (1.01 to 5.84) | 10.4 (5.57 to 15.46) |
| Bosnia and Herzegovina | 1 (1 to 1) | 0.11 (0.11 to 0.11) | 1 (1 to 1) | 0.16 (0.16 to 0.16) | 1.76 (1.21 to 2.31) |
| Botswana | 446 (211 to 843) | 145.36 (66.24 to 278.76) | 2022 (969 to 3702) | 298.36 (142.67 to 547.78) | -1.77 (-4.16 to 0.67) |
| Brazil | 1169 (1168 to 1170) | 3.12 (3.12 to 3.13) | 3188 (3181 to 3194) | 5.04 (5.03 to 5.05) | -0.29 (-1.18 to 0.61) |
| Brunei Darussalam | 0 (0 to 0) | 0.1 (0.1 to 0.1) | 0 (0 to 0) | 0.36 (0.35 to 0.36) | 3.2 (2.64 to 3.76) |
| Bulgaria | 7 (7 to 7) | 0.33 (0.33 to 0.33) | 7 (7 to 7) | 0.49 (0.49 to 0.49) | 0.62 (-0.4 to 1.64) |
| Burkina Faso | 3738 (1950 to 6377) | 194.99 (102.1 to 329.9) | 751 (376 to 1279) | 16.23 (8.12 to 27.62) | -9.59 (-10.68 to -8.48) |
| Burundi | 1751 (758 to 4046) | 149.25 (64.22 to 350.16) | 644 (296 to 1186) | 24.78 (11.31 to 46.01) | -8.13 (-10.12 to -6.1) |
| Cabo Verde | 8 (3 to 20) | 11.79 (3.67 to 28.54) | 14 (4 to 42) | 9.49 (2.46 to 29.03) | -3.15 (-5 to -1.26) |
| Cambodia | 0 (0 to 0) | 0 (0 to 0) | 302 (105 to 621) | 6.94 (2.38 to 14.3) | 16.28 (6.66 to 26.78) |
| Cameroon | 562 (273 to 1043) | 25.1 (11.97 to 46.85) | 7546 (4453 to 11697) | 113.27 (67.1 to 174.98) | 2.9 (0.37 to 5.49) |
| Canada | 43 (43 to 43) | 0.55 (0.55 to 0.55) | 25 (25 to 25) | 0.27 (0.27 to 0.27) | -4.05 (-4.85 to -3.24) |
| Central African Republic | 846 (317 to 1714) | 134.86 (49.38 to 276.56) | 1946 (1083 to 3300) | 163 (92.8 to 271.24) | -2.28 (-4.07 to -0.46) |
| Chad | 279 (129 to 571) | 22.61 (10.4 to 46.28) | 1132 (525 to 2102) | 35.38 (16.27 to 65.78) | -0.89 (-2.81 to 1.07) |
| Chile | 17 (17 to 17) | 0.49 (0.49 to 0.49) | 67 (67 to 68) | 1.34 (1.33 to 1.34) | 2.45 (1.67 to 3.23) |
| China | 281 (17 to 519) | 0.1 (0.01 to 0.18) | 3574 (2563 to 4778) | 0.97 (0.7 to 1.28) | 6.3 (5.13 to 7.48) |
| Colombia | 97 (97 to 97) | 1.23 (1.22 to 1.23) | 460 (458 to 461) | 3.45 (3.44 to 3.46) | 3.81 (2.74 to 4.9) |
| Comoros | 0 (0 to 0) | 0.04 (0 to 0.16) | 0 (0 to 1) | 0.21 (0.09 to 0.41) | 5.05 (3.09 to 7.04) |
| Congo | 728 (329 to 1361) | 140.86 (62.75 to 264.95) | 1942 (1149 to 3152) | 143.66 (85.75 to 231.73) | -2.27 (-3.41 to -1.11) |
| Cook Islands | 0 (0 to 0) | 0.09 (0.02 to 0.21) | 0 (0 to 0) | 2.74 (0.96 to 5.24) | 9.75 (8.63 to 10.89) |
| Costa Rica | 11 (11 to 11) | 1.63 (1.62 to 1.63) | 34 (34 to 34) | 2.48 (2.48 to 2.48) | 1.62 (1.33 to 1.92) |
| Coted'Ivoire | 5662 (2714 to 10754) | 227.09 (107.4 to 431.06) | 4283 (2251 to 7209) | 73.32 (38.43 to 123.81) | -5.46 (-7.09 to -3.8) |
| Croatia | 1 (1 to 1) | 0.1 (0.1 to 0.1) | 1 (1 to 1) | 0.1 (0.1 to 0.1) | -0.87 (-1.74 to -0.01) |
| Cuba | 25 (25 to 25) | 0.84 (0.84 to 0.84) | 25 (25 to 25) | 0.92 (0.92 to 0.92) | 0.19 (-0.4 to 0.78) |
| Cyprus | 0 (0 to 0) | 0.17 (0.17 to 0.17) | 1 (1 to 1) | 0.3 (0.3 to 0.3) | 1.13 (-0.08 to 2.35) |
| Czechia | 1 (1 to 1) | 0.06 (0.06 to 0.06) | 1 (1 to 1) | 0.06 (0.06 to 0.06) | -0.42 (-1.25 to 0.43) |
| Democratic People's Republic of Korea | 1 (0 to 2) | 0.01 (0 to 0.05) | 88 (15 to 334) | 1.28 (0.22 to 4.84) | 13.31 (10.28 to 16.43) |
| Democratic Republic of the Congo | 7121 (3977 to 12091) | 93.03 (52.49 to 156.28) | 2990 (1499 to 5519) | 15.9 (7.98 to 29.24) | -5.54 (-7.32 to -3.72) |
| Denmark | 6 (6 to 6) | 0.42 (0.42 to 0.42) | 3 (3 to 3) | 0.2 (0.2 to 0.21) | -4.54 (-5.35 to -3.74) |
| Djibouti | 0 (0 to 1) | 0.23 (0.02 to 0.88) | 374 (216 to 614) | 116.61 (67.6 to 191.01) | 13.78 (8.3 to 19.54) |
| Dominica | 1 (1 to 1) | 5.53 (5.51 to 5.56) | 1 (1 to 1) | 5.57 (5.55 to 5.59) | -0.46 (-1.41 to 0.5) |
| Dominican Republic | 99 (31 to 246) | 5.42 (1.64 to 13.63) | 201 (52 to 466) | 7.07 (1.82 to 16.43) | -2.4 (-4.68 to -0.06) |
| Ecuador | 21 (21 to 21) | 0.92 (0.92 to 0.92) | 191 (190 to 192) | 4.07 (4.05 to 4.09) | 5.79 (4.44 to 7.16) |
| Egypt | 24 (24 to 24) | 0.2 (0.2 to 0.2) | 141 (141 to 141) | 0.57 (0.56 to 0.57) | 2.98 (2.32 to 3.64) |
| El Salvador | 27 (27 to 27) | 2.27 (2.26 to 2.27) | 74 (74 to 75) | 4.23 (4.22 to 4.25) | 2.15 (0.68 to 3.65) |
| Equatorial Guinea | 10 (5 to 23) | 11.06 (5.02 to 24.56) | 882 (411 to 1709) | 267.45 (125.02 to 512.38) | 8.63 (6.18 to 11.14) |
| Eritrea | 209 (88 to 457) | 28.75 (12.07 to 62.89) | 383 (230 to 603) | 26.03 (15.89 to 40.65) | -2.96 (-4.96 to -0.93) |
| Estonia | 2 (2 to 2) | 0.47 (0.47 to 0.47) | 9 (9 to 9) | 2.92 (2.91 to 2.93) | 6.64 (6.36 to 6.92) |
| Eswatini | 9 (3 to 25) | 4.57 (1.43 to 12.58) | 1463 (639 to 2866) | 486.17 (213.16 to 947.87) | 8.81 (3.93 to 13.91) |
| Ethiopia | 3598 (1871 to 6720) | 34.13 (17.65 to 63.41) | 6867 (3786 to 11156) | 30.01 (16.61 to 48.5) | -3.72 (-5.73 to -1.66) |
| Fiji | 5 (5 to 5) | 2.53 (2.53 to 2.54) | 10 (10 to 10) | 4.4 (4.39 to 4.42) | 1.22 (-0.15 to 2.61) |
| Finland | 3 (3 to 3) | 0.19 (0.19 to 0.19) | 1 (1 to 1) | 0.11 (0.11 to 0.12) | -3.4 (-4.07 to -2.73) |
| France | 328 (327 to 328) | 2.2 (2.19 to 2.21) | 70 (70 to 70) | 0.45 (0.45 to 0.45) | -7.67 (-8.6 to -6.74) |
| Gabon | 70 (35 to 132) | 34.88 (17.24 to 65.37) | 505 (255 to 864) | 115.51 (58.51 to 196.68) | 1.22 (-0.83 to 3.31) |
| Gambia | 9 (4 to 17) | 4.14 (1.9 to 8.26) | 437 (216 to 775) | 84.31 (42.47 to 146.98) | 7.56 (4.94 to 10.24) |
| Georgia | 3 (3 to 3) | 0.2 (0.19 to 0.2) | 6 (6 to 6) | 0.66 (0.66 to 0.67) | 4.17 (3.93 to 4.42) |
| Germany | 157 (157 to 158) | 0.76 (0.76 to 0.76) | 52 (52 to 52) | 0.27 (0.27 to 0.27) | -4.97 (-5.73 to -4.19) |
| Ghana | 1106 (566 to 1934) | 33.93 (17.37 to 59.08) | 7016 (4270 to 10868) | 83.68 (51.74 to 127.92) | 0.84 (-0.92 to 2.63) |
| Greece | 4 (4 to 4) | 0.18 (0.18 to 0.18) | 3 (3 to 3) | 0.12 (0.12 to 0.12) | -2.85 (-3.57 to -2.12) |
| Greenland | 0 (0 to 0) | 2.57 (2.57 to 2.58) | 0 (0 to 0) | 1.87 (1.86 to 1.88) | -2.83 (-3.95 to -1.7) |
| Grenada | 1 (1 to 1) | 6.23 (6.2 to 6.25) | 1 (1 to 1) | 4.27 (4.26 to 4.29) | -1.25 (-1.91 to -0.58) |
| Guam | 0 (0 to 0) | 0.95 (0.95 to 0.95) | 2 (2 to 2) | 6.01 (5.98 to 6.04) | 6.03 (4.7 to 7.37) |
| Guatemala | 77 (77 to 77) | 4.73 (4.72 to 4.73) | 143 (143 to 144) | 3.5 (3.48 to 3.52) | -1.08 (-1.86 to -0.29) |
| Guinea | 136 (67 to 272) | 10.33 (5.08 to 20.57) | 1250 (689 to 2057) | 43.81 (24.39 to 71.46) | 2.45 (0.24 to 4.7) |
| Guinea-Bissau | 16 (7 to 32) | 7.62 (3.36 to 14.87) | 447 (172 to 915) | 94.4 (35.66 to 193.69) | 6.06 (3.79 to 8.38) |
| Guyana | 35 (34 to 35) | 18.78 (18.62 to 18.93) | 58 (57 to 59) | 29.45 (28.92 to 29.97) | 0.61 (-0.95 to 2.19) |
| Haiti | 2157 (1026 to 3920) | 147.73 (70.02 to 268.34) | 1767 (920 to 2939) | 52.24 (27.54 to 86.43) | -4.95 (-6.05 to -3.84) |
| Honduras | 71 (71 to 72) | 7.33 (7.31 to 7.35) | 65 (65 to 65) | 2.39 (2.38 to 2.4) | -3.17 (-3.27 to -3.08) |
| Hungary | 13 (13 to 13) | 0.48 (0.47 to 0.48) | 8 (8 to 8) | 0.34 (0.34 to 0.34) | -2.21 (-3.04 to -1.38) |
| Iceland | 0 (0 to 0) | 0.39 (0.39 to 0.39) | 0 (0 to 0) | 0.24 (0.24 to 0.24) | -3.15 (-3.82 to -2.47) |
| India | 49 (13 to 120) | 0.03 (0.01 to 0.06) | 14097 (9187 to 20151) | 3.81 (2.49 to 5.45) | 10.95 (5.46 to 16.71) |
| Indonesia | 103 (103 to 103) | 0.24 (0.24 to 0.24) | 846 (805 to 909) | 1.08 (1.03 to 1.16) | 5.84 (4.84 to 6.85) |
| Iran (Islamic Republic of) | 4 (4 to 4) | 0.04 (0.04 to 0.04) | 97 (97 to 97) | 0.38 (0.37 to 0.38) | 9.34 (8.31 to 10.39) |
| Iraq | 2 (2 to 2) | 0.07 (0.07 to 0.07) | 44 (44 to 44) | 0.45 (0.45 to 0.45) | 7.15 (6.52 to 7.78) |
| Ireland | 2 (2 to 2) | 0.28 (0.28 to 0.28) | 2 (2 to 2) | 0.16 (0.16 to 0.16) | -3.74 (-4.45 to -3.03) |
| Israel | 7 (7 to 7) | 0.57 (0.57 to 0.57) | 7 (7 to 7) | 0.3 (0.3 to 0.3) | -3.8 (-4.48 to -3.12) |
| Italy | 279 (278 to 279) | 1.94 (1.94 to 1.95) | 71 (71 to 71) | 0.49 (0.49 to 0.49) | -7.32 (-8.39 to -6.25) |
| Jamaica | 57 (57 to 58) | 10.75 (10.68 to 10.83) | 136 (134 to 137) | 17.47 (17.29 to 17.66) | 0.83 (-0.21 to 1.88) |
| Japan | 4 (4 to 4) | 0.01 (0.01 to 0.01) | 8 (8 to 8) | 0.03 (0.03 to 0.03) | 1.76 (0.76 to 2.76) |
| Jordan | 0 (0 to 0) | 0.07 (0.07 to 0.07) | 11 (11 to 11) | 0.39 (0.39 to 0.39) | 5.98 (5.27 to 6.7) |
| Kazakhstan | 20 (20 to 20) | 0.49 (0.49 to 0.49) | 63 (63 to 63) | 1.22 (1.22 to 1.22) | 2.11 (1.28 to 2.94) |
| Kenya | 7525 (4064 to 12850) | 162.41 (85.78 to 277.71) | 11052 (6519 to 17482) | 95.54 (56.32 to 153.16) | -4.27 (-6.36 to -2.12) |
| Kiribati | 0 (0 to 0) | 2.76 (2.75 to 2.76) | 1 (1 to 1) | 3.46 (3.44 to 3.47) | -0.11 (-1.25 to 1.04) |
| Kuwait | 0 (0 to 0) | 0.13 (0.13 to 0.13) | 2 (2 to 2) | 0.1 (0.1 to 0.1) | -0.08 (-1.44 to 1.3) |
| Kyrgyzstan | 10 (10 to 10) | 1.04 (1.04 to 1.04) | 49 (48 to 49) | 2.8 (2.79 to 2.81) | 2.91 (2.57 to 3.25) |
| Lao People's Democratic Republic | 0 (0 to 0) | 0.01 (0 to 0.01) | 34 (12 to 76) | 1.8 (0.63 to 4.01) | 20.96 (15.39 to 26.81) |
| Latvia | 11 (11 to 11) | 1.65 (1.64 to 1.66) | 52 (51 to 52) | 11.46 (11.39 to 11.53) | 6.64 (6.31 to 6.98) |
| Lebanon | 1 (1 to 2) | 0.21 (0.11 to 0.34) | 21 (12 to 33) | 1.29 (0.75 to 2.03) | 6.04 (5.58 to 6.51) |
| Lesotho | 210 (101 to 381) | 57.9 (27.38 to 105.85) | 2573 (1372 to 4269) | 563.35 (299.99 to 936.66) | 4.2 (1.46 to 7.01) |
| Liberia | 50 (20 to 123) | 9.37 (3.67 to 23.23) | 514 (279 to 855) | 40.87 (22.22 to 67.94) | 3.22 (0.71 to 5.79) |
| Libya | 2 (0 to 8) | 0.23 (0.01 to 1.1) | 26 (0 to 245) | 1.24 (0.01 to 11.59) | 5.73 (4.68 to 6.79) |
| Lithuania | 17 (17 to 17) | 1.76 (1.75 to 1.76) | 65 (65 to 65) | 10.39 (10.33 to 10.44) | 6.04 (5.73 to 6.35) |
| Luxembourg | 1 (1 to 1) | 0.56 (0.56 to 0.56) | 1 (1 to 1) | 0.3 (0.3 to 0.3) | -4.05 (-4.76 to -3.33) |
| Madagascar | 0 (0 to 1) | 0.01 (0 to 0.04) | 1297 (664 to 2272) | 20.79 (10.76 to 35.95) | 21.7 (16.47 to 27.17) |
| Malawi | 5476 (2809 to 9492) | 267 (134.45 to 463.88) | 7272 (3732 to 12486) | 175.34 (89.86 to 302.11) | -4.87 (-6.83 to -2.88) |
| Malaysia | 44 (43 to 44) | 1.09 (1.09 to 1.09) | 244 (243 to 245) | 2.92 (2.9 to 2.93) | 4.24 (3.22 to 5.26) |
| Maldives | 0 (0 to 0) | 0.15 (0.15 to 0.15) | 1 (1 to 1) | 0.93 (0.93 to 0.93) | 6.86 (5.79 to 7.94) |
| Mali | 191 (84 to 405) | 10.49 (4.58 to 22.35) | 1696 (908 to 2953) | 38.29 (20.92 to 65.47) | 1.54 (-0.52 to 3.65) |
| Malta | 0 (0 to 0) | 0.35 (0.35 to 0.35) | 0 (0 to 0) | 0.22 (0.22 to 0.22) | -2.86 (-3.44 to -2.28) |
| Marshall Islands | 0 (0 to 0) | 0.21 (0.04 to 0.47) | 1 (0 to 2) | 7.79 (3.48 to 13.86) | 10.98 (10.19 to 11.77) |
| Mauritania | 0 (0 to 0) | 0.06 (0.02 to 0.1) | 1 (0 to 1) | 0.05 (0.02 to 0.1) | -1.96 (-3.42 to -0.47) |
| Mauritius | 1 (1 to 1) | 0.27 (0.27 to 0.27) | 11 (11 to 11) | 3.38 (3.36 to 3.39) | 17.21 (14.22 to 20.28) |
| Mexico | 341 (341 to 341) | 1.81 (1.81 to 1.81) | 845 (843 to 846) | 2.36 (2.36 to 2.37) | 0.51 (0.12 to 0.91) |
| Micronesia (Federated States of) | 0 (0 to 0) | 0.21 (0.04 to 0.48) | 2 (1 to 4) | 8.17 (3.61 to 14.61) | 11.11 (10.35 to 11.89) |
| Monaco | 0 (0 to 0) | 0.25 (0.09 to 0.52) | 0 (0 to 0) | 0.12 (0.05 to 0.27) | -3.59 (-4.27 to -2.9) |
| Mongolia | 0 (0 to 0) | 0.12 (0.12 to 0.12) | 2 (2 to 2) | 0.25 (0.25 to 0.25) | 1.46 (0.71 to 2.2) |
| Montenegro | 0 (0 to 0) | 0.14 (0.14 to 0.14) | 0 (0 to 0) | 0.15 (0.15 to 0.15) | -1.01 (-2.01 to 0.01) |
| Morocco | 27 (10 to 58) | 0.46 (0.18 to 1) | 82 (31 to 176) | 0.83 (0.32 to 1.78) | 2.34 (0.19 to 4.54) |
| Mozambique | 587 (370 to 954) | 19.65 (12.46 to 31.97) | 22254 (19483 to 25795) | 322.18 (284.2 to 371.32) | 7.13 (4.48 to 9.84) |
| Myanmar | 9 (2 to 24) | 0.09 (0.02 to 0.23) | 1355 (557 to 2448) | 9.03 (3.72 to 16.29) | 9.56 (4.86 to 14.48) |
| Namibia | 109 (51 to 203) | 35.33 (16.19 to 66.2) | 1234 (641 to 2161) | 199.27 (103.54 to 348.84) | 1.77 (-1.2 to 4.83) |
| Nauru | 0 (0 to 0) | 0.09 (0.02 to 0.2) | 0 (0 to 0) | 2.8 (0.96 to 5.53) | 9.78 (8.65 to 10.93) |
| Nepal | 0 to 0 | 0 (0 to 0) | 88 (32 to 195) | 1.03 (0.37 to 2.32) | 19.77 (11.41 to 28.76) |
| Netherlands | 17 (17 to 17) | 0.41 (0.41 to 0.41) | 8 (8 to 8) | 0.21 (0.21 to 0.21) | -4.46 (-5.25 to -3.66) |
| New Zealand | 2 (2 to 2) | 0.17 (0.17 to 0.17) | 1 (1 to 1) | 0.08 (0.08 to 0.08) | -2.53 (-3.82 to -1.22) |
| Nicaragua | 9 (9 to 9) | 1.18 (1.18 to 1.18) | 39 (39 to 39) | 2.17 (2.17 to 2.18) | 2.7 (2.29 to 3.12) |
| Niger | 100 (45 to 215) | 6.26 (2.83 to 13.52) | 300 (132 to 585) | 7.26 (3.19 to 14.22) | -1.46 (-3.93 to 1.07) |
| Nigeria | 3154 (1725 to 5525) | 17.51 (9.53 to 30.55) | 30179 (19793 to 43978) | 63.5 (42.17 to 91.35) | 0.92 (-1 to 2.89) |
| Niue | 0 (0 to 0) | 0.09 (0.02 to 0.21) | 0 (0 to 0) | 2.29 (0.71 to 4.7) | 8.8 (7.66 to 9.95) |
| North Macedonia | 0 (0 to 0) | 0.03 (0.03 to 0.03) | 0 (0 to 0) | 0.04 (0.04 to 0.04) | -0.6 (-1.65 to 0.45) |
| Northern Mariana Islands | 0 (0 to 0) | 1.23 (1.22 to 1.23) | 0 (0 to 0) | 3.72 (3.7 to 3.73) | 3.49 (2.03 to 4.97) |
| Norway | 1 (1 to 1) | 0.11 (0.11 to 0.11) | 3 (3 to 3) | 0.21 (0.21 to 0.21) | -1.4 (-2.91 to 0.13) |
| Oman | 1 (1 to 1) | 0.26 (0.25 to 0.26) | 14 (14 to 14) | 1.31 (1.3 to 1.31) | 6.05 (5.41 to 6.69) |
| Pakistan | 0 (0 to 0) | 0 (0 to 0) | 2286 (12 to 13704) | 3.99 (0.02 to 24.16) | 41.48 (38.43 to 44.61) |
| Palau | 0 (0 to 0) | 0.09 (0.02 to 0.2) | 0 (0 to 0) | 2.26 (0.74 to 4.49) | 9.14 (8.05 to 10.24) |
| Palestine | 0 (0 to 0) | 0.02 (0.02 to 0.02) | 3 (3 to 3) | 0.23 (0.23 to 0.23) | 9.35 (8.01 to 10.7) |
| Panama | 26 (26 to 26) | 4.6 (4.58 to 4.62) | 89 (89 to 90) | 8.35 (8.31 to 8.39) | 1.52 (0.65 to 2.39) |
| Papua New Guinea | 0 (0 to 1) | 0.04 (0.01 to 0.1) | 273 (121 to 524) | 11.09 (4.91 to 21.27) | 14.26 (8.51 to 20.31) |
| Paraguay | 10 (10 to 10) | 1.17 (1.17 to 1.17) | 63 (63 to 64) | 3.44 (3.41 to 3.46) | 2.79 (1.92 to 3.66) |
| Peru | 57 (57 to 57) | 1.19 (1.19 to 1.19) | 267 (266 to 269) | 2.72 (2.71 to 2.74) | 1.96 (0.47 to 3.48) |
| Philippines | 3 (3 to 3) | 0.02 (0.02 to 0.02) | 968 (941 to 1005) | 3.41 (3.31 to 3.54) | 22.14 (18.33 to 26.08) |
| Poland | 2 (2 to 2) | 0.02 (0.02 to 0.02) | 20 (20 to 20) | 0.2 (0.2 to 0.2) | 9.13 (6.05 to 12.29) |
| Portugal | 53 (52 to 53) | 2.09 (2.08 to 2.1) | 38 (38 to 39) | 1.43 (1.43 to 1.44) | -4.12 (-5.28 to -2.94) |
| Puerto Rico | 130 (130 to 131) | 13.7 (13.64 to 13.76) | 31 (31 to 31) | 3.85 (3.84 to 3.85) | -6.01 (-6.76 to -5.25) |
| Qatar | 0 (0 to 0) | 0.45 (0.44 to 0.45) | 2 (2 to 2) | 0.38 (0.38 to 0.38) | -0.91 (-1.94 to 0.13) |
| Republic of Korea | 2 (2 to 2) | 0.02 (0.02 to 0.02) | 4 (4 to 4) | 0.03 (0.03 to 0.03) | 1.26 (0.83 to 1.68) |
| Republic of Moldova | 8 (8 to 8) | 0.7 (0.7 to 0.71) | 48 (47 to 48) | 4.58 (4.57 to 4.59) | 6.59 (6.26 to 6.91) |
| Romania | 30 (30 to 30) | 0.53 (0.53 to 0.54) | 36 (35 to 36) | 0.92 (0.91 to 0.92) | 1.38 (0.39 to 2.39) |
| Russian Federation | 481 (480 to 481) | 1.23 (1.23 to 1.23) | 5220 (5209 to 5231) | 13.09 (13.06 to 13.12) | 9.47 (8.99 to 9.95) |
| Rwanda | 866 (353 to 2220) | 58.19 (23.41 to 150.03) | 1761 (916 to 2994) | 56.18 (29.13 to 95.92) | -3.89 (-5.87 to -1.88) |
| Saint Kitts and Nevis | 0 (0 to 0) | 1.83 (0.56 to 4.48) | 3 (2 to 4) | 16.26 (8.85 to 25.77) | 5.44 (3.47 to 7.44) |
| Saint Lucia | 2 (2 to 2) | 5.82 (5.79 to 5.84) | 2 (2 to 2) | 3.7 (3.69 to 3.71) | -1.59 (-2.26 to -0.91) |
| Saint Vincent and the Grenadines | 4 (4 to 4) | 17.78 (17.6 to 17.96) | 5 (5 to 5) | 15.96 (15.81 to 16.1) | -0.51 (-1.54 to 0.53) |
| Samoa | 0 (0 to 0) | 0.21 (0.04 to 0.49) | 4 (2 to 6) | 7.86 (3.45 to 14.06) | 10.98 (10.24 to 11.72) |
| San Marino | 0 (0 to 0) | 0.25 (0.09 to 0.52) | 0 (0 to 0) | 0.1 (0.04 to 0.22) | -4.4 (-5.03 to -3.76) |
| Sao Tome and Principe | 0 (0 to 0) | 0.04 (0.02 to 0.09) | 0 (0 to 0) | 0.04 (0.03 to 0.07) | -3.81 (-5.64 to -1.94) |
| Saudi Arabia | 26 (26 to 26) | 0.94 (0.94 to 0.95) | 311 (310 to 312) | 2.75 (2.74 to 2.76) | 3.95 (2.74 to 5.16) |
| Senegal | 110 (55 to 205) | 7.11 (3.57 to 13.13) | 516 (253 to 901) | 15.3 (7.52 to 26.61) | 0.35 (-1.81 to 2.56) |
| Serbia | 4 (4 to 4) | 0.18 (0.18 to 0.18) | 3 (3 to 3) | 0.16 (0.16 to 0.16) | -1.76 (-2.73 to -0.78) |
| Seychelles | 0 (0 to 0) | 0.81 (0.81 to 0.81) | 2 (2 to 2) | 5.87 (5.81 to 5.92) | 8.48 (7.07 to 9.92) |
| Sierra Leone | 51 (22 to 117) | 5.32 (2.29 to 12.24) | 704 (348 to 1200) | 35.63 (17.53 to 60.68) | 4.34 (1.76 to 6.98) |
| Singapore | 0 (0 to 0) | 0.04 (0.04 to 0.04) | 1 (1 to 1) | 0.05 (0.05 to 0.05) | -0.46 (-1.2 to 0.29) |
| Slovakia | 0 (0 to 0) | 0.03 (0.03 to 0.03) | 0 (0 to 0) | 0.03 (0.03 to 0.03) | -0.7 (-1.65 to 0.26) |
| Slovenia | 0 (0 to 0) | 0.05 (0.05 to 0.05) | 0 (0 to 0) | 0.04 (0.04 to 0.04) | -1.55 (-2.52 to -0.57) |
| Solomon Islands | 0 (0 to 0) | 0.22 (0.04 to 0.49) | 13 (6 to 23) | 7.94 (3.5 to 14.3) | 10.86 (10.1 to 11.63) |
| Somalia | 7 (1 to 25) | 0.45 (0.04 to 1.55) | 1091 (536 to 1966) | 26.98 (13.31 to 48.56) | 7.63 (3.87 to 11.53) |
| South Africa | 1052 (871 to 1273) | 11.3 (9.35 to 13.65) | 36875 (34192 to 39699) | 231.31 (214.24 to 249.27) | 5.77 (1.73 to 9.97) |
| South Sudan | 118 (45 to 332) | 10.02 (3.85 to 28.18) | 1285 (522 to 2958) | 63.22 (25.74 to 146.18) | 4.58 (2.06 to 7.15) |
| Spain | 304 (303 to 305) | 3.16 (3.15 to 3.17) | 85 (85 to 85) | 0.67 (0.67 to 0.67) | -8.17 (-9.16 to -7.17) |
| Sri Lanka | 7 (7 to 7) | 0.15 (0.15 to 0.15) | 37 (36 to 37) | 0.62 (0.62 to 0.63) | 4.9 (3.82 to 5.99) |
| Sudan | 97 (34 to 309) | 2.25 (0.8 to 7.18) | 2914 (1338 to 6846) | 28.74 (13.41 to 66.47) | 6.57 (5.1 to 8.06) |
| Suriname | 15 (15 to 15) | 16.56 (16.42 to 16.69) | 29 (29 to 30) | 19.85 (19.63 to 20.07) | 0.24 (-0.94 to 1.43) |
| Sweden | 3 (3 to 3) | 0.16 (0.16 to 0.16) | 4 (4 to 4) | 0.16 (0.16 to 0.16) | -2.04 (-3.12 to -0.96) |
| Switzerland | 16 (16 to 16) | 0.85 (0.85 to 0.85) | 8 (8 to 8) | 0.38 (0.38 to 0.38) | -5.96 (-7.12 to -4.77) |
| Syrian Arab Republic | 2 (2 to 2) | 0.09 (0.09 to 0.09) | 12 (12 to 12) | 0.32 (0.32 to 0.32) | 4.58 (3.68 to 5.49) |
| Taiwan (Province of China) | 1 (1 to 1) | 0.02 (0.02 to 0.02) | 10 (10 to 10) | 0.15 (0.15 to 0.15) | 5.21 (4.16 to 6.26) |
| Tajikistan | 24 (24 to 24) | 2.43 (2.43 to 2.44) | 60 (60 to 61) | 2.46 (2.45 to 2.48) | -0.7 (-1.39 to -0.01) |
| Thailand | 2366 (2314 to 2421) | 14.58 (14.27 to 14.92) | 2136 (2109 to 2163) | 11.77 (11.63 to 11.91) | -2.8 (-4.29 to -1.28) |
| Timor-Leste | 0 (0 to 1) | 0.24 (0.06 to 0.63) | 50 (29 to 78) | 17.44 (10.33 to 26.59) | 8.73 (5.83 to 11.71) |
| Togo | 190 (90 to 369) | 24.07 (11.36 to 46.7) | 924 (447 to 1657) | 45.66 (22.03 to 82.05) | 0.11 (-2.5 to 2.78) |
| Tokelau | 0 (0 to 0) | 0.1 (0.02 to 0.22) | 0 (0 to 0) | 2.25 (0.69 to 4.7) | 8.63 (7.5 to 9.77) |
| Tonga | 0 (0 to 0) | 0.59 (0.59 to 0.59) | 0 (0 to 0) | 1.03 (1.03 to 1.03) | 1.38 (0.35 to 2.42) |
| Trinidad and Tobago | 36 (36 to 36) | 12.24 (12.17 to 12.3) | 53 (52 to 53) | 14.19 (14.08 to 14.31) | -0.71 (-2.03 to 0.63) |
| Tunisia | 0 (0 to 1) | 0.01 (0 to 0.03) | 9 (3 to 20) | 0.27 (0.08 to 0.61) | 12.49 (10.65 to 14.36) |
| Turkey | 4 (4 to 4) | 0.03 (0.03 to 0.03) | 54 (54 to 54) | 0.23 (0.23 to 0.23) | 8.09 (7.65 to 8.53) |
| Turkmenistan | 13 (13 to 13) | 1.7 (1.7 to 1.7) | 35 (35 to 36) | 2.85 (2.83 to 2.87) | 1.46 (1.03 to 1.89) |
| Tuvalu | 0 (0 to 0) | 0.1 (0.02 to 0.22) | 0 (0 to 0) | 2.36 (0.73 to 5.04) | 8.79 (7.68 to 9.9) |
| Uganda | 24480 (12944 to 39348) | 736.28 (386.15 to 1179.31) | 10930 (5440 to 18790) | 121.28 (59.98 to 208.41) | -6.97 (-7.81 to -6.11) |
| Ukraine | 204 (204 to 205) | 1.52 (1.51 to 1.52) | 1799 (1785 to 1814) | 14.41 (14.3 to 14.52) | 7.54 (6 to 9.1) |
| United Arab Emirates | 0 (0 to 1) | 0.09 (0.04 to 0.18) | 5 (2 to 10) | 0.22 (0.09 to 0.47) | 5.07 (3.03 to 7.16) |
| United Kingdom | 24 (24 to 24) | 0.16 (0.16 to 0.16) | 50 (50 to 50) | 0.3 (0.3 to 0.3) | 0.47 (-0.47 to 1.42) |
| United Republic of Tanzania | 10485 (5341 to 18711) | 192.87 (96.42 to 346.94) | 13585 (6864 to 23378) | 104.03 (52.33 to 179.21) | -4.42 (-6.1 to -2.71) |
| United States of America | 2706 (2701 to 2709) | 3.76 (3.76 to 3.77) | 798 (797 to 798) | 0.97 (0.97 to 0.97) | -6.69 (-7.45 to -5.93) |
| United States Virgin Islands | 2 (2 to 2) | 6.22 (6.2 to 6.25) | 1 (1 to 1) | 4.71 (4.69 to 4.72) | -2.35 (-3.32 to -1.37) |
| Uruguay | 10 (10 to 10) | 1.29 (1.29 to 1.29) | 35 (35 to 35) | 3.99 (3.98 to 4) | 2.97 (2.04 to 3.91) |
| Uzbekistan | 39 (39 to 39) | 0.91 (0.91 to 0.92) | 183 (182 to 183) | 1.96 (1.96 to 1.97) | 2.38 (1.87 to 2.89) |
| Vanuatu | 0 (0 to 0) | 0.21 (0.04 to 0.48) | 6 (3 to 11) | 7.88 (3.45 to 14.31) | 11.06 (10.3 to 11.82) |
| Venezuela (Bolivarian Republic of) | 84 (84 to 85) | 1.9 (1.89 to 1.9) | 349 (347 to 351) | 4.76 (4.74 to 4.78) | 3.66 (3.08 to 4.24) |
| Viet Nam | 33 (11 to 66) | 0.19 (0.06 to 0.38) | 667 (380 to 1206) | 2.45 (1.42 to 4.39) | 7.67 (5.27 to 10.13) |
| Yemen | 3 (1 to 7) | 0.11 (0.02 to 0.3) | 96 (31 to 194) | 1.24 (0.4 to 2.49) | 8.62 (7.75 to 9.49) |
| Zambia | 4947 (2502 to 8721) | 293.36 (141.67 to 526.81) | 9155 (4439 to 16658) | 212.11 (102 to 387.97) | -4.15 (-5.83 to -2.43) |
| Zimbabwe | 7460 (3684 to 13122) | 335.65 (158.84 to 600.43) | 8487 (4320 to 14704) | 233.95 (119.08 to 405.57) | -4.4 (-6.5 to -2.25) |

**Supplementary Table S4**

The DALYs of HIV/AIDS cases and rates among women of childbearing age across 204 countries in 1990 and 2021, and the trends from 1990 to 2021.

| location | Num_1990 | ASR_1990 | Num_2021 | ASR_2021 | EAPC_CI |
| --- | --- | --- | --- | --- | --- |
| Afghanistan | 170 (30 to 372) | 9.74 (1.68 to 21.19) | 1694 (466 to 3705) | 27.12 (7.41 to 58.66) | 4.27 (3.46 to 5.08) |
| Albania | 13 (12 to 14) | 1.48 (1.4 to 1.58) | 12 (11 to 13) | 1.91 (1.79 to 2.19) | -0.85 (-1.96 to 0.27) |
| Algeria | 368 (190 to 620) | 7.29 (3.91 to 11.89) | 4314 (2239 to 8049) | 36.88 (19.21 to 68.65) | 6.06 (4.73 to 7.41) |
| American Samoa | 6 (6 to 6) | 50.66 (50.41 to 50.9) | 22 (21 to 23) | 194.79 (189.71 to 201) | 4.83 (3.18 to 6.51) |
| Andorra | 1 (1 to 2) | 6.63 (3.25 to 12.78) | 1 (0 to 2) | 3.95 (1.97 to 7.45) | -3.8 (-4.61 to -2.98) |
| Angola | 6078 (2800 to 12159) | 274.71 (126.22 to 549.74) | 545470 (325447 to 882890) | 7960.9 (4815.92 to 12698.8) | 8.77 (6.75 to 10.83) |
| Antigua and Barbuda | 73 (73 to 75) | 457.83 (452.58 to 465.31) | 84 (80 to 91) | 326.52 (310.69 to 354.96) | -1.34 (-1.99 to -0.68) |
| Argentina | 6247 (5863 to 6808) | 78.96 (74.1 to 86.04) | 22795 (21791 to 24195) | 185.02 (176.89 to 196.35) | 1.22 (0.06 to 2.39) |
| Armenia | 54 (54 to 55) | 6.22 (6.16 to 6.27) | 172 (153 to 200) | 20.15 (17.96 to 23.42) | 3.8 (3.45 to 4.15) |
| Australia | 407 (376 to 451) | 8.91 (8.22 to 9.87) | 547 (409 to 798) | 8.45 (6.33 to 12.28) | -1.85 (-2.63 to -1.07) |
| Austria | 573 (559 to 592) | 28.11 (27.44 to 29.06) | 447 (338 to 640) | 20.83 (15.84 to 29.54) | -2.94 (-3.73 to -2.14) |
| Azerbaijan | 295 (289 to 301) | 16.57 (16.31 to 16.9) | 479 (442 to 540) | 15.88 (14.64 to 17.89) | -1.14 (-1.76 to -0.52) |
| Bahamas | 984 (954 to 1023) | 1417.78 (1375.39 to 1473.42) | 1928 (1843 to 2055) | 1754.16 (1676.48 to 1870.03) | -2 (-3.22 to -0.76) |
| Bahrain | 18 (18 to 19) | 16.32 (16.07 to 16.79) | 230 (228 to 235) | 68.36 (67.62 to 69.65) | 6.86 (5.49 to 8.24) |
| Bangladesh | 12 (5 to 22) | 0.05 (0.02 to 0.1) | 9238 (4741 to 16104) | 20.36 (10.5 to 35.33) | 22.69 (19.94 to 25.51) |
| Barbados | 288 (282 to 297) | 419.04 (410.99 to 432.26) | 317 (303 to 336) | 424.91 (406.68 to 450.4) | -0.79 (-1.69 to 0.12) |
| Belarus | 1162 (1127 to 1181) | 43.64 (42.32 to 44.37) | 6367 (6085 to 6850) | 258.71 (247.32 to 278.4) | 6.74 (6.23 to 7.26) |
| Belgium | 1217 (1192 to 1256) | 47.59 (46.57 to 49.12) | 894 (715 to 1143) | 33.64 (26.97 to 42.91) | -2.84 (-3.52 to -2.15) |
| Belize | 286 (282 to 291) | 763.58 (752.91 to 776.75) | 1356 (1299 to 1459) | 1171.73 (1122.79 to 1260.79) | 1.12 (-0.09 to 2.34) |
| Benin | 796 (262 to 1937) | 71.14 (23.13 to 173.72) | 39683 (21218 to 65712) | 1383.02 (744.96 to 2277.22) | 2.45 (-1.2 to 6.23) |
| Bermuda | 93 (92 to 94) | 499.84 (493.93 to 507.53) | 42 (41 to 43) | 284.97 (279.49 to 293.46) | -2.13 (-2.82 to -1.44) |
| Bhutan | 17 (4 to 44) | 13.46 (3.28 to 34.11) | 145 (57 to 317) | 69.89 (27.54 to 153.71) | 4.83 (2.45 to 7.26) |
| Bolivia (Plurinational State of) | 88 (9 to 364) | 5.9 (0.61 to 24.68) | 4985 (2032 to 10165) | 162.04 (65.96 to 331) | 9.94 (5.25 to 14.85) |
| Bosnia and Herzegovina | 74 (73 to 74) | 6.27 (6.24 to 6.31) | 69 (68 to 69) | 9.41 (9.37 to 9.47) | 1.69 (1.15 to 2.24) |
| Botswana | 30085 (16053 to 54055) | 9379.38 (4857.5 to 17096.44) | 122066 (65101 to 213014) | 17890.06 (9530.27 to 31250.18) | -1.83 (-4.12 to 0.52) |
| Brazil | 71941 (70028 to 74627) | 187.58 (182.7 to 194.55) | 186816 (177002 to 201092) | 298.91 (283.13 to 321.88) | -0.33 (-1.19 to 0.54) |
| Brunei Darussalam | 5 (4 to 5) | 6.88 (6.27 to 8.01) | 32 (27 to 39) | 24.16 (20.63 to 29.65) | 3.41 (2.92 to 3.9) |
| Bulgaria | 401 (395 to 406) | 19.79 (19.55 to 20.06) | 443 (418 to 480) | 30.77 (28.96 to 33.44) | 0.74 (-0.26 to 1.75) |
| Burkina Faso | 226959 (123431 to 381807) | 11478.97 (6259.08 to 19161.08) | 46253 (25093 to 75837) | 961.6 (522.32 to 1573.83) | -9.46 (-10.48 to -8.43) |
| Burundi | 110231 (50044 to 243215) | 9033.61 (4076.33 to 20224.55) | 40125 (20742 to 70134) | 1479.47 (764.62 to 2593.4) | -8.13 (-10.01 to -6.21) |
| Cabo Verde | 544 (180 to 1274) | 724.2 (238.72 to 1693.1) | 870 (257 to 2613) | 589.36 (174.96 to 1764.45) | -3 (-4.76 to -1.21) |
| Cambodia | 4 (2 to 7) | 0.16 (0.09 to 0.28) | 18631 (7746 to 35567) | 423.6 (175.73 to 809.3) | 12.69 (4.73 to 21.25) |
| Cameroon | 36889 (19297 to 65658) | 1580.99 (811.94 to 2835.62) | 448857 (279299 to 683353) | 6520.91 (4074.18 to 9873.88) | 2.67 (0.22 to 5.17) |
| Canada | 2612 (2427 to 2914) | 33.31 (30.91 to 37.25) | 2557 (1767 to 3828) | 28.06 (19.36 to 42.01) | -2.2 (-2.82 to -1.58) |
| Central African Republic | 55806 (24133 to 107777) | 8605.99 (3668.31 to 16775.08) | 115731 (66675 to 193186) | 9457.17 (5558.15 to 15503.53) | -2.45 (-4.12 to -0.75) |
| Chad | 18190 (8928 to 35427) | 1419.57 (695.03 to 2774.51) | 70396 (36518 to 125528) | 2122.34 (1099.88 to 3781.71) | -0.98 (-2.8 to 0.88) |
| Chile | 1034 (1002 to 1078) | 29.09 (28.21 to 30.34) | 4247 (3809 to 5117) | 84.93 (76.14 to 102.26) | 2.73 (1.99 to 3.47) |
| China | 17146 (2176 to 30268) | 5.68 (0.69 to 10.03) | 197134 (141450 to 263730) | 55.27 (40.04 to 72.74) | 6.24 (5.11 to 7.38) |
| Colombia | 5622 (5551 to 5784) | 68.62 (67.81 to 70.44) | 27257 (26077 to 29300) | 204.33 (195.48 to 219.66) | 3.91 (2.82 to 5.01) |
| Comoros | 2 (0 to 9) | 2.56 (0.24 to 9.35) | 23 (10 to 44) | 12.33 (5.27 to 23.55) | 5.17 (3.24 to 7.14) |
| Congo | 45904 (21782 to 83343) | 8503.52 (3984.88 to 15539.01) | 112457 (66922 to 182803) | 8224.15 (4932.49 to 13287.29) | -2.32 (-3.39 to -1.23) |
| Cook Islands | 0 (0 to 1) | 5.43 (1.21 to 11.9) | 7 (3 to 13) | 160.5 (61.47 to 298.11) | 9.84 (8.8 to 10.89) |
| Costa Rica | 647 (639 to 665) | 89.3 (88.09 to 91.85) | 1935 (1882 to 2021) | 142.83 (138.81 to 149.37) | 1.77 (1.47 to 2.08) |
| Coted'Ivoire | 350812 (176007 to 655011) | 13441.4 (6651.7 to 25093.85) | 254506 (143645 to 413611) | 4230.73 (2389.64 to 6885.31) | -5.47 (-7.04 to -3.88) |
| Croatia | 71 (70 to 73) | 5.94 (5.85 to 6.05) | 57 (53 to 64) | 6.33 (5.9 to 7.04) | -0.58 (-1.41 to 0.26) |
| Cuba | 1407 (1391 to 1435) | 46.97 (46.46 to 47.96) | 1605 (1422 to 1977) | 60.69 (53.88 to 74.7) | 0.71 (0.11 to 1.3) |
| Cyprus | 20 (20 to 20) | 9.84 (9.76 to 9.96) | 70 (68 to 74) | 17.6 (16.82 to 18.91) | 1.24 (0.06 to 2.44) |
| Czechia | 87 (85 to 90) | 3.41 (3.33 to 3.53) | 103 (87 to 130) | 4.62 (3.91 to 5.74) | 0.4 (-0.38 to 1.18) |
| Democratic People's Republic of Korea | 53 (2 to 152) | 0.95 (0.04 to 2.75) | 5286 (1076 to 18841) | 77.51 (15.72 to 275.64) | 13.11 (10.17 to 16.13) |
| Democratic Republic of the Congo | 435872 (251839 to 724953) | 5489.61 (3199.4 to 9045.07) | 189079 (104010 to 332731) | 977.77 (542.33 to 1701.89) | -5.53 (-7.22 to -3.82) |
| Denmark | 332 (323 to 343) | 24.68 (24.02 to 25.53) | 171 (156 to 191) | 12.86 (11.82 to 14.37) | -4.19 (-4.96 to -3.41) |
| Djibouti | 17 (2 to 57) | 17.46 (1.68 to 59.12) | 20960 (11942 to 34798) | 6481.63 (3700.51 to 10756.05) | 12.94 (7.72 to 18.41) |
| Dominica | 47 (46 to 48) | 307.89 (304.02 to 313.14) | 53 (51 to 56) | 320.35 (311.59 to 338.21) | -0.37 (-1.31 to 0.58) |
| Dominican Republic | 6635 (2374 to 15478) | 347.67 (120.41 to 823.98) | 12395 (3973 to 26901) | 434.3 (139.3 to 942.07) | -2.45 (-4.61 to -0.23) |
| Ecuador | 1241 (1203 to 1301) | 53.26 (51.78 to 55.56) | 11620 (11078 to 12463) | 246.93 (235.48 to 264.7) | 5.85 (4.53 to 7.19) |
| Egypt | 1380 (1367 to 1394) | 11.21 (11.11 to 11.33) | 7949 (7748 to 8315) | 31.46 (30.69 to 32.87) | 2.93 (2.27 to 3.58) |
| El Salvador | 1581 (1563 to 1613) | 129 (127.5 to 131.72) | 4424 (4228 to 4747) | 250.95 (239.8 to 269.32) | 2.27 (0.8 to 3.77) |
| Equatorial Guinea | 663 (308 to 1434) | 699.94 (324.44 to 1518.14) | 53789 (25820 to 103213) | 15872.64 (7660.5 to 30100.21) | 8.47 (6.13 to 10.86) |
| Eritrea | 13486 (5857 to 28568) | 1789.22 (772.93 to 3796.6) | 22314 (13551 to 35075) | 1484.45 (914.12 to 2314.27) | -3.2 (-5.1 to -1.26) |
| Estonia | 104 (103 to 104) | 26.18 (26.02 to 26.33) | 570 (532 to 630) | 183.87 (171.05 to 204.11) | 7.2 (6.83 to 7.56) |
| Eswatini | 670 (215 to 1801) | 320.3 (102.41 to 866.93) | 92459 (44857 to 174690) | 30103.88 (14722.61 to 56443.76) | 8.5 (3.83 to 13.37) |
| Ethiopia | 227558 (121406 to 416476) | 2077.66 (1101.85 to 3788.55) | 411410 (237184 to 653599) | 1737.15 (1007.84 to 2744.8) | -3.78 (-5.72 to -1.81) |
| Fiji | 264 (262 to 267) | 140.88 (139.84 to 142) | 570 (559 to 587) | 249.34 (244.31 to 256.78) | 1.27 (-0.09 to 2.65) |
| Finland | 153 (149 to 157) | 11.3 (11.04 to 11.63) | 102 (84 to 128) | 8.42 (6.99 to 10.54) | -2.58 (-3.23 to -1.92) |
| France | 19850 (19163 to 20809) | 133.72 (129.08 to 140.17) | 5768 (4872 to 6892) | 37.91 (32.05 to 45.27) | -6.43 (-7.35 to -5.5) |
| Gabon | 4578 (2397 to 8347) | 2165.2 (1135.85 to 3934.7) | 30034 (16156 to 50295) | 6732.64 (3638.11 to 11208.11) | 1.15 (-0.82 to 3.16) |
| Gambia | 581 (273 to 1140) | 265.04 (123.27 to 517.68) | 26293 (13301 to 46399) | 4913.67 (2527.23 to 8523.48) | 7.3 (4.79 to 9.86) |
| Georgia | 149 (148 to 152) | 10.62 (10.52 to 10.82) | 359 (332 to 413) | 40.29 (37.29 to 46.2) | 4.69 (4.47 to 4.91) |
| Germany | 9502 (9203 to 9917) | 46 (44.53 to 48.04) | 3926 (3134 to 5205) | 20.68 (16.58 to 27.26) | -4.07 (-4.83 to -3.3) |
| Ghana | 69881 (37872 to 119323) | 2064.39 (1117.17 to 3514.21) | 411335 (254109 to 634367) | 4809.7 (3016.71 to 7327.99) | 0.71 (-0.97 to 2.43) |
| Greece | 272 (264 to 281) | 10.73 (10.42 to 11.11) | 234 (186 to 306) | 10.1 (8.07 to 13.14) | -1.71 (-2.41 to -1.01) |
| Greenland | 20 (19 to 22) | 145.6 (138.36 to 155.86) | 16 (14 to 19) | 118.78 (104.27 to 142.14) | -2.32 (-3.33 to -1.31) |
| Grenada | 63 (62 to 64) | 346.7 (343.35 to 351.23) | 60 (59 to 62) | 240.59 (236.62 to 246.35) | -1.24 (-1.9 to -0.57) |
| Guam | 18 (18 to 18) | 52.37 (52.19 to 52.62) | 120 (119 to 122) | 330.33 (327.2 to 334.86) | 6.03 (4.72 to 7.35) |
| Guatemala | 4534 (4438 to 4679) | 270.29 (264.78 to 278.85) | 8477 (8181 to 8968) | 203.16 (196.1 to 214.93) | -1.07 (-1.86 to -0.27) |
| Guinea | 8946 (4532 to 17355) | 660.75 (333.99 to 1280.16) | 76406 (44141 to 122500) | 2589.11 (1511.88 to 4107.38) | 2.27 (0.16 to 4.42) |
| Guinea-Bissau | 1095 (467 to 2109) | 490.68 (210.16 to 941.48) | 27888 (11156 to 56241) | 5718.56 (2264.35 to 11570.33) | 5.88 (3.73 to 8.07) |
| Guyana | 2028 (1998 to 2067) | 1058.8 (1043.54 to 1079.32) | 3374 (3258 to 3541) | 1705.94 (1647.15 to 1790.02) | 0.67 (-0.88 to 2.24) |
| Haiti | 131274 (63698 to 236142) | 8737.78 (4233.23 to 15703.26) | 105247 (55822 to 172034) | 3068.16 (1642.12 to 4988.47) | -4.93 (-5.97 to -3.88) |
| Honduras | 4283 (4224 to 4369) | 424.87 (419.04 to 433.41) | 3839 (3775 to 3934) | 138.49 (136.14 to 141.99) | -3.19 (-3.28 to -3.09) |
| Hungary | 722 (703 to 754) | 27.93 (27.24 to 29.09) | 424 (418 to 432) | 19.7 (19.47 to 20.02) | -2.16 (-2.95 to -1.36) |
| Iceland | 15 (15 to 16) | 23.16 (22.15 to 24.65) | 17 (14 to 23) | 20.85 (16.34 to 27.97) | -1.77 (-2.4 to -1.12) |
| India | 4377 (2025 to 8576) | 2.19 (1.01 to 4.33) | 822410 (550334 to 1157415) | 221.17 (148.3 to 311.1) | 9.91 (4.83 to 15.23) |
| Indonesia | 5728 (5693 to 5775) | 13.17 (13.1 to 13.26) | 50634 (47874 to 54854) | 65.34 (61.74 to 70.84) | 6.17 (5.17 to 7.18) |
| Iran (Islamic Republic of) | 264 (256 to 279) | 2.31 (2.24 to 2.44) | 5973 (5691 to 6326) | 23.64 (22.5 to 25.08) | 9.51 (8.53 to 10.5) |
| Iraq | 141 (137 to 149) | 3.94 (3.83 to 4.15) | 2610 (2485 to 2944) | 25.82 (24.6 to 29.15) | 7.15 (6.52 to 7.77) |
| Ireland | 146 (142 to 152) | 16.88 (16.38 to 17.54) | 188 (139 to 259) | 15.18 (11.29 to 20.85) | -2.2 (-2.86 to -1.53) |
| Israel | 393 (385 to 408) | 32.69 (31.97 to 33.9) | 557 (447 to 709) | 24.92 (20.05 to 31.61) | -2.59 (-3.26 to -1.93) |
| Italy | 17755 (17091 to 18668) | 124.16 (119.51 to 130.53) | 5139 (4407 to 6145) | 36.79 (31.74 to 43.81) | -6.57 (-7.57 to -5.55) |
| Jamaica | 3316 (3263 to 3396) | 606.36 (596.7 to 620.67) | 7645 (7393 to 8021) | 978.55 (946.35 to 1026.34) | 0.76 (-0.29 to 1.83) |
| Japan | 257 (223 to 321) | 0.78 (0.67 to 0.97) | 756 (583 to 998) | 2.87 (2.2 to 3.8) | 3 (2.19 to 3.82) |
| Jordan | 29 (28 to 29) | 4.1 (4.05 to 4.2) | 627 (624 to 633) | 21.32 (21.21 to 21.49) | 5.87 (5.16 to 6.59) |
| Kazakhstan | 1124 (1110 to 1141) | 27.2 (26.88 to 27.6) | 3945 (3654 to 4434) | 76.25 (70.63 to 85.72) | 2.41 (1.61 to 3.21) |
| Kenya | 478266 (271265 to 797859) | 9817.34 (5455.24 to 16428.66) | 675169 (419736 to 1023913) | 5664.9 (3527.73 to 8673.06) | -4.31 (-6.32 to -2.26) |
| Kiribati | 28 (27 to 28) | 154.03 (152.98 to 155.26) | 60 (60 to 61) | 191.58 (189.82 to 194) | -0.14 (-1.26 to 1) |
| Kuwait | 28 (28 to 29) | 7.04 (7 to 7.1) | 97 (95 to 99) | 5.8 (5.72 to 5.95) | -0.09 (-1.39 to 1.23) |
| Kyrgyzstan | 559 (555 to 564) | 57.09 (56.67 to 57.59) | 2879 (2738 to 3086) | 164.68 (156.68 to 176.52) | 3.14 (2.82 to 3.46) |
| Lao People's Democratic Republic | 3 (1 to 7) | 0.36 (0.15 to 0.73) | 2201 (956 to 4464) | 113.95 (49.11 to 232.62) | 20.34 (14.92 to 26.02) |
| Latvia | 629 (619 to 638) | 92.95 (91.57 to 94.31) | 2859 (2786 to 2957) | 651.23 (634.84 to 673.42) | 6.68 (6.36 to 7.01) |
| Lebanon | 85 (45 to 141) | 11.95 (6.47 to 19.51) | 1176 (701 to 1833) | 72.39 (43.14 to 113.16) | 5.98 (5.54 to 6.43) |
| Lesotho | 14129 (7510 to 24317) | 3815.39 (2003.98 to 6610.76) | 157603 (90701 to 251381) | 33601.14 (19331.21 to 53634.1) | 4.05 (1.43 to 6.74) |
| Liberia | 3217 (1394 to 7602) | 580.7 (249.96 to 1374.47) | 30238 (17265 to 49240) | 2357.78 (1348.58 to 3830.32) | 3.01 (0.57 to 5.5) |
| Libya | 99 (6 to 474) | 13.32 (0.78 to 62.12) | 1470 (12 to 13300) | 70.73 (0.59 to 636.53) | 5.71 (4.67 to 6.75) |
| Lithuania | 923 (904 to 957) | 97.72 (95.74 to 101.3) | 3518 (3483 to 3578) | 571.96 (566.52 to 580.82) | 6.07 (5.78 to 6.37) |
| Luxembourg | 36 (34 to 38) | 33.93 (32.08 to 36.54) | 42 (33 to 55) | 24.32 (19.3 to 31.74) | -2.96 (-3.65 to -2.27) |
| Madagascar | 28 (7 to 70) | 1.04 (0.26 to 2.64) | 74238 (39710 to 129524) | 1158.78 (626.31 to 1996.01) | 20.85 (15.83 to 26.08) |
| Malawi | 352465 (197663 to 588061) | 16416.09 (9043.62 to 27478.98) | 457792 (259667 to 748590) | 10642.49 (6050.21 to 17422.34) | -4.67 (-6.51 to -2.8) |
| Malaysia | 2500 (2442 to 2572) | 60.51 (59.13 to 62.21) | 14493 (13855 to 15455) | 171.61 (164.11 to 182.92) | 4.28 (3.29 to 5.28) |
| Maldives | 4 (4 to 4) | 9.06 (8.68 to 9.55) | 64 (63 to 66) | 53.12 (52.5 to 54.49) | 6.66 (5.65 to 7.67) |
| Mali | 12458 (5974 to 25007) | 663.27 (315.8 to 1334.45) | 101698 (57067 to 173715) | 2208.16 (1264.3 to 3700.01) | 1.34 (-0.64 to 3.36) |
| Malta | 21 (20 to 22) | 20.82 (20.17 to 21.79) | 22 (16 to 30) | 20.85 (15.57 to 28.56) | -1.17 (-1.71 to -0.62) |
| Marshall Islands | 1 (0 to 2) | 12.56 (2.92 to 27.33) | 65 (31 to 114) | 449.39 (212.24 to 791.05) | 10.9 (10.16 to 11.65) |
| Mauritania | 17 (7 to 29) | 4.13 (1.73 to 6.96) | 56 (23 to 98) | 5.95 (2.4 to 10.48) | 0.03 (-1.45 to 1.54) |
| Mauritius | 46 (45 to 47) | 15.66 (15.34 to 16.02) | 656 (631 to 695) | 198.52 (190.81 to 210.5) | 16.4 (13.75 to 19.12) |
| Mexico | 19576 (19351 to 19871) | 100.37 (99.28 to 101.81) | 48634 (47099 to 50826) | 136.9 (132.56 to 143.11) | 0.58 (0.16 to 1) |
| Micronesia (Federated States of) | 3 (1 to 6) | 13.26 (3.12 to 28.44) | 116 (54 to 207) | 472.71 (220.11 to 833.81) | 10.94 (10.23 to 11.65) |
| Monaco | 1 (0 to 2) | 14.55 (5.71 to 29.79) | 1 (0 to 1) | 8.85 (4.55 to 16.66) | -2.79 (-3.43 to -2.14) |
| Mongolia | 27 (27 to 27) | 6.33 (6.32 to 6.33) | 129 (123 to 144) | 14.19 (13.53 to 15.77) | 1.67 (0.94 to 2.4) |
| Montenegro | 13 (13 to 13) | 8.16 (8.05 to 8.36) | 13 (13 to 15) | 9.37 (8.84 to 10.19) | -0.77 (-1.76 to 0.23) |
| Morocco | 1610 (654 to 3404) | 27.33 (11.2 to 57.32) | 5081 (2271 to 10065) | 51.75 (23.17 to 102.29) | 2.44 (0.38 to 4.54) |
| Mozambique | 41379 (26677 to 65149) | 1336.73 (864.98 to 2108.03) | 1418187 (1228796 to 1657683) | 19953.02 (17463.22 to 23140.75) | 6.89 (4.4 to 9.45) |
| Myanmar | 1131 (561 to 2102) | 10.62 (5.23 to 19.85) | 83998 (39457 to 144183) | 560.47 (263.32 to 960.82) | 8.34 (4.24 to 12.61) |
| Namibia | 7472 (3939 to 12990) | 2315.06 (1200.92 to 4063.81) | 76111 (43638 to 126763) | 12098.01 (6956.54 to 20112.94) | 1.67 (-1.16 to 4.58) |
| Nauru | 0 (0 to 0) | 5.45 (1.32 to 11.73) | 5 (2 to 9) | 165.85 (62.84 to 317.48) | 9.78 (8.74 to 10.82) |
| Nepal | 0 to 0 | 0 (0 to 0) | 5468 (2366 to 11215) | 63.49 (27.21 to 131.11) | 22.75 (13.78 to 32.43) |
| Netherlands | 1031 (988 to 1096) | 25.21 (24.14 to 26.82) | 703 (540 to 960) | 18.23 (14.09 to 24.72) | -3.04 (-3.78 to -2.29) |
| New Zealand | 95 (91 to 101) | 10.48 (10.07 to 11.14) | 86 (66 to 120) | 6.91 (5.32 to 9.63) | -1.3 (-2.35 to -0.24) |
| Nicaragua | 529 (525 to 535) | 65.54 (65.09 to 66.27) | 2358 (2237 to 2550) | 130.03 (123.42 to 140.51) | 2.94 (2.53 to 3.35) |
| Niger | 6557 (3069 to 13487) | 395.56 (184.29 to 815.05) | 18438 (8889 to 34671) | 427.31 (206.4 to 806.07) | -1.7 (-4.06 to 0.73) |
| Nigeria | 200812 (116646 to 340911) | 1063.19 (613.79 to 1800.53) | 1766837 (1187995 to 2541994) | 3613.08 (2457.76 to 5131.57) | 0.82 (-1.02 to 2.71) |
| Niue | 0 (0 to 0) | 5.78 (1.37 to 12.49) | 1 (0 to 1) | 137.16 (48.59 to 270.53) | 8.89 (7.85 to 9.94) |
| North Macedonia | 9 (9 to 9) | 1.79 (1.77 to 1.82) | 12 (11 to 12) | 2.16 (2.11 to 2.23) | -0.58 (-1.61 to 0.46) |
| Northern Mariana Islands | 9 (9 to 9) | 67.94 (67.48 to 68.47) | 25 (24 to 25) | 210.49 (207.13 to 215.58) | 3.53 (2.11 to 4.98) |
| Norway | 71 (68 to 76) | 6.68 (6.38 to 7.07) | 266 (204 to 356) | 21.35 (16.39 to 28.67) | 0.2 (-1.09 to 1.51) |
| Oman | 42 (41 to 42) | 13.93 (13.73 to 14.2) | 834 (806 to 875) | 76.09 (73.47 to 79.83) | 6.27 (5.63 to 6.92) |
| Pakistan | 2 (0 to 13) | 0.01 (0 to 0.06) | 132786 (1080 to 778900) | 227.93 (1.77 to 1351.35) | 40.56 (37.56 to 43.61) |
| Palau | 0 (0 to 0) | 5.5 (1.31 to 11.93) | 5 (2 to 10) | 134.71 (49.12 to 258.4) | 9.22 (8.23 to 10.23) |
| Palestine | 5 (5 to 5) | 1.28 (1.26 to 1.31) | 164 (155 to 179) | 13.67 (12.89 to 14.94) | 9.46 (8.19 to 10.74) |
| Panama | 1496 (1466 to 1542) | 261.78 (256.94 to 269.53) | 5347 (5142 to 5658) | 500.85 (481.59 to 530.14) | 1.63 (0.76 to 2.5) |
| Papua New Guinea | 27 (8 to 64) | 2.9 (0.88 to 6.8) | 17420 (9003 to 30776) | 696.5 (360.13 to 1229.45) | 13.48 (8.12 to 19.12) |
| Paraguay | 592 (577 to 614) | 66.81 (65.21 to 69.13) | 3735 (3584 to 3974) | 200.04 (192.06 to 212.7) | 2.84 (1.97 to 3.72) |
| Peru | 3501 (3379 to 3664) | 69.98 (67.79 to 72.91) | 15907 (15268 to 16902) | 161.97 (155.47 to 172.1) | 1.9 (0.43 to 3.39) |
| Philippines | 164 (161 to 169) | 1.13 (1.11 to 1.17) | 59810 (56674 to 66408) | 207.73 (196.93 to 230.6) | 20.91 (17.43 to 24.49) |
| Poland | 123 (116 to 134) | 1.25 (1.18 to 1.37) | 1305 (1175 to 1495) | 13.12 (11.87 to 14.94) | 8.47 (5.74 to 11.28) |
| Portugal | 3036 (3007 to 3070) | 121.11 (119.94 to 122.47) | 2302 (2162 to 2477) | 87.67 (82.73 to 93.92) | -3.95 (-5.1 to -2.78) |
| Puerto Rico | 7443 (7332 to 7599) | 783.77 (772.1 to 800.24) | 1687 (1659 to 1728) | 211.58 (208.05 to 216.74) | -6.12 (-6.86 to -5.38) |
| Qatar | 20 (20 to 21) | 25.23 (24.54 to 26.32) | 134 (132 to 135) | 21.42 (21.22 to 21.7) | -1.02 (-2 to -0.03) |
| Republic of Korea | 160 (114 to 191) | 1.27 (0.9 to 1.52) | 272 (233 to 326) | 2.13 (1.82 to 2.57) | 1.27 (0.92 to 1.63) |
| Republic of Moldova | 460 (455 to 471) | 39.11 (38.65 to 40.02) | 2744 (2629 to 2922) | 269.15 (258.08 to 286.22) | 6.79 (6.43 to 7.15) |
| Romania | 1796 (1767 to 1828) | 32.02 (31.49 to 32.6) | 2412 (2200 to 2766) | 63.76 (58.54 to 72.32) | 1.94 (0.94 to 2.94) |
| Russian Federation | 27608 (27219 to 28424) | 70.85 (69.84 to 72.91) | 307053 (294163 to 325967) | 789.14 (755.6 to 838.55) | 9.65 (9.17 to 10.13) |
| Rwanda | 55893 (23647 to 137941) | 3596.49 (1503.79 to 8935.01) | 106502 (59402 to 173357) | 3318.63 (1850.12 to 5413.16) | -3.85 (-5.73 to -1.94) |
| Saint Kitts and Nevis | 11 (4 to 26) | 109.6 (37.35 to 260.59) | 151 (86 to 236) | 904.58 (512.12 to 1423.78) | 5.24 (3.32 to 7.19) |
| Saint Lucia | 97 (95 to 98) | 320.21 (315.95 to 325.52) | 101 (98 to 106) | 208.79 (202.95 to 220.02) | -1.55 (-2.23 to -0.88) |
| Saint Vincent and the Grenadines | 233 (229 to 237) | 989.08 (973.84 to 1007.06) | 255 (249 to 267) | 902.88 (880.16 to 943.55) | -0.54 (-1.57 to 0.5) |
| Samoa | 4 (1 to 10) | 13.08 (3.04 to 28.71) | 207 (94 to 369) | 453.11 (206.56 to 801.28) | 10.87 (10.19 to 11.56) |
| San Marino | 1 (0 to 2) | 14.53 (5.71 to 29.67) | 1 (0 to 1) | 7.43 (3.77 to 14.07) | -3.43 (-4.03 to -2.83) |
| Sao Tome and Principe | 1 (0 to 1) | 3.05 (1.49 to 5.55) | 2 (1 to 3) | 3.98 (2.78 to 5.51) | -2.24 (-3.78 to -0.68) |
| Saudi Arabia | 1514 (1484 to 1554) | 52.91 (52 to 54.13) | 17186 (16676 to 18742) | 152.84 (148.25 to 166.68) | 3.9 (2.72 to 5.08) |
| Senegal | 7096 (3745 to 12729) | 439.52 (232.46 to 782.64) | 30988 (16433 to 51974) | 893.54 (475.28 to 1492.01) | 0.26 (-1.81 to 2.37) |
| Serbia | 259 (234 to 281) | 11.01 (9.97 to 11.98) | 197 (191 to 213) | 9.49 (9.21 to 10.16) | -1.8 (-2.72 to -0.88) |
| Seychelles | 8 (7 to 8) | 46.18 (45.47 to 47.15) | 83 (81 to 88) | 325.2 (316.03 to 344.56) | 8.22 (6.85 to 9.62) |
| Sierra Leone | 3374 (1556 to 7272) | 336.58 (153.86 to 727.97) | 43062 (23234 to 71421) | 2104.96 (1138.6 to 3483.94) | 4.16 (1.69 to 6.69) |
| Singapore | 23 (22 to 26) | 2.46 (2.29 to 2.72) | 87 (57 to 132) | 5.14 (3.49 to 7.62) | 1.09 (0.36 to 1.83) |
| Slovakia | 23 (22 to 23) | 1.7 (1.68 to 1.73) | 25 (24 to 27) | 1.98 (1.91 to 2.1) | -0.57 (-1.51 to 0.37) |
| Slovenia | 15 (14 to 15) | 2.93 (2.87 to 2.98) | 12 (11 to 14) | 2.84 (2.65 to 3.19) | -1.14 (-2.08 to -0.18) |
| Solomon Islands | 9 (2 to 19) | 13.52 (3.12 to 29.27) | 754 (347 to 1352) | 461.7 (213.31 to 821.27) | 10.72 (10.01 to 11.42) |
| Somalia | 549 (100 to 1600) | 33.01 (5.98 to 96.28) | 61814 (31509 to 109343) | 1483.11 (759.66 to 2620.1) | 6.98 (3.39 to 10.7) |
| South Africa | 73016 (60427 to 88249) | 757.52 (627.23 to 913.65) | 2512397 (2295756 to 2730265) | 15756.93 (14378.51 to 17143.17) | 5.96 (2.13 to 9.94) |
| South Sudan | 7505 (3063 to 20148) | 610.38 (248.38 to 1635.85) | 76115 (33972 to 166072) | 3686.97 (1646.39 to 8080.22) | 4.42 (1.99 to 6.9) |
| Spain | 19478 (18805 to 20429) | 202.09 (195.07 to 212) | 5134 (4689 to 5728) | 42.28 (38.56 to 47.27) | -8.03 (-8.97 to -7.07) |
| Sri Lanka | 391 (382 to 405) | 8.73 (8.54 to 9.04) | 2046 (1998 to 2133) | 35.54 (34.69 to 37.09) | 4.85 (3.78 to 5.93) |
| Sudan | 5902 (2201 to 18445) | 133 (49.54 to 414.87) | 165366 (76664 to 389389) | 1593.7 (750.71 to 3695.17) | 6.36 (4.91 to 7.83) |
| Suriname | 857 (846 to 873) | 935.97 (923.45 to 952.68) | 1757 (1682 to 1877) | 1200.11 (1148.21 to 1282.07) | 0.45 (-0.71 to 1.63) |
| Sweden | 204 (194 to 219) | 9.53 (9.06 to 10.27) | 313 (248 to 401) | 13.1 (10.4 to 16.79) | -0.93 (-1.89 to 0.04) |
| Switzerland | 984 (924 to 1079) | 53.41 (50.07 to 58.72) | 669 (536 to 856) | 30.6 (24.77 to 38.89) | -4.97 (-6.08 to -3.86) |
| Syrian Arab Republic | 122 (121 to 124) | 5.07 (5.02 to 5.17) | 632 (625 to 644) | 17.55 (17.36 to 17.91) | 4.52 (3.64 to 5.42) |
| Taiwan (Province of China) | 70 (66 to 73) | 1.25 (1.19 to 1.31) | 553 (530 to 596) | 8.89 (8.52 to 9.57) | 5.03 (4 to 6.07) |
| Tajikistan | 1342 (1330 to 1358) | 132.79 (131.67 to 134.23) | 3482 (3364 to 3639) | 140.47 (135.58 to 146.94) | -0.59 (-1.26 to 0.09) |
| Thailand | 140478 (137164 to 144154) | 854.74 (834.77 to 876.84) | 129003 (119961 to 144013) | 733.76 (683.66 to 817.26) | -2.68 (-4.16 to -1.17) |
| Timor-Leste | 33 (12 to 76) | 16.85 (6.19 to 39.45) | 2825 (1653 to 4420) | 964.43 (578.78 to 1473.55) | 8.27 (5.52 to 11.09) |
| Togo | 12236 (6300 to 22648) | 1490.68 (761.68 to 2761.51) | 55913 (29688 to 95777) | 2721.18 (1448.31 to 4662.29) | -0.04 (-2.54 to 2.52) |
| Tokelau | 0 (0 to 0) | 5.99 (1.44 to 12.84) | 0 (0 to 1) | 136.05 (47.62 to 273.48) | 8.72 (7.69 to 9.76) |
| Tonga | 7 (6 to 7) | 33.11 (32.4 to 33.85) | 19 (17 to 22) | 79.7 (68.92 to 93) | 2.24 (1.35 to 3.13) |
| Trinidad and Tobago | 2054 (2017 to 2121) | 683.52 (671.49 to 705.26) | 3131 (3010 to 3296) | 861.1 (827.09 to 907.9) | -0.51 (-1.83 to 0.83) |
| Tunisia | 8 (1 to 31) | 0.43 (0.03 to 1.6) | 535 (184 to 1156) | 16.34 (5.66 to 35.32) | 12.33 (10.56 to 14.13) |
| Turkey | 208 (198 to 218) | 1.57 (1.49 to 1.64) | 2989 (2906 to 3146) | 13.15 (12.76 to 13.87) | 7.97 (7.52 to 8.41) |
| Turkmenistan | 782 (772 to 795) | 95.55 (94.39 to 97.01) | 1981 (1950 to 2025) | 158.56 (156.1 to 162.11) | 1.42 (0.99 to 1.86) |
| Tuvalu | 0 (0 to 0) | 6.04 (1.45 to 12.94) | 4 (1 to 8) | 144.43 (51.25 to 296.73) | 8.87 (7.86 to 9.9) |
| Uganda | 1480663 (814470 to 2347779) | 42482.92 (23097.86 to 67158.2) | 698384 (382893 to 1149278) | 7483.71 (4114.11 to 12279.28) | -6.67 (-7.43 to -5.9) |
| Ukraine | 11324 (11110 to 11768) | 84.42 (82.79 to 87.76) | 99782 (95794 to 106299) | 818.74 (784.43 to 875.6) | 7.59 (6.04 to 9.16) |
| United Arab Emirates | 18 (8 to 37) | 4.97 (2.19 to 10.33) | 269 (114 to 534) | 13.86 (6 to 27.43) | 5.09 (3.1 to 7.12) |
| United Kingdom | 1526 (1418 to 1688) | 10.5 (9.75 to 11.62) | 4651 (3696 to 5932) | 27.95 (22.29 to 35.53) | 1.84 (0.98 to 2.71) |
| United Republic of Tanzania | 665073 (363287 to 1142962) | 11664.66 (6254.59 to 20229.84) | 862106 (483222 to 1410462) | 6420.76 (3595.61 to 10498.08) | -4.29 (-5.87 to -2.69) |
| United States of America | 161700 (155376 to 170664) | 225.59 (216.6 to 238.37) | 59696 (49237 to 73771) | 73.54 (60.67 to 90.86) | -5.79 (-6.45 to -5.13) |
| United States Virgin Islands | 99 (98 to 101) | 336.9 (332.86 to 343.09) | 49 (48 to 51) | 264.85 (257.94 to 275.09) | -2.23 (-3.2 to -1.26) |
| Uruguay | 546 (534 to 567) | 72.82 (71.2 to 75.65) | 2095 (1939 to 2329) | 244.06 (225.84 to 271.23) | 3.16 (2.25 to 4.08) |
| Uzbekistan | 2245 (2215 to 2279) | 50.92 (50.3 to 51.61) | 10529 (10033 to 11346) | 112.89 (107.66 to 121.52) | 2.47 (1.98 to 2.96) |
| Vanuatu | 4 (1 to 9) | 12.96 (3.09 to 28.14) | 342 (157 to 614) | 457.38 (211.43 to 813.87) | 10.93 (10.23 to 11.64) |
| Venezuela (Bolivarian Republic of) | 4890 (4845 to 4947) | 106.51 (105.54 to 107.73) | 20081 (19350 to 21109) | 278.41 (268.01 to 293.03) | 3.73 (3.1 to 4.35) |
| Viet Nam | 2520 (1204 to 4358) | 13.88 (6.5 to 24.31) | 41842 (25822 to 70757) | 155.86 (97.3 to 260.8) | 7.4 (5.17 to 9.67) |
| Yemen | 166 (37 to 436) | 6.59 (1.4 to 17.23) | 5709 (1960 to 11304) | 72.47 (24.95 to 142.47) | 8.58 (7.75 to 9.41) |
| Zambia | 323814 (178584 to 548645) | 18199.79 (9609.12 to 31442.06) | 578310 (306905 to 1002263) | 12972.52 (6862.92 to 22506.22) | -4.04 (-5.63 to -2.42) |
| Zimbabwe | 491688 (270025 to 825650) | 21047.09 (11137.38 to 35981.19) | 519366 (292914 to 858159) | 13986.8 (7902.68 to 23076.15) | -4.4 (-6.4 to -2.36) |

**Supplementary Figure 1**

The numbers and rates of incidence, prevalence, mortality, and DALYs for HIV/AIDS among women of childbearing age by SDI regions in 1990 and 2021


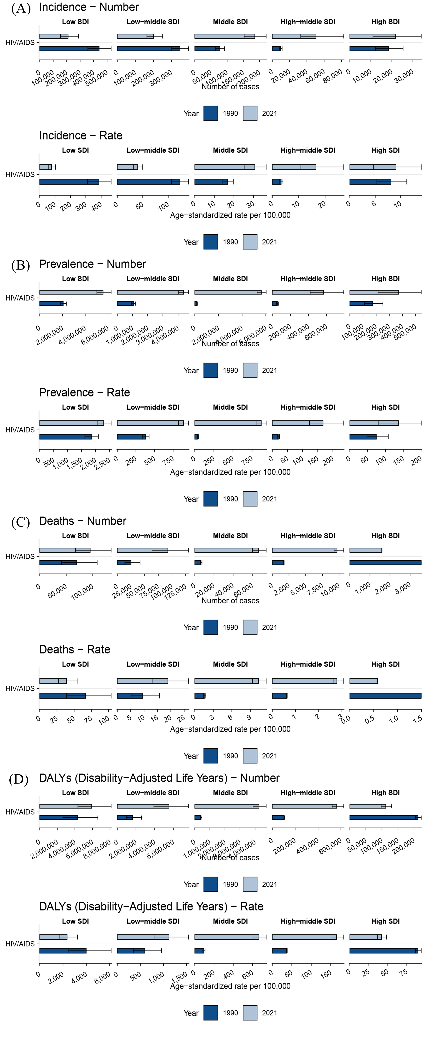


**Supplementary Figure 2**

The rates of incidence, prevalence, mortality, and DALYs for HIV/AIDS among women of childbearing age in different age sex groups


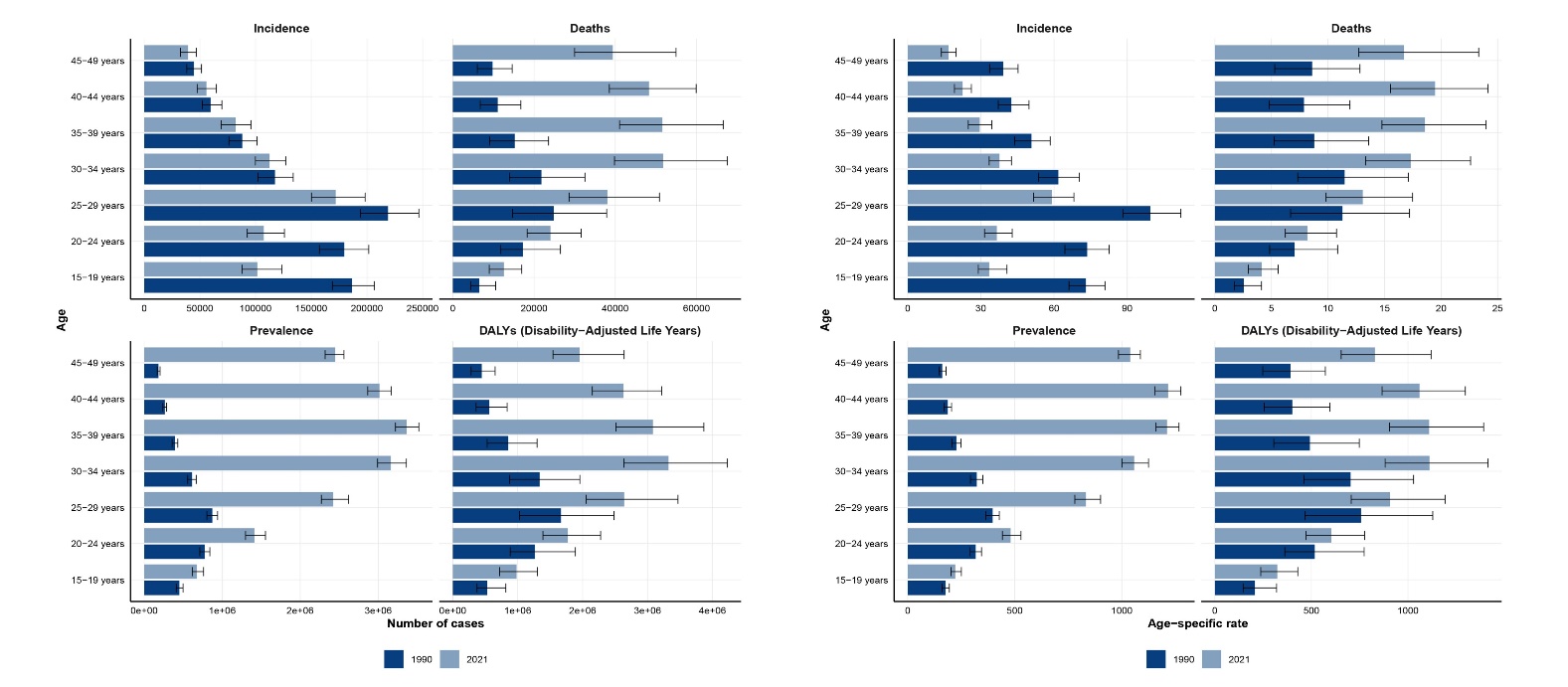


**Supplementary Figure 3**

Decomposition analysis of incidence, prevalence, mortality, and DALYs change in HIV/AIDS among women of childbearing age from 1990 to 2021 at the global level and by SDI.


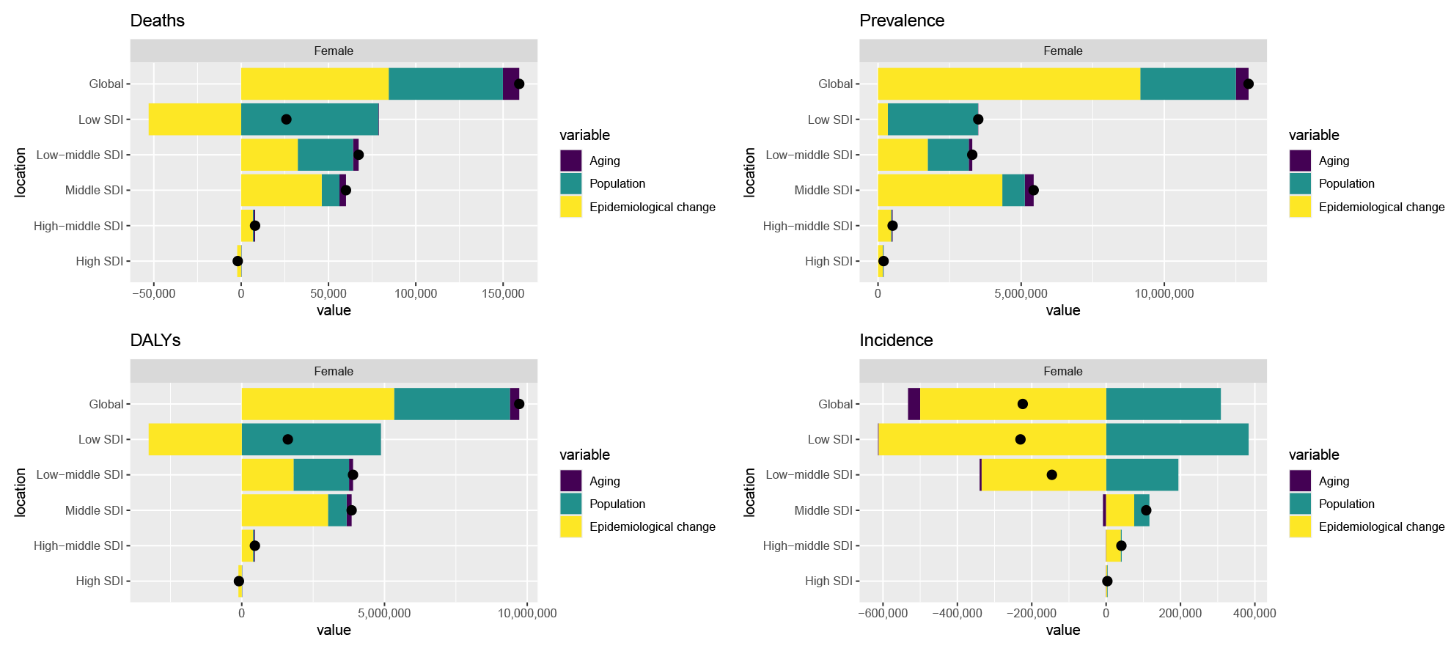


**Supplementary Figure 4**

Joinpoint regression analysis of the temporal trends of HIV/AIDS among women of childbearing age from 1990 to 2021.


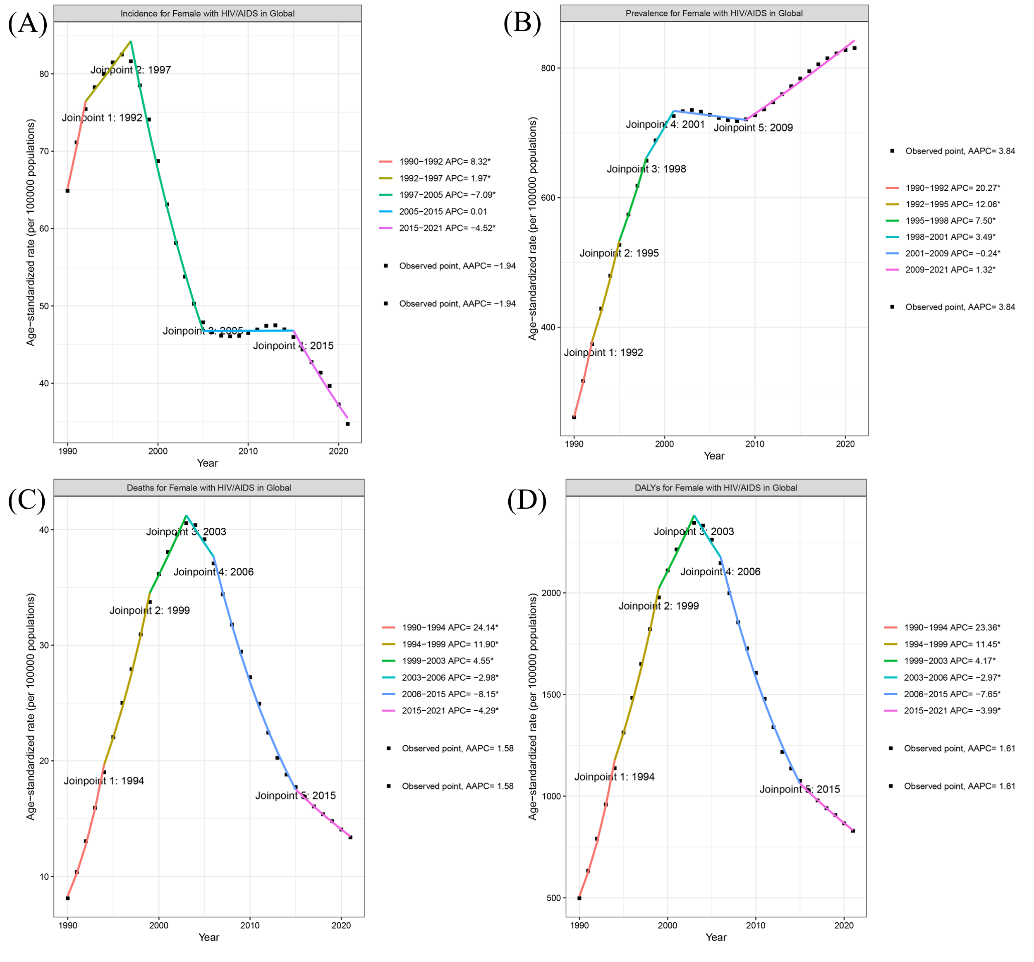


**Supplementary Figure 5**

Prediction of disease burden for HIV/AIDS among women of childbearing age by 2046.


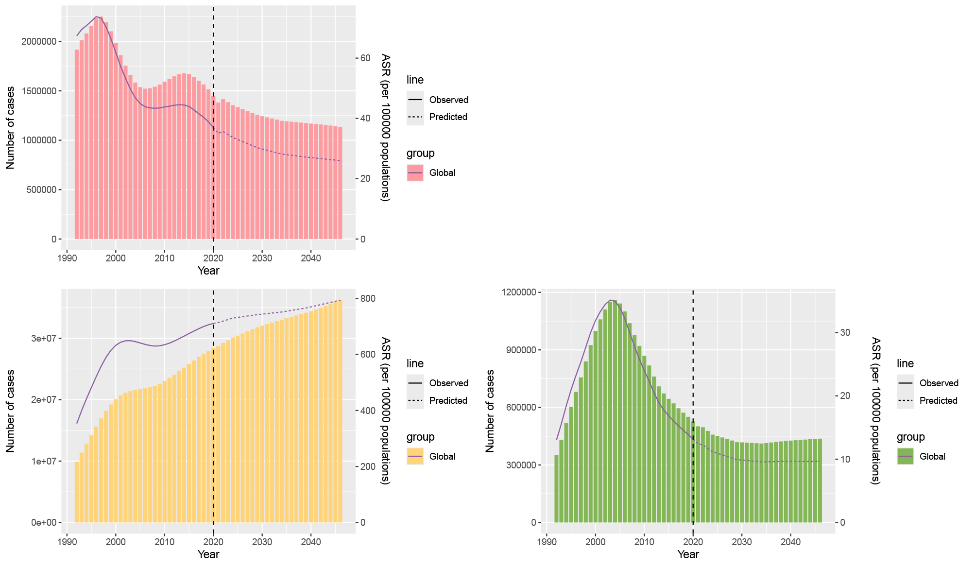

Supplement: Supplementary file 1 [file Table_1.docx]
